# Isotope-assisted metabolic flux analysis as an equality-constrained nonlinear program for improved scalability and robustness

**DOI:** 10.1371/journal.pcbi.1009831

**Published:** 2022-03-24

**Authors:** Daniel J. Lugar, Ganesh Sriram

**Affiliations:** Department of Chemical and Biomolecular Engineering, University of Maryland, College Park, Maryland, United States of America; The Pennsylvania State University, UNITED STATES

## Abstract

Stable isotope-assisted metabolic flux analysis (MFA) is a powerful method to estimate carbon flow and partitioning in metabolic networks. At its core, MFA is a parameter estimation problem wherein the fluxes and metabolite pool sizes are model parameters that are estimated, via optimization, to account for measurements of steady-state or isotopically-nonstationary isotope labeling patterns. As MFA problems advance in scale, they require efficient computational methods for fast and robust convergence. The structure of the MFA problem enables it to be cast as an equality-constrained nonlinear program (NLP), where the equality constraints are constructed from the MFA model equations, and the objective function is defined as the sum of squared residuals (SSR) between the model predictions and a set of labeling measurements. This NLP can be solved by using an algebraic modeling language (AML) that offers state-of-the-art optimization solvers for robust parameter estimation and superior scalability to large networks. When implemented in this manner, the optimization is performed with no distinction between state variables and model parameters. During each iteration of such an optimization, the system state is updated instead of being calculated explicitly from scratch, and this occurs concurrently with improvement in the model parameter estimates. This optimization approach starkly contrasts with traditional “shooting” methods where the state variables and model parameters are kept distinct and the system state is computed afresh during each iteration of a stepwise optimization. Our NLP formulation uses the MFA modeling framework of Wiechert et al. [1], which is amenable to incorporation of the model equations into an NLP. The NLP constraints consist of balances on either elementary metabolite units (EMUs) or cumomers. In this formulation, both the steady-state and isotopically-nonstationary MFA (inst-MFA) problems may be solved as an NLP. For the inst-MFA case, the ordinary differential equation (ODE) system describing the labeling dynamics is transcribed into a system of algebraic constraints for the NLP using collocation. This large-scale NLP may be solved efficiently using an NLP solver implemented on an AML. In our implementation, we used the reduced gradient solver CONOPT, implemented in the General Algebraic Modeling System (GAMS). The NLP framework is particularly advantageous for inst-MFA, scaling well to large networks with many free parameters, and having more robust convergence properties compared to the shooting methods that compute the system state and sensitivities at each iteration. Additionally, this NLP approach supports the use of tandem-MS data for both steady-state and inst-MFA when the cumomer framework is used. We assembled a software, eiFlux, written in Python and GAMS that uses the NLP approach and supports both steady-state and inst-MFA. We demonstrate the effectiveness of the NLP formulation on several examples, including a genome-scale inst-MFA model, to highlight the scalability and robustness of this approach. In addition to typical inst-MFA applications, we expect that this framework and our associated software, eiFlux, will be particularly useful for applying inst-MFA to complex MFA models, such as those developed for eukaryotes (e.g. algae) and co-cultures with multiple cell types.

## Author summary

Isotope-assisted metabolic flux analysis (MFA) is a computationally intensive parameter estimation problem. Isotopically nonstationary MFA (inst-MFA) represents the most computationally burdensome MFA application. We present the formulation of the steady-state and inst-MFA problems as equality-constrained nonlinear programs (NLPs), solved by a state-of-the-art solver implemented in an algebraic modeling language. We show that this formulation leads to robust convergence properties compared to traditional approaches, particularly for inst-MFA. We developed a software, eiFlux that uses the NLP formulation to perform both steady-state and inst-MFA. We demonstrate the application of eiFlux on several examples, including a genome-scale inst-MFA model, and show that it has robust optimal convergence even when started from a very poor initial guess for the parameters. eiFlux is implemented using the Python programming language and the General Algebraic Modeling System (GAMS), using the CONOPT solver. eiFlux is available upon request, pending institutional approval, and is free for academic use.

This is a *PLOS Computational Biology* Methods paper.

## Introduction

Stable isotope-assisted metabolic flux analysis (MFA) is a powerful methodology used for experimentally quantifying *in-vivo* metabolic fluxes [2–4]. This methodology involves two parts—experimental data collection and computational flux estimation. Experimentally, an isotopically labeled substrate is fed to a cell culture. Labeling measurements of intracellular metabolites, which depend on the fluxes are then measured, typically by using mass spectrometry (MS). A metabolic network model of carbon atom rearrangements is then fit to the measured labeling patterns, called mass isotopomer distributions (MIDs), to estimate the flux distribution that best accounts for these measurements. The labeling measurements can be collected under either steady-state labeling conditions or unsteady-state (isotopically nonstationary) labeling conditions in which time-series measurements are gathered on the approach to steady state. In the steady-state case, an algebraic model, in which the fluxes are unknown parameters, is fit to the labeling data. In the isotopically nonstationary case, the model consists of a system of ordinary differential equations (ODEs) in which the fluxes and metabolite pool sizes are the unknown parameters. This is a computationally intensive parameter estimation problem that requires the use of specialized software.

Several modeling formulations, especially those involving cumomers [1] and elementary metabolite units (EMUs) [5], have been applied to ease the computational burden by casting the model equations in a way that they can be solved in a piecewise linear form. Other formulations that seek to reduce computational burden by expressing the state variables in an alternative form include bondomers [6,7], (which are useful for a specific type of MFA involving a single, uniformly labeled carbon source) and fluxomers [8]. Formulations such as these have resulted in the steady-state MFA problem becoming efficiently solvable. The extension of the EMU method to isotopically nonstationary MFA (inst-MFA) has made the unsteady-state problem computationally tractable [9].

MFA applications estimate fluxes by solving a parameter estimation problem that minimizes the sum of squared residuals (SSR) between the experimentally measured and model-predicted labeling state (or dynamic labeling state [DLS] in case of inst-MFA). This minimization requires an optimization algorithm to estimate the optimal fluxes (and metabolite pool sizes for inst-MFA) that best account for the experimental DLS. To our knowledge, all available inst-MFA software employ the “shooting method” for determining the optimum. In this method, parameter estimation is performed via a sequential algorithm, in which a set of model parameters are chosen and the DLS is subsequently evaluated by using a numerical integration scheme for that set of parameter values. The parameter estimates are refined at each iteration using a Gauss-Newton method (e.g. Levenberg-Marquardt) or a derivative-free method such as simulated annealing [10,11], and the DLS is recalculated and compared to the measurements. These steps are repeated until an optimum is reached.

A desirable quality of a parameter estimation process such as MFA is *robustness*. A robust estimation would be accurate (converging in the neighborhood of the global optimum) and reliable (converging to the same optimum from arbitrary starting points) [12]. The shooting method, which re-computes the DLS at each iteration, is not robust because it suffers from a small region of convergence [13], particularly for problems with a large number of free parameters. This is typically due to the presence of many local optima, often far from the globally optimal solution. In this case, for a general network, robust convergence to the global or a near-global optimum typically requires a very good initial guess for the model parameters. However, in inst-MFA the pool sizes and fluxes are generally difficult to estimate *a priori*, and a good initial guess is usually not available for the large number of free parameters in this problem. Therefore, the optimization must typically be restarted many times from various points in the feasible parameter space to increase the chance of finding the globally optimal solution.

To address these limitations, we present the formulation of the MFA problem as an equality-constrained nonlinear program (NLP). In the NLP formulation, the EMU or cumomer balances constitute a system of equality constraints whose feasibility must be maintained while minimizing the error between the measurements and the model predictions. For inst-MFA, we use collocation [14] to discretize the system of ordinary differential equations (ODEs) to a form amenable to state-of-the-art NLP solvers. This discretization is commonly referred to as transcription [14]. In the NLP approach, the parameter estimation and state prediction occur simultaneously during optimization, contrasting with traditional methods that compute the state from scratch using the parameter values at each iteration. The NLP problem is solved efficiently using the NLP solver CONOPT [15,16] implemented in the General Algebraic Modeling System (GAMS) [17], an algebraic modeling language (AML). We employ the cumomer modeling formulation presented by Wiechert et al. [1], which is ideally suited for incorporating the model equations into an NLP framework. Further, we also use this modeling formulation to develop the EMU balance equations. In this formulation, the EMU fractional abundances are the state variables rather than the EMU vectors as has traditionally been the case [5,9]. Solving the NLP using a solver implemented on an AML such as GAMS results in efficient and robust solution of the MFA and inst-MFA problems. AMLs have capabilities such as automatic differentiation and efficient use of sparse linear algebra. They therefore efficiently integrate state-of-the-art optimization algorithms and render them amenable for use on large-scale problems.

We show that the NLP formulation is particularly beneficial for inst-MFA, where collocation is used to transcribe the ODE system, resulting in robust convergence and relatively few local optima even for large network models. This contrasts with traditional methods which use the shooting method for inst-MFA parameter estimation and tend to suffer from a small region of convergence. The NLP formulation also scales well to problems with a large number of independent parameters [10], providing a computationally effective framework for flux estimation on large network models with many independent fluxes and pool sizes, such as those encountered in models of eukaryotic systems (e.g. algae), co-culture conditions, or those of genome-scale. Additionally, because isotopomers and cumomers are easily mapped to tandem MS measurements [18], this formulation supports the use of tandem MS data for both steady-state and inst-MFA.

**Fig 1:**
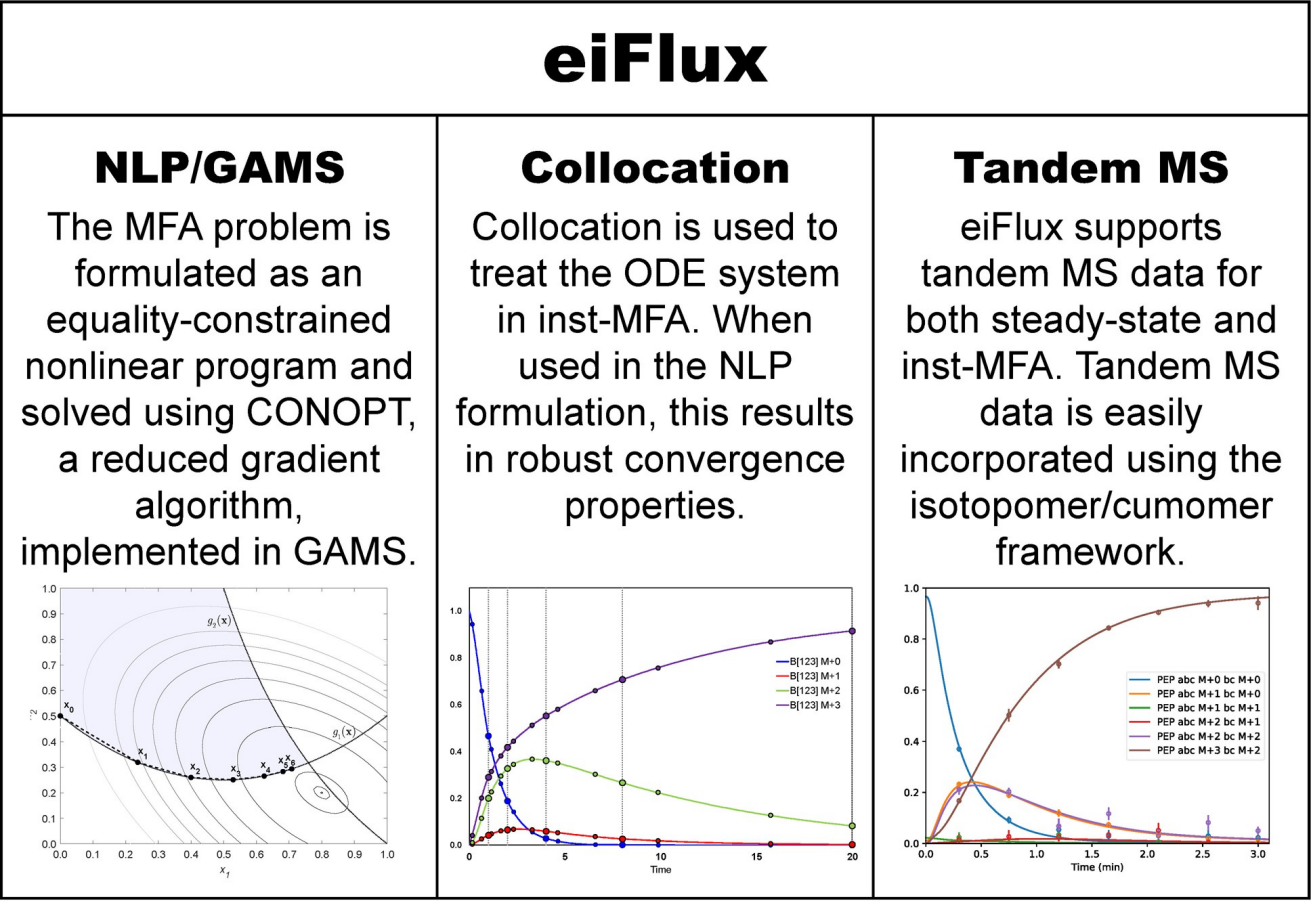
Several factors make eiFlux unique among currently available MFA software. eiFlux uses equality-constrained nonlinear programming to solve both the steady-state and inst-MFA problems. Treating the ODE system using collocation in an NLP formulation results in robust convergence properties. eiFlux, which supports the cumomer modeling approach, may directly fit tandem MS data.

We have developed a software, eiFlux (**e**quality-constrained nonlinear programming for **i**sotope-assisted metabolic **flux** analysis), that uses the NLP formulation and supports both steady-state and inst-MFA (**Fig 1**). Using the GAMS Python API, eiFlux assembles the matrices and vectors for the model equations of a given metabolic network using the Python programming language, then solves the MFA problem in GAMS using the NLP solver CONOPT, enforcing the model equations as equality constraints. The metabolic network and carbon atom rearrangements are specified in a user-friendly manner similar to other software, making models easily transferable between eiFlux and existing software.

## Methods

### Steady-state MFA as an equality-constrained nonlinear program

To perform steady-state MFA on an arbitrary metabolic network, the cumomer or EMU balances, the measurement mapping matrix, and SSR objective function should be assembled into a general model structure. We first present how this model structure is developed for the cumomer balances, and then we discuss how the EMU framework also fits this structure. We employed the systematic procedure developed by Wiechert et al. [1], which enables a metabolic network and its associated carbon atom rearrangements to be efficiently translated into a system of cumomer balances. Möllney et al. [19] describe how isotopomer and cumomer distributions are mapped to experimental data sets using a mapping matrix. The NLP for the steady-state MFA problem, based on this formulation, is shown in (Eq 1).


$$\begin{array}{l} \min\;z=\sum_{n}{\frac{\left(h_{n}^{meas}m_{n}^{meas}-m_{n}\right)}{\sigma_{n}^{2}}}^{2}+\sum_{r}\frac{{\left(v_{r}^{meas}-v_{r}\right)}^{2}}{\sigma_{v,r}^{2}}\\ \mathrm{cumomer}\;\mathrm{or}\;\mathrm{EMU}\;\mathrm{balances}\\ \begin{array}{llll} s.t. & \sum_{r}v_{r}(\frac{1}{2}\sum_{i,j}Q_{rkij}x_{i}x_{j}+\sum_{j}P_{rkj}x_{j}+\sum_{j}P_{rkj}^{inp}x_{j}^{inp})=0 & \forall k & \begin{array}{l} Q_{rkij}x_{i}x_{j}:\;\mathrm{bimolecular}\;\mathrm{reactions}\;\mathrm{producing}\;x_{{}_{k}}\\ P_{rkj}x_{j}:\;\mathrm{unimolecular}\;\mathrm{production}\;\mathrm{and}\;\mathrm{consumption}\;\mathrm{of}\;x_{{}_{k}}\;\\ \begin{array}{l} P_{rkj}^{inp}x_{j}^{inp}:\;\mathrm{input}\;\mathrm{of}\;\mathrm{fed}\;\mathrm{isotopically}\;\mathrm{labeled}\;\mathrm{compound}(s)\\ \mathrm{into}\;\mathrm{the}\;\mathrm{network} \end{array} \end{array}\\ & \sum_{r}S_{qr}v_{r}=0 & \forall q & \mathrm{fluxes}\;\mathrm{are}\;\mathrm{stoichiometrically}\;\mathrm{constrained}\\ & \sum_{r}R_{wr}v_{r}\leq b_{w} & \forall w & \mathrm{linear}\;\mathrm{inequality}\;\mathrm{constraints}\;\mathrm{on}\;\mathrm{fluxes}\\ & \sum_{c}U_{nc}h_{c}^{frag}=h_{n}^{meas} & \forall n & \begin{array}{l} \mathrm{constrains}\;\mathrm{measurement}\;\mathrm{scaling}\;\mathrm{factors}\;\mathrm{to}\\ \mathrm{equal}\;\mathrm{fragment}\;\mathrm{scaling}\;\mathrm{factors} \end{array}\\ & m_{n}=\sum_{j}M_{nj}x_{j} & \forall n & \mathrm{cumomer}\;\mathrm{or}\;\mathrm{EMU}\;\mathrm{mapping}\;\mathrm{to}\;\mathrm{measurements}\\ & x_{u}=1 & \forall u & \mathrm{all}\;\mathrm{zero}‐\mathrm{order}\;\mathrm{cumomers}\equiv 1\;(\mathrm{only}\;\mathrm{for}\;\mathrm{cumomer}\;\mathrm{framework})\\ & v_{r}^{lb}\leq v_{r}\leq v_{r}^{ub} & \forall r & \mathrm{flux}\;\mathrm{lower}\;\mathrm{and}\;\mathrm{upper}\;\mathrm{bounds} \end{array} \end{array}$$ (1)

The objective function of the NLP (Eq 1) consists of (i) a term for the residuals between the measured isotope labeling data and the simulated data, for which *n* is the measurement index; and (ii) a term for the residuals between any measured fluxes and the computed fluxes, in which *r* is the flux index. Here, *σ*_*n*_ and *σ*_*v*,*r*_ are the standard deviations for the isotope labeling measurements and the flux measurements, respectively. The equality constraints are (i) the cumomer or EMU balances, where *i*, *j*, and *k* are cumomer or EMU indices, and *Q*_*rkij*_, *P*_*rkj*_, and $P_{rkj}^{inp}$ are arrays defining the balances for the network’s carbon atom rearrangements, as developed by Wiechert et al. [1]; (ii) the stoichiometric flux balances, *S*_*qr*_ is the stoichiometric matrix; (iii) constraints on the (optional) measurement scaling factors [19] to equal their corresponding fragment scaling factors; (iv) the mapping of the cumomers or EMUs to the measurements; and (v) definition of zero-order cumomers if the cumomer framework is used. The inequality constraints include (i) any linear inequality constraints on the fluxes including bounds on the net fluxes of reversible reactions; and (ii) upper and lower bounds on fluxes. All flux values must be nonnegative, therefore for reversible reactions the forward reaction and the reverse reaction must each be considered as separate.

If the user is fitting complete and normalized MIDs, then these scaling factors should be fixed to equal 1. The elements of the matrix *U*_*nc*_ in this constraint are defined in (Eq 2).


$$U_{nc}=\{\begin{array}{l} 1\;\mathrm{if}\;\mathrm{measurement}\;n\;\mathrm{corresponds}\;\mathrm{to}\;\mathrm{measured}\;\mathrm{fragment}\;c\\ 0\;\mathrm{otherwise} \end{array}$$ (2)

This NLP may also be defined using the EMU framework. In this case, the EMU fractional abundances are the state variables rather than the cumomer fractions. Following the EMU decomposition presented by Antoniewicz et al. [5], the model arrays, *P*_*rkj*_, $P_{rkj}^{inp}$, and *M*_*nj*_ may be similarly defined. Storing the EMUs in the composite vector **x** (formed by concatenating the EMU vectors), the array *Q* is defined:

$$Q_{rkij}=\{\begin{array}{l} s\;\mathrm{if}\;i\neq j\;\mathrm{and}\;\mathrm{EMU}\;\mathrm{fractions}\;i\;\mathrm{and}\;j\;\mathrm{combine}\;\mathrm{to}\;\mathrm{form}\;\mathrm{EMU}\;\mathrm{fraction}\;k\;\mathrm{in}\;\mathrm{reaction}\;r\\ 2s\;\mathrm{if}\;i=j\;\mathrm{and}\;\mathrm{EMU}\;\mathrm{fractions}\;i\;\mathrm{and}\;j\;\mathrm{combine}\;\mathrm{to}\;\mathrm{form}\;\mathrm{EMU}\;\mathrm{fraction}\;k\;\mathrm{in}\;\mathrm{reaction}\;r\\ 0\;\mathrm{otherwise} \end{array}$$ (3)

Here *s* is the stoichiometric coefficient of the metabolite corresponding to the EMU fraction with index *k* in reaction with index *r*. The array *P*_*rkj*_ may similarly be defined as:

$$P_{rkj}=\{\begin{array}{l} s\;\mathrm{if}\;\mathrm{EMU}\;\mathrm{fraction}\;j\;\mathrm{produces}\;\mathrm{EMU}\;\mathrm{fraction}\;k\;\mathrm{in}\;\mathrm{reaction}\;r\\ -s\;\mathrm{if}\;k=j\;\mathrm{and}\;\mathrm{EMU}\;\mathrm{fraction}\;k\;\mathrm{is}\;a\;\mathrm{reactant}\;\mathrm{in}\;\mathrm{reaction}\;r\\ 0\;\mathrm{otherwise} \end{array}$$ (4)

The array $P_{rkj}^{inp}$, whose elements are nonzero for reactions in which a metabolite enters the network, is defined as follows:

$$P_{rkj}^{inp}=\{\begin{array}{l} s\;\mathrm{if}\;k=j\;\mathrm{and}\;\mathrm{EMU}\;\mathrm{fraction}\;k\;\mathrm{enters}\;\mathrm{the}\;\mathrm{network}\;\mathrm{in}\;\mathrm{reaction}\;r\\ 0\;\mathrm{otherwise} \end{array}$$ (5)

The measurement mapping matrix *M*_*nj*_ is defined as:

$$M_{nj}=\{\begin{array}{l} 1\;\mathrm{if}\;\mathrm{EMU}\;\mathrm{fraction}\;j\;\mathrm{corresponds}\;\mathrm{to}\;\mathrm{measured}\;\mathrm{EMU}\;\mathrm{fraction}\;n\\ 0\;\mathrm{otherwise} \end{array}$$ (6)

Last, **x**^*inp*^ is defined as the input composite EMU vector. For the fed isotopically labeled compound, the corresponding EMU elements are defined based on the pattern of isotope enrichment. The remaining EMU elements are defined based on natural isotope abundance.

In our implementation, this NLP is solved using the CONOPT reduced gradient algorithm, implemented in GAMS [15,16,20]. The CONOPT algorithm works well for optimization problems in which the total number of equality constraints is similar to the total number of variables. In each iteration of a reduced gradient algorithm, the variables are partitioned into two sets—basic and nonbasic. This partitioning is performed automatically by the solver without distinguishing between parameters and state variables. At each iteration of the algorithm, the nonbasic variables are propagated to a better point (one that improves the objective function), and the basic variables are propagated to the new point such that they satisfy the equality constraints [16,20]. Importantly, the number of basic variables is the same as the number of equality constraints. Therefore, the values of the basic variables are determined by these constraints, and any constraint violation is corrected for using iterations of Newton’s method within a given CONOPT solver iteration. There are approximately the same number of nonbasic variables as there are free parameters (fluxes, pool sizes, and scaling factors if used), so there are typically only *O*(100) nonbasic variables in a typical MFA problem formulated in this way. In CONOPT, second derivative information in the form of a reduced Hessian is computed only for the nonbasic variables for the optimization.

Therefore, for nonlinear programs in which the number of equality constraints is similar to the number of variables, there are fewer nonbasic variables, and the algorithm behaves more efficiently. A reduced gradient algorithm is well-suited for the MFA problem, in which a typical network has hundreds-to-thousands of cumomers or EMU fractional abundances, with each contributing an equality constraint. For a simple step-by-step example employing a generic reduced gradient algorithm to a simple example, see **S1 Text**. Importantly, a general NLP solver, such as CONOPT, does not distinguish model parameters (i.e. fluxes) from model variables (i.e. cumomers and simulated measurements) and views both as optimization variables.

**Fig 2:**
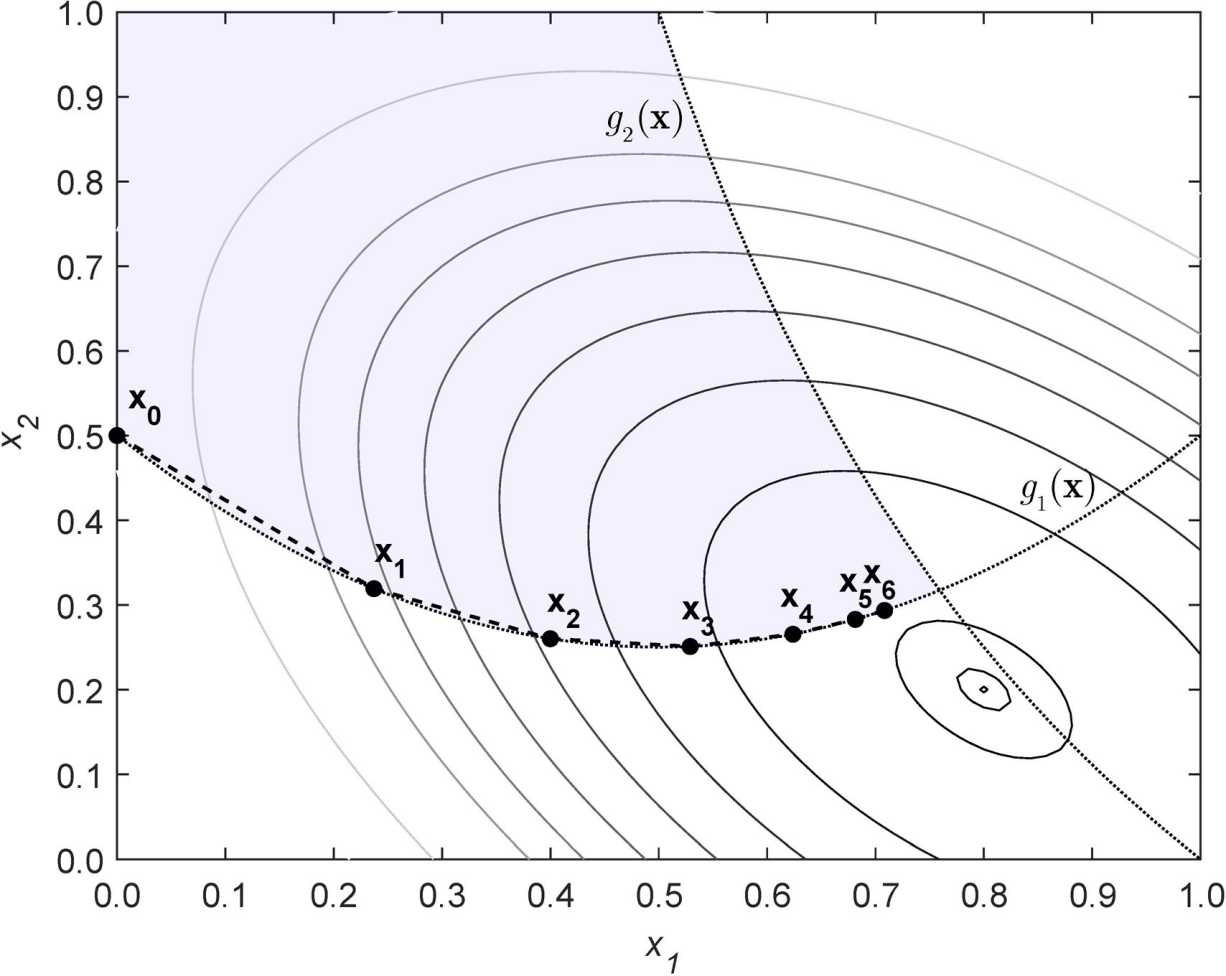
The first six iterations of a generic reduced gradient algorithm are shown for a simple illustrative example (see S1 Text for details). At each iteration of a reduced gradient algorithm, the variables are propagated to an improved point in which the constraints remain feasible. In this example, the feasible region (shaded in blue) lies above constraint g_1_ and below constraint g_2_. In this case, the iterations proceed along the g_1_ nonlinear constraint boundary.

Consequently, the cumomer or EMU balances are not solved from scratch at each iteration and are not solved in the previously mentioned piecewise-linear way. The algorithm is initialized at a random or user-input feasible flux distribution. From here, the algorithm moves from point to point in a direction such that the constraints, of which the cumomer or EMU balances are a large subset, remain statistically feasible (to a small tolerance). As the iterations proceed, the variable values are updated to maintain constraint feasibility. We illustrate this approach with an example NLP, where the iteration trajectory to the optimum can be easily visualized (**Fig 2**). **S1 Text** provides details on the NLP solved in this example. Here, *g*_1_(**x**) and *g*_2_(**x**) are nonlinear constraints. The algorithm begins at an initial feasible point, **x**_0_. From here, it proceeds through successive candidate points in a manner such that the constraints *g*_1_(**x**) and *g*_2_(**x**) remain feasible and the objective function is improved until the optimum is reached. The optimum is a minimal value of the objective function in which these constraints are satisfied, which is typically different from the unconstrained minimum.

CONOPT is able to effectively handle nonlinear constraints during each iteration of the optimization while taking reasonably large step sizes. This is because as a reduced gradient solver, CONOPT uses the equality constraints to reduce the effective problem size significantly. Recall that the equality constraints result from the EMU or cumomer balances, and the number of parameters is *O*(10^2^) for a typical model. The total number of variables in the NLP is therefore only a few hundred more than the total number of equality constraints. The CONOPT solver chooses a set of O(10^2^) variables (nonbasic) with the remaining variables (basic) being exactly determined based on the equality constraints. This effectively reduces the problem to one in only the nonbasic variables, of which there are only *O*(10^2^). The values of the basic variables, which number the same as the constraints, are updated using Newton iterations involving the inverse of the constraint Jacobian at each iteration until the equality constraints are satisfied within a tolerance. The default values of the maximum and minimum feasibility tolerances in CONOPT are 1x10^-7^ and 4x10^-10^ respectively. Models solved in eiFlux use these default values. In this way, the CONOPT solver is able to maintain feasibility for a large number of equality constraints during each iteration of the optimization problem with an effective size of O(10^2^) variables. In practice, MFA models solved using CONOPT very rarely lose feasibility during the optimization iterations. In iterations where feasibility is lost, the infeasibility is usually very small, and CONOPT is able to quickly regain feasibility by moving to a nearby feasible point. For mathematical details on CONOPT’s method of maintaining constraint feasibility, see **S2 Text**.

### Inst-MFA as an equality-constrained nonlinear program

In inst-MFA, metabolic fluxes are estimated using time-series measurements of isotope labeling in intracellular metabolites. In this case, the system is not at isotope labeling steady state, and the flux information is extracted from the time-dependent labeling dynamics [3,4]. This differs from steady-state MFA, in which flux estimates are extracted from steady-state labeling data. While the goal is the same—to accurately estimate intracellular fluxes using isotope labeling data—inst-MFA requires fitting a system of ODEs to data, rather than algebraic equations as is done in the steady-state case. This poses an added computational burden. Additionally, both fluxes and metabolite pool sizes are model parameters in inst-MFA. This contrasts with steady-state MFA, which is insensitive to pool sizes.

Inst-MFA may be preferred over steady-state MFA in a variety of situations. For a steady-state labeling experiment, the cell doubling time may be considered a lower bound for the time required to reach steady-state labeling in intracellular metabolites [4]. When the doubling time is long, as is common notably for mammalian cells, maintaining metabolic steady state in this time interval may be difficult. Because inst-MFA only requires the system to maintain metabolic steady state, the time length of the experiment may be considerably shorter, surmounting some experimental difficulties [3].

Photoautotrophic metabolism is an important situation where inst-MFA is not only preferred over steady-state MFA but is required. In this case, steady-state labeling profiles provide no information on metabolic fluxes [3,4]. All the flux information is contained within the labeling transient between the time when the cell culture is first provided the ^13^C-labeled carbon source and the time when it reaches isotope labeling steady state. Successfully implementing inst-MFA on these systems can provide vital engineering insights on photosynthetic organisms, such as algae.

The first mathematical treatment of inst-MFA [21,22] presented a method for simulating the DLS experiment and parameter sensitivities by using the cumomer framework. Subsequent work [23] demonstrated that these calculations can be accelerated by employing the adjoint approach to compute the gradient of the measurement residuals. Currently, two software are capable of performing inst-MFA—INCA [24] and OpenMebius [25]. All of these approaches simulate the DLS at every iteration of an optimization routine. For example, INCA uses the isotopically nonstationary EMU method [9,24] for DLS simulation within a Levenberg-Marquardt algorithm for parameter estimation. Casting the inst-MFA problem as an equality-constrained NLP would offer many benefits including robustness and scalability. However, this requires that the system of ODEs in inst-MFA be converted to algebraic equations.

Toward developing such a formulation, we constructed a framework that uses collocation [14] to transcribe any inst-MFA problem into a set of algebraic constraints for incorporation into a general NLP framework. As described by Shin et al. [10], there are several important benefits of this approach for large-scale parameter estimation problems with dynamic systems. Specifically, this approach allows large-scale models to be efficiently handled by an AML, such as GAMS, which employs automatic differentiation schemes for computing derivatives, and sparse linear algebra algorithms that efficiently handle the structured system of algebraic equations obtained by transcription of the ODEs [10]. Also, this approach scales well to problems with a large number of free parameters [10]. Because both pool sizes and free fluxes are model parameters in inst-MFA, the total number of parameters can easily exceed 100 when considering compartmented eukaryotic systems such as algae or genome-scale models. Approaches based on the traditional shooting method involving simulation of the sensitivity equations do not scale efficiently to problems with a large number of free parameters, impeding their usability for problems of this type [10,26]. In this context, the NLP framework provides a distinct advantage.

In addition to these considerations, the shooting method using a Gauss-Newton type (e.g. Levenberg-Marquardt) optimization algorithm does not have robust convergence when provided a poor initial guess for the parameter values [13]. In this situation, this method will often converge to poor local optima far from the global optimum. In inst-MFA, a good initial guess is typically not available for most or all of the fluxes and pool sizes in a model. This method can also have slow convergence in situations where the measurement residuals are large [27]. While local optima must be considered when using any convex optimization scheme on a nonconvex optimization problem (such as inst-MFA), the NLP framework using collocation has comparatively robust convergence properties [14], even when provided a poor initial guess for the parameters. To search for the global optimum, the optimization is typically restarted from a number of (random) initial starting points. This robust convergence means the user will need to perform fewer random restarts to find a global (or near-global) optimum compared to the shooting method. Alternatives such as derivative-free search methods, which among others include simulated annealing and genetic algorithms, can avoid local optima, but do not scale well to parameter estimation problems with a large number of free parameters [10], hindering their applicability to large MFA models.

The system of ODEs that describes the cumomer or EMU dynamics is shown in (Eq 7). This is analogous to the first constraint in (Eq 1) which is composed of the balances at steady state. The dynamics of each cumomer or EMU fraction, with index *k*, is described by an ODE. In this equation, *p*_*k*_ is the pool size of the metabolite corresponding to cumomer or EMU fraction *x*_*k*_. Together, the set of all balances constitute a system of coupled nonlinear ODEs.


$$p_{k}\frac{dx_{k}}{dt}=\sum_{r}v_{r}(\frac{1}{2}\sum_{i,j}Q_{rkij}x_{i}(t)x_{j}(t)+\sum_{j}P_{rkj}x_{j}(t)+\sum_{j}P_{rkj}^{inp}x_{j}^{inp})\;\forall k$$ (7)

In the NLP formulation, collocation is used to transcribe the ODEs [14]. Using collocation methods, the time domain is divided, and a polynomial approximates the ODE solution within each time interval. Transcribing the ODEs using collocation into a system of algebraic equations allows the parameter estimation problem to be solved using a general NLP solver (e.g. CONOPT). In eiFlux, we chose to use the Radau IIA orthogonal collocation method due to its stiff-stability properties [28,29]. Collocation methods also have corresponding fully implicit Runge-Kutta methods [28,29]. For a derivation of collocation methods and a detailed explanation of their connection to Runge-Kutta methods, see Huynh [29]. A simple example using the 5^th^-order Radau IIA collocation method is provided in **S3 Text**. For an ODE system of the form (Eq 8), the corresponding fully implicit Runge-Kutta Method requires solving (Eq 9) and (Eq 10) in each time interval.


$$\frac{dx}{dt}=f(x,t)$$ (8)

$$k_{i}=f(x(t_{k})+h\sum_{j=1}^{s}a_{ij}k_{j},t_{k}+c_{i}h)\;\forall i$$ (9)

$$x(t_{k}+h)=x(t_{k})+h\sum_{j=1}^{s}b_{j}k_{j}$$ (10)

In (Eq 9) and (Eq 10), *a*_*ij*_ refers to the elements of the Runge-Kutta matrix, each of which may be nonzero for a fully implicit method such as a Radau IIA method. The values of *c*_*i*_ are the collocation nodes on the interval [0,1], and *b*_*j*_ are the weights. The value of *s* is the number of stages in the method. The set indices used in transcribing the inst-MFA problem are summarized in **Table 1**.

**Table 1:** The indexed sets used in developing the NLP of the inst-MFA problem. The Greek letters are indices used in discretizing the ODE system.

Index	Set
*i*,*j*,*k*	Cumomer index
*u*	0-order cumomer index (subset of cumomers)
*r*	Flux index
*n*	Mass isotopomer distribution measurement index
*m*	Metabolite index
*q*	Row index of stoichiometric matrix
*w*	Flux inequality constraint index
*c*	Measured fragment index
*α*,*β*	Runge-Kutta stage index
*γ*	Time node index. Also the index for time interval (*t*_*γ*−1_,*t*_*γ*_].
*μ*	Measured time index

Choosing a set of time nodes $t_{0}<t_{1}<\ldots t_{\gamma }<t_{\gamma +1}<\ldots <t_{\gamma^{max}}$, the general Runge-Kutta scheme that discretizes the entire time domain is shown in (Eq 11) and (Eq 12). Here, *h*_*γ*_ = *t*_*γ*_−*t*_*γ*−1_.


$$k_{\alpha \gamma i}=f(x_{\gamma -1,i}+h_{\gamma }\sum_{\beta }a_{\alpha \beta }k_{\beta \gamma i},t_{\gamma -1}+c_{\alpha }h_{\gamma })\;\forall \alpha ,\gamma ,i$$ (11)

$$x_{\gamma i}=x_{\gamma -1,i}+h_{\gamma }\sum_{\alpha }b_{\alpha }k_{\alpha \gamma i}\;\forall \gamma ,i$$ (12)

Equation (Eq 11) can be split into two equations by introducing the variable *K*_*αγi*_.


$$K_{\alpha \gamma i}=x_{\gamma -1,i}+h_{\gamma }\sum_{\beta }a_{\alpha \beta }k_{\beta \gamma i}\;\forall \alpha ,\gamma ,i$$ (13)

$$k_{\alpha \gamma i}=f(K_{\alpha \gamma i},t_{\gamma -1}+c_{\alpha }h_{\gamma })\;\forall \alpha ,\gamma ,i$$ (14)

Applying (Eq 12), (Eq 13), and (Eq 14) to the instationary balances in (Eq 7) gives the following system of algebraic constraints for the transcribed ODE system:

$$\begin{array}{l} K_{\alpha \gamma i}=x_{\gamma -1,i}+h_{\gamma }\sum_{\beta }a_{\alpha \beta }k_{\beta \gamma i}\;\forall \alpha ,\gamma ,i\\ p_{k}k_{\alpha \gamma k}=\sum_{r}v_{r}(\frac{1}{2}\sum_{i,j}Q_{rkij}K_{\alpha \gamma i}K_{\alpha \gamma j}+\sum_{j}P_{rkj}K_{\alpha \gamma j}+\sum_{j}P_{rkj}^{inp}x_{j}^{inp})\;\forall \alpha ,\gamma ,k\\ x_{\gamma i}=x_{\gamma -1,i}+h_{\gamma }\sum_{\alpha }b_{\alpha }k_{\alpha \gamma i}\;\forall \gamma ,i \end{array}$$ (15)

To approximate the cumomer or EMU fraction values, *x*_*μi*_, at a time point *t*_*μ*_ where a measurement was taken within the time interval *γ*, the polynomial basis functions *B*_*α*_(*τ*) defined in (Eq 16) for the collocation method, are used to develop (Eq 17). These basis functions are found by integrating the Lagrange polynomials, $l_{\alpha }(\tau )$. For Radau collocation methods, these Lagrange polynomials are constructed from the zeros of the corresponding Radau polynomial, *c*_1_…*c*_*s*_ [28,29]. See **S3 Text** for an example using Radau collocation to approximate the solution to an ODE system. Equation S2.7 in **S3 Text** lists the basis functions *B*_*α*_(*τ*) explicitly for a 3-stage method.


$$B_{\alpha }(\tau )=\int_{0}^{\tau }l_{\alpha }(\tau^{\prime })d\tau^{\prime }=\int_{0}^{\tau }\left(\prod_{\beta =1,\alpha \neq \beta }^{s}\frac{\tau^{\prime }-c_{\beta }}{c_{\alpha }-c_{\beta }}\right)d\tau^{\prime }$$ (16)

$$x_{\mu i}=x_{\gamma -1,i}+h_{\gamma }\sum_{\alpha }B_{\alpha }(\tau_{\mu })k_{\alpha \gamma i},\;\tau_{\mu }=\frac{t_{\mu }-t_{\gamma -1}}{h_{\gamma }},\;t_{\gamma -1}<t_{\mu }\leq t_{\gamma }\;\forall \mu ,i$$ (17)

Together, (Eq 15) and (Eq 17) are used to generate an NLP with a transcribed ODE system (Eq 18). For reference, all indexed sets used in developing the NLP are defined in **Table 1**. For clarity, the sets used in transcribing the ODE system are represented using Greek letters.


$$\begin{array}{l} \min\;z=\sum_{n,\mu }{\frac{\left(h_{n\mu }^{meas}m_{n\mu }^{meas}-m_{n\mu }\right)}{\sigma_{n\mu }^{2}}}^{2}+\sum_{r}\frac{{\left(v_{r}^{meas}-v_{r}\right)}^{2}}{\sigma_{v,r}^{2}}+\sum_{m}\frac{{\left(p_{m}^{met,meas}-p_{m}^{met}\right)}^{2}}{\sigma_{p,m}^{2}}\\ \begin{array}{llll} s.t. & K_{\alpha \gamma i}=x_{\gamma -1,i}+h_{\gamma }\sum_{\beta }a_{\alpha \beta }k_{\beta \gamma i} & \forall \alpha ,\gamma ,i & \mathrm{Runge}‐\mathrm{Kutta}\;\mathrm{term}\;1\;(\mathrm{eqn}.\;14)\\ & p_{k}k_{\alpha \gamma k}=\sum_{r}v_{r}(\frac{1}{2}\sum_{i,j}Q_{rkij}K_{\alpha \gamma i}K_{\alpha \gamma j}+\sum_{j}P_{rkj}K_{\alpha \gamma j}+\sum_{j}P_{rkj}^{inp}x_{j}^{inp}) & \forall \alpha ,\gamma ,k & \mathrm{Runge}‐\mathrm{Kutta}\;\mathrm{term}\;2\;(\mathrm{eqn}.\;14)\\ & x_{\gamma i}=x_{\gamma -1,i}+h_{\gamma }\sum_{\alpha }b_{\alpha }k_{\alpha \gamma i} & \forall \gamma ,i & \mathrm{Runge}‐\mathrm{Kutta}\;\mathrm{term}\;3\;(\mathrm{eqn}.\;14)\\ & p_{k}=\sum_{m}D_{km}p_{m}^{met} & \forall k & \begin{array}{l} \mathrm{cumomer}/\mathrm{EMU}\;\mathrm{fraction}\;\mathrm{pool}\;\mathrm{sizes}=\\ \mathrm{metabolite}\;\mathrm{pool}\;\mathrm{sizes} \end{array}\\ & \sum_{r}S_{qr}v_{r}=0 & \forall q & \mathrm{fluxes}\;\mathrm{are}\;\mathrm{stoichiometrically}\;\mathrm{constrained}\\ & \sum_{r}R_{wr}v_{r}\leq b_{w} & \forall w & \mathrm{linear}\;\mathrm{inequality}\;\mathrm{constraints}\;\mathrm{on}\;\mathrm{fluxes}\\ & \sum_{c}U_{nc}h_{c\mu }^{frag}=h_{n\mu }^{meas} & \forall n,\mu & \begin{array}{l} \mathrm{constrains}\;\mathrm{measurement}\;\mathrm{scaling}\;\mathrm{factors}\;\mathrm{to}\\ \mathrm{equal}\;\mathrm{fragment}\;\mathrm{scaling}\;\mathrm{factors} \end{array}\\ & x_{\mu i}=x_{\gamma -1,i}+h_{\gamma }\sum_{\alpha }B_{\alpha }(\tau_{\mu })k_{\alpha \gamma i},\;\tau_{\mu }=\frac{t_{\mu }-t_{\gamma -1}}{h_{\gamma }},t_{\gamma -1}<t_{\mu }\leq t_{\gamma } & \forall \mu ,i & \begin{array}{l} \mathrm{cumomer}\;\mathrm{values}\;\mathrm{at}\\ \mathrm{measurement}\;\mathrm{times}\;(\mathrm{Eq}.\;16) \end{array}\\ & m_{n\mu }=\sum_{j}M_{nj}x_{\mu j} & \forall n,\mu & \begin{array}{l} \mathrm{map}\;\mathrm{cumomers}\;\mathrm{to}\\ \mathrm{measurements} \end{array}\\ & x_{\gamma u}=1 & \forall \gamma ,u & \mathrm{all}\;\mathrm{zero}‐\mathrm{order}\;\mathrm{cumomers}\equiv 1\;(\mathrm{only}\;\mathrm{for}\;\mathrm{cumomer}\;\mathrm{framework})\\ & x_{0i}=x_{i}^{inp} & \forall i & \mathrm{define}\;\mathrm{initial}\;\mathrm{conditions}\\ & v_{r}^{lb}\leq v_{r}\leq v_{r}^{ub} & \forall r & \mathrm{flux}\;\mathrm{bounds}\\ & p_{m}^{met,lb}\leq p_{m}^{met}\leq p_{m}^{met,ub} & \forall m & \mathrm{pool}\;\mathrm{size}\;\mathrm{bounds} \end{array}\; \end{array}$$ (18)

Here, the matrix **D** is used to constrain the pool sizes for all cumomers of the same metabolite to be equal (Eq 19). The vector of metabolite pool sizes, $p_{m}^{met}$, is thereby mapped to the vector of cumomer or EMU fraction pool sizes, *p*_*k*_ (Eq 19).


$$D_{km}=\{\begin{array}{l} 1\;\mathrm{if}\;\mathrm{cumomer}/\mathrm{EMU}\;\mathrm{fraction}\;k\;\mathrm{is}\;\mathrm{from}\;\mathrm{metabolite}\;m\\ 0\;\mathrm{otherwise} \end{array}$$ (19)

The initial conditions, *x*_0*i*_ are specified as being the input cumomers or EMU fractions, $x_{i}^{inp}$. In eiFlux, the fed label(s) are specified in **x**^*inp*^. Metabolites whose labeling patterns are not specified are assumed to have natural isotope abundance for each atom. Those whose labeling patterns are specified are assumed to have natural isotope abundance for atoms not specified as labeled. This definition of initial conditions allows for metabolites to have their initial labeling specified. This allows the flexibility to define an impulse input of an isotope label (as an alternative to the usual step input), which may be useful for modeling scenarios where a bolus of label was added to a culture. The cumomer or EMU values at the measured time points (having index *μ*), are mapped using the measurement mapping matrix M to the metabolite labeling measurements *m*_*nμ*_. The pool sizes, $p_{m}^{met}$ are constrained by user-defined upper and lower bounds. The objective function also contains a term for the measurement residuals of any measured pool sizes.

#### Support for tandem MS data

Tandem MS (MS/MS) is an extension of conventional “single” MS that provides greater information on the labeling state than single MS [30,30–32], thus potentially increasing the accuracy of flux estimations. In MS/MS (**S1 Fig**), analytes pass through two mass analyzers in series and undergo fragmentation between the two analyzers. The first mass analyzer selects for a specific mass of a parent ion, which is then fragmented to generate daughter ions with various masses. These daughter ions are analyzed in the second mass analyzer. Combined interpretation of the parent and daughter ion MIDs provides considerably richer information on the labeling state than single MS. A novel feature of eiFlux is that it supports tandem MS data for both steady-state MFA and inst-MFA. This feature is an advantage because MS/MS data are particularly useful for MFA [32].

Although most previous reports of MFA were based on gas chromatography (GC)-MS analysis, the use of liquid chromatography (LC)-MS/MS analysis is becoming more prevalent. Two distinct benefits of LC-MS/MS are that (i) it does not require derivatization of analytes and (ii) it can typically measure more intermediates of central carbon metabolism than GC-MS, especially those that derivatize inefficiently. Researchers utilizing LC-MS/MS for inst-MFA have often used only the parent ion measurements (MS data), as opposed to the parent and daughter ion measurements (MS/MS data) due to the current unavailability of an inst-MFA software to fit the MS/MS data.

## Results

### Toy network example

**Fig 3:**
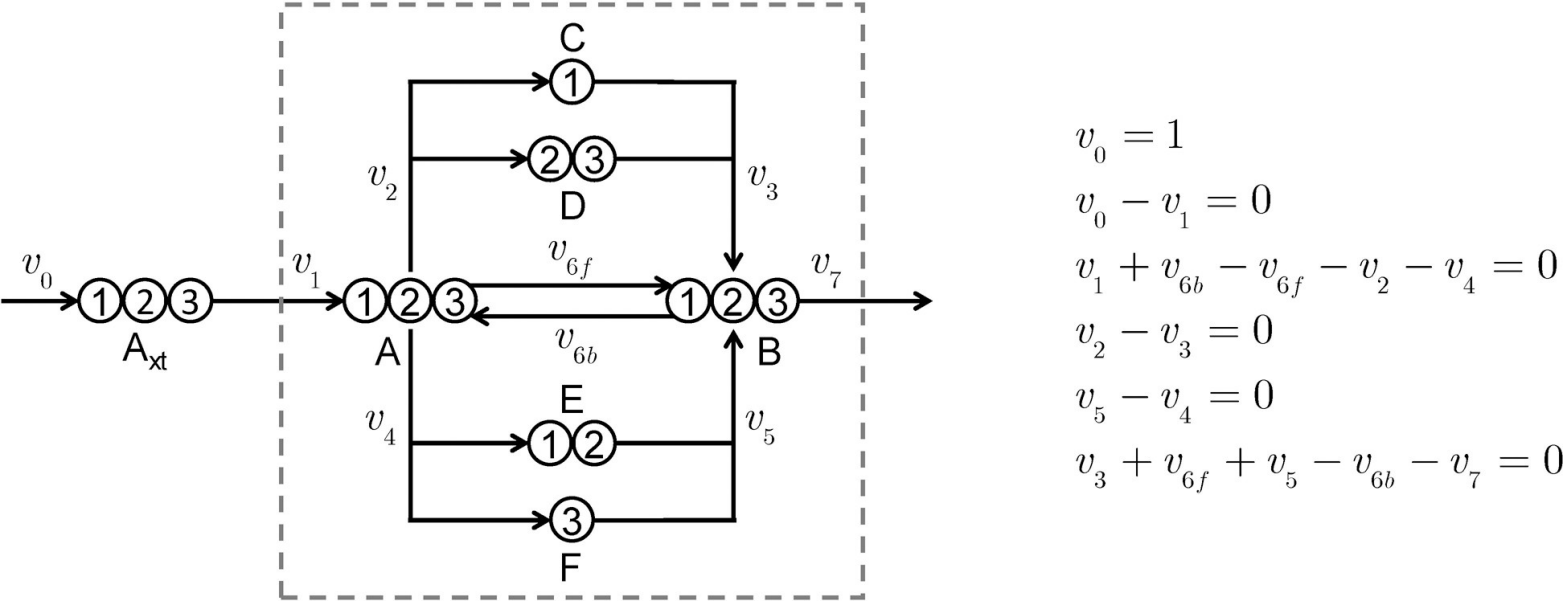
Toy network showing reactions and carbon atom rearrangements with stoichiometric flux constraints. The dashed gray line indicates the cell boundary. Since *v*_0_ is assumed to be known exactly, there are three independent fluxes based on the stoichiometric constraints. There are also six intracellular pool size parameters.

We present a simple example to illustrate how eiFlux uses collocation for inst-MFA flux and pool size estimation. For this example, we simulated the measurements from a given set of parameter (flux and pool size) values, and therefore the exact solution to the optimal estimation problem is known *a priori*. In this toy network (**Fig 3**), metabolite A may be converted to metabolite B via three different pathways, with one pathway being reversible. Each pathway has a unique carbon atom rearrangement. Suppose that at *t* = 0, the cell culture is fed media containing 100% U-^13^C A_xt_. The intracellular metabolites A, B, C, D, E, and F will gradually become enriched, becoming fully enriched as *t*→∞. In this case, all information on flux and pool sizes is contained within the labeling transient. Therefore, steady-state MFA is unusable and inst-MFA is necessary to estimate these parameters.

This toy network is simple enough that the isotopically nonstationary cumomer balances may be easily formulated and numerically integrated using MATLAB (MathWorks, Natick, MA). Therefore, to demonstrate the NLP approach to inst-MFA, we generated accurate synthetic data by numerically integrating the cumomer balances with set parameter values. For this demonstration, the chosen parameter values are shown in **S4 Text**. The synthetic data was generated for this toy network assuming no natural ^13^C abundance and 100% ^13^C enrichment of A_xt_ at *t* = 0. The synthetic measurements were taken from the synthetic data in the time interval [0 20] in time increments of 2. These synthetic measurements were used for solving the inverse problem—estimating the fluxes and pool sizes from the synthetic time-series measurements of fragments B[12], B[23], and B[123]. For details on the fluxes and pool sizes used to simulate the data, the solution converged on by eiFlux, and model fits to the data, see **S4 Text**. **S1 Data** contains the simulated data fit to this model.

**Fig 4:**
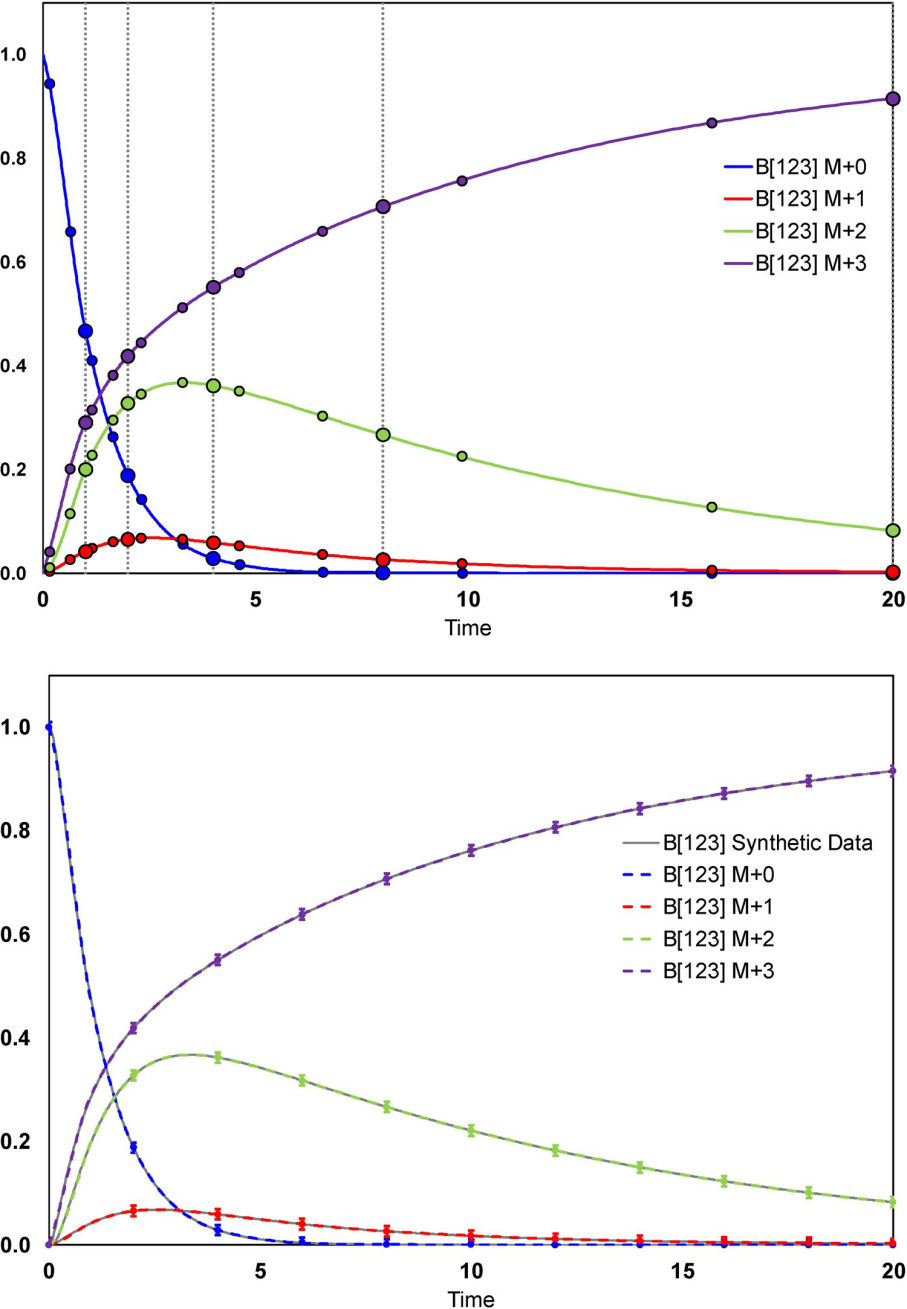
(Top) Continuous approximation to the DLS generated by fitting the synthetic measurements. The markers indicate the collocation points of the 5^th^-order Radau IIA method, with the larger markers indicating the endpoint of each time interval (which are also collocation points in Radau IIA methods). The vertical dashed lines indicate the boundaries of each time interval. (Bottom) The fit to the synthetic data is shown, with a standard deviation of 0.01 arbitrarily assigned to each synthetic measurement. The synthetic data was generated by numerically solving the cumomer balances with a known flux and pool size distribution using the 4^th^-order explicit Runge-Kutta method and a time step of 0.02. The continuous approximation generated by the 5^th^-order Radau IIA collocation method fitting the synthetic measurements is superimposed for comparison. Clearly, using the collocation method the synthetic measurements are accurately fit by the model, and the continuous approximation accurately matches the synthetic data.

The resulting time-dependent labeling profile of metabolite B is shown in **Fig 4**. The 5^th^-order Radau IIA collocation points in each time interval are displayed as markers. In this example, the time domain was split into five intervals with three collocation points in each interval. The point at *t* = 0 represents the initial condition. Using the NLP formulation of inst-MFA, the synthetic measurements are accurately fit to the model. Additionally, the continuous approximation generated by the 5^th^-order Radau IIA method accurately matches the synthetic data generated by solving the cumomer balances in MATLAB.

The inst-MFA problem consists of a system of ODEs, many of which are stiff. For a typical labeling experiment, it is known by the nature of the problem that the stiff region is located at the beginning of the time span. It is important to assign short time intervals early in the experiment’s time span so that the stiff dynamics may be accurately captured. If the time interval assigned encompassing the stiff region of the ODEs is too wide, the user may notice numerical artifacts in the polynomial approximation within this time interval. However, due to the stiff stability properties of the Radau IIA methods, the numerical solution remains stable in the subsequent intervals.

**Fig 5:**
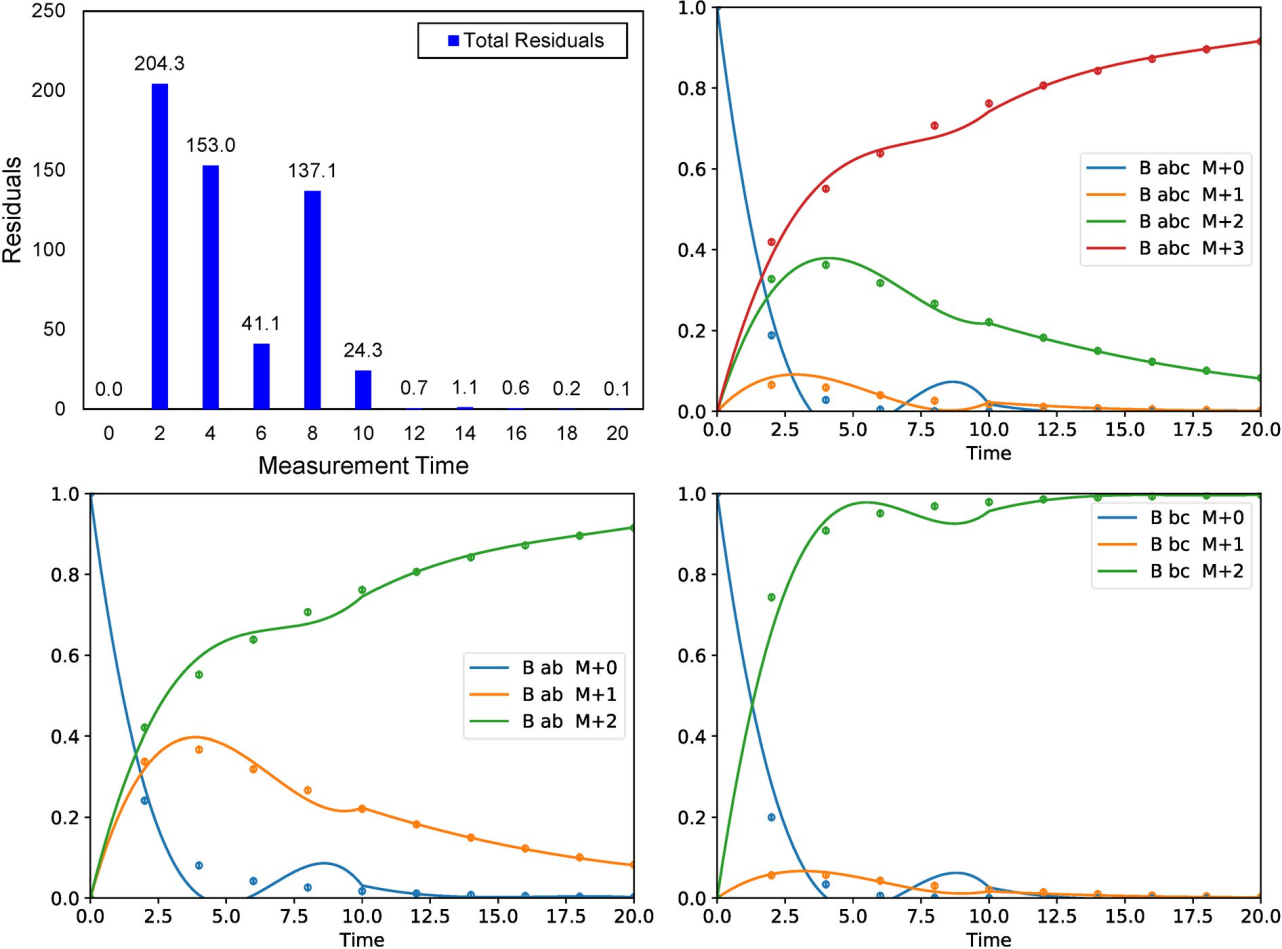
The result of assigning a larger than permissible step size in the stiff region for the toy network. The total measurement residuals converged upon by eiFlux when the time domain is divided into two intervals: [0, 10] and [10, 20] is plotted showing that the poor approximation in the first time interval does not significantly affect the solution in the later time interval. The solution is plotted for all measured fragments of B. Clearly, the solution is poorly approximated in the first time interval, but the solution in the second interval remains accurately approximated and asymptotically approaches the true steady-state solution.

To demonstrate the important stability properties of the Radau IIA methods, we next solved the toy network using a larger than permissible step size in the stiff region of the dynamics (**Fig 5**). In this case, we divided the time domain into two contiguous intervals: [0, 10] and [10, 20] and performed the parameter estimation using the 5^th^-order method. As expected, we found that the solution was approximated poorly in the [0, 10] interval. This is indicated by the high measurement residuals in this region. However, the solution in the subsequent interval, [10, 20], was not significantly impacted by the poor approximation in the previous interval, as indicated by the small measurement residuals in this interval. Furthermore, the numerical approximation of the solution asymptotically approaches the true steady-state solution of the system despite the poor approximation in the first time interval. In this case, the steady-state solution is that all metabolites are fully enriched, which is consistent with the numerical approximation. This numerical stability is an important feature of methods, such as Radau IIA, which are suitable for stiff ODE systems.

When choosing time points to segregate the time domain into intervals, the user must be mindful of the dynamics of a typical isotope labeling experiment. In a typical experiment, the labeling states of upstream metabolites change rapidly immediately after the isotopically labeled compound is introduced to the cell culture. After this initial rapid transient, the dynamics become slower as the system relaxes toward a steady-state labeling distribution. A system whose dynamics operate in two (or more) different timescales is usually referred to as being stiff. Isotope labeling states frequently exhibit stiff dynamics with the stiff, rapidly changing region of the dynamics occurring at the start. Therefore, a user should assign time points that result in short time intervals at early times to accurately capture the ODE solution in the rapidly changing region. Wider time intervals may be assigned at later times because the solution is expected to be slowly varying.

We have found that the most efficient way to estimate fluxes for an inst-MFA model is to initially perform a set of restarts of the optimization from random initial feasible flux and pool size distributions (see **S5 Text** for the method used by eiFlux) using the 3^rd^-order Radau IIA collocation method. The best solution from these restarts should subsequently be used as the initial point for an optimization using the 9^th^-order Radau IIA method to improve the solution’s accuracy. eiFlux allows the user to specify this refinement such that it occurs automatically.

We suggest the following guidelines when choosing time points and implementing a collocation method:

- Time points should be assigned that result in short time intervals at early times to accurately capture the rapidly changing dynamics in this region, and longer time intervals at later times when the solution is slowly varying and segregating into too many time intervals would be computationally wasteful.
- To maximize computational efficiency, the optimization should be restarted multiple times from random initial parameter values using the 3rd-order Radau IIA collocation method to search for a global optimum. The best solution from these restarts serves as the starting point for an optimization using a higher order method, such as the 5th-order or 9th-order Radau IIA method (9th-order is preferred).
- If the user observes that the solution is approximated poorly when plotted, as would be indicated by an apparent discontinuity in the derivative between time intervals, then an additional time point should be added within the poorly approximated time interval to split this interval in two and obtain a better approximation.

### *E*. *coli* tandem inst-MFA example

We demonstrate eiFlux on a realistic example, that of *E*. *coli* central carbon metabolism. This network is based on that presented by Young et al. [9]. It contains a total of 58 metabolites having 5152 isotopomers (5094 cumomers) and 84 reactions having 26 independent fluxes. For a given flux distribution and a randomly assigned metabolite pool size distribution, we simulated transient tandem MS measurements from a hypothetical experiment in which *E*. *coli* is fed 99% uniformly ^13^C-labeled glucose. The simulated tandem MS data includes the metabolites and daughter fragments described by Rühl et al. [32]. In such an experiment, the steady-state isotope labeling distribution provides no information on metabolic pathway fluxes. Therefore, this information must be extracted from the transient (isotopically nonstationary) labeling data. In total, 2338 measurements were fit to the model. The acceptable SSR based on the 95% confidence interval was 2368. To fit this data type, we used the cumomer modeling formalism to construct the NLP.

We perturbed the simulated data with random normally-distributed error to simulate experimental error, then we fit this data to the model using eiFlux to estimate fluxes and pool sizes. Because the simulated dataset consisted of complete MIDs, measurement scaling factors were not used. To search for a best-fit solution, the optimization was restarted 15 times from random initial feasible flux and pool size distributions, using the 3^rd^-order Radau IIA collocation method to treat the ODEs. These starting points were far from the optimum, with each of these starting points having an initial objective function values on the order of 10^6^. All restarts converged to a narrow SSR range—2294 (3 times), 2293 (2 times), and 2288 (10 times). The fact that the optimizations tightly converged from very poor starting points is a testament to the robust convergence properties of the collocation NLP approach to inst-MFA, and demonstrates a distinct advantage over traditional methods that use the shooting method. The best solution from these 15 restarts was used as the initial point for an optimization using the 9^th^-order Radau IIA method to improve the accuracy of the solution, which achieved an SSR of 2212. The 15 restarts took a total time of 144.3 minutes, or an average of 9.62 minutes per restart (median: 8.78 minutes) on an Intel Core i7-7700 CPU @ 3.60 GHz running on a single processor core. The refinement step using the 9^th^-order method took 5.80 minutes.

**Fig 6:**
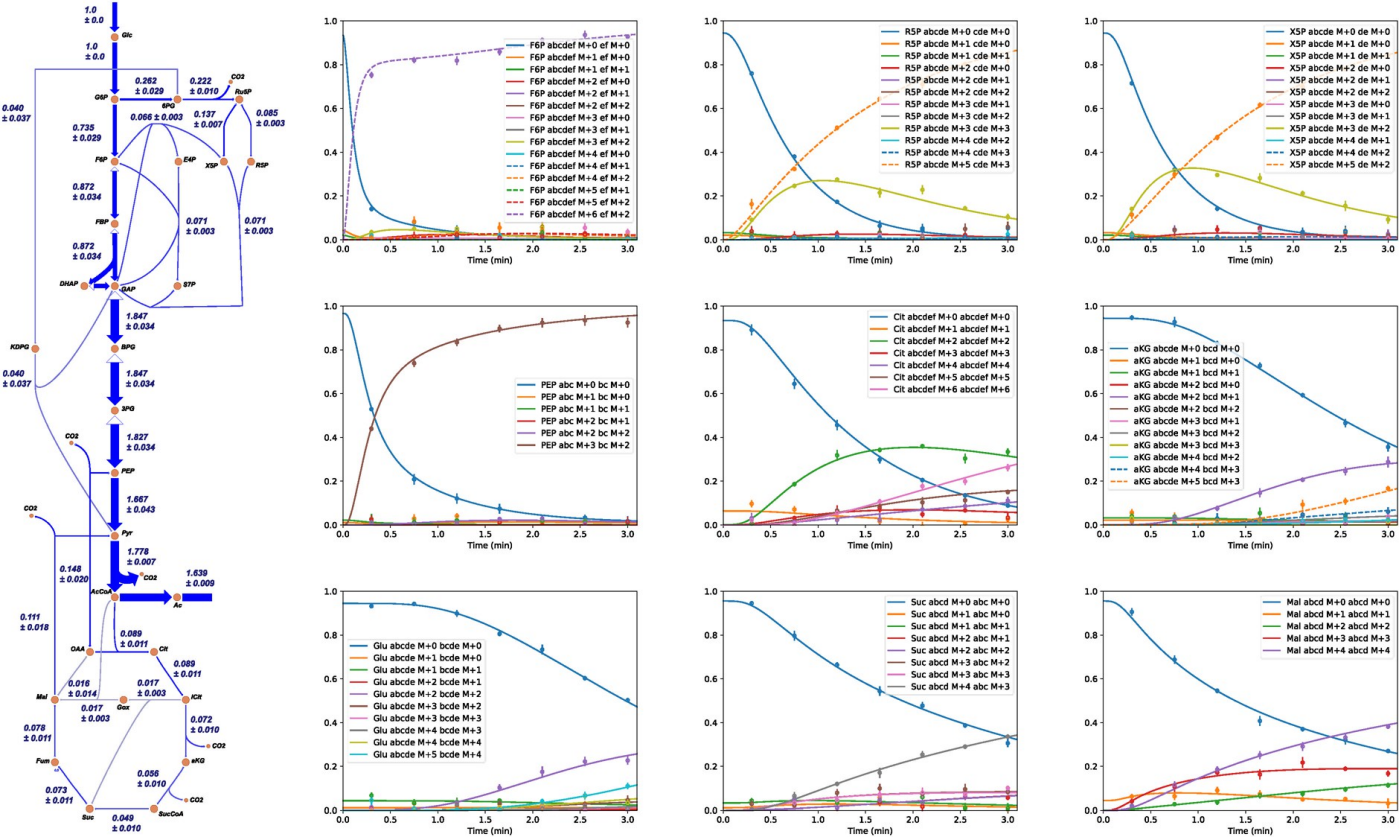
(Left) The central carbon metabolic reactions of the E. coli model, generated using Escher. Not shown are peripheral reactions that produce biomass metabolites (amino acids). For a full list of network reactions and their associated carbon atom rearrangements, see **S4 Text**. (Right) A set of representative model fits to the simulated tandem MS measurement data of several metabolites. For the full set of model fits to all simulated data, see **S4 Text**.

Ideally, we would recover the flux distribution from which the simulated data was generated. However, because the data was perturbed with artificial uncertainties, the original flux distribution may not be recovered exactly. Advantageously, the flux through fermentative acetate synthesis converged to nearly its actual value despite not being a measured flux. This demonstrates that using isotopically nonstationary tandem MS data can potentially allow one to accurately estimate some metabolite influxes and effluxes without directly measuring them. The flux map and model fit to some representative data is shown in **Fig 6**. To estimate flux confidence intervals, we used bootstrap Monte-Carlo [33]. This approach has been used previously for MFA studies; see [34] for an example and a brief explanation. A comparison between a subset of the actual flux values with the estimated flux values is displayed in **Table 2**. Clearly, eiFlux accurately estimates the fluxes through these important reactions, despite the measurements being perturbed by simulated measurement error.

**Table 2:** True and estimated flux values are compared for a subset of reactions of the *E*. *coli* central carbon metabolism network. The difference is calculated between the true and estimated fluxes. Clearly, eiFlux accurately estimates the fluxes from a simulated set of measurements perturbed by simulated measurement error. Abbreviations: pentose phosphate pathway (PPP), Entner-Doudoroff pathway (EDP), tricarboxylic acid (TCA), glucose 6-phosphate (G6P), fructose 6-phosphate (F6P), glyceraldehyde 3-phosphate (GAP), 1,3-bisphosphoglycerate (BPG), 6-phosphogluconate (6PG), 2-keto-3-deoxy-6-phosphogluconate (KDPG), oxaloacetate (OAA), acetyl-CoA (AcCoA), citrate (Cit), isocitrate (iCit), glyoxylate (Gox), succinate (Suc), malate (Mal), pyruvate (Pyr), phosphoenolpyruvate (PEP), acetate (Ac).

Pathway	Reaction	True	Estimated		Difference
			Mean	SD	
**glycolysis**	G6P → F6P	0.7367	0.7354	0.0295	0.0013
**glycolysis**	GAP → BPG	1.8510	1.8474	0.0343	0.0037
**PPP**	6PG → Ru5P + CO_2_	0.2255	0.2219	0.0100	0.0036
**EDP**	6PG → KDPG	0.0347	0.0396	0.0370	0.0049
**TCA cycle**	OAA + AcCoA → Cit	0.0961	0.0888	0.0112	0.0073
**glyoxylate shunt**	iCit → Gox + Suc	0.0195	0.0166	0.0027	0.0028
**cataplerosis**	Mal → Pyr + CO_2_	0.1377	0.1111	0.0176	0.0266
**anaplerosis**	PEP + CO_2_ → OAA	0.1612	0.1480	0.0196	0.0132
**efflux**	Ac →	1.6254	1.6391	0.0088	0.0138

Upon running eiFlux, a JSON file containing the network reactions is automatically generated. This JSON file is readable by Escher, an application used for visualizing metabolic networks and flux distributions [35]. This allows the user to visualize their network model and evaluate fluxes quickly and easily. This fast visualization can aid in model development and analysis. The flux map for the *E*. *coli* network in **Fig 6** was generated using Escher. eiFlux also automatically plots the model fits to the data for visual evaluation, with some representative model fits to the *E*. *coli* tandem MS example data shown in **Fig 6**. The complete set of model fits is shown in **S4 Text**. **S1 Data** contains the simulated data fit to this model.

### Comparison with existing software

To directly compare the NLP approach with the shooting method, we solved the same model using both eiFlux and the MFA software INCA (version 2.0) [24], which uses the shooting method. We solved the *E*. *coli* model described previously using a synthetic single-MS time-series dataset (**S1 Data**). To develop this synthetic dataset, we generated the simulated measurements for the set of GC-MS fragments listed in [36] using the same flux and pool size distribution as was used when generating the simulated tandem-MS data. This data was generated for a hypothetical experiment feeding 80% 1,2-^13^C and 20% uniformly ^13^C-labeled glucose. We then perturbed these measurements with simulated measurement error. To enable a direct comparison with INCA, measurement scaling factors were used in eiFlux in this example. We restarted the optimization 20 times from random initial feasible flux and pool size distributions using each software. These initial flux and pool size distributions were generated in INCA by setting the “number of logs to perturb initial values” option to either 4 or 5 for a given restart, ensuring that the starting points were far from the optimum. These values were transferred into eiFlux to serve as the starting point for the optimization. This guaranteed that the initial feasible flux and pool size distributions were the same in both INCA and eiFlux for each of these restarts, ensuring a fair comparison. With eiFlux, these 20 restarts were each performed using the 3^rd^-order Radau IIA method followed by a refinement using the 9^th^-order Radau IIA method to improve the accuracy of the solution. To compare the two approaches, we report the average convergence time per restart and the fraction of restarts that converged within a small neighborhood of the best-computed optimum by each platform. In this example, both eiFlux and INCA were run on a personal computer using an Intel Core i5-8250U CPU @1.60 GHz processor running on a single processor core.

**Fig 7:**
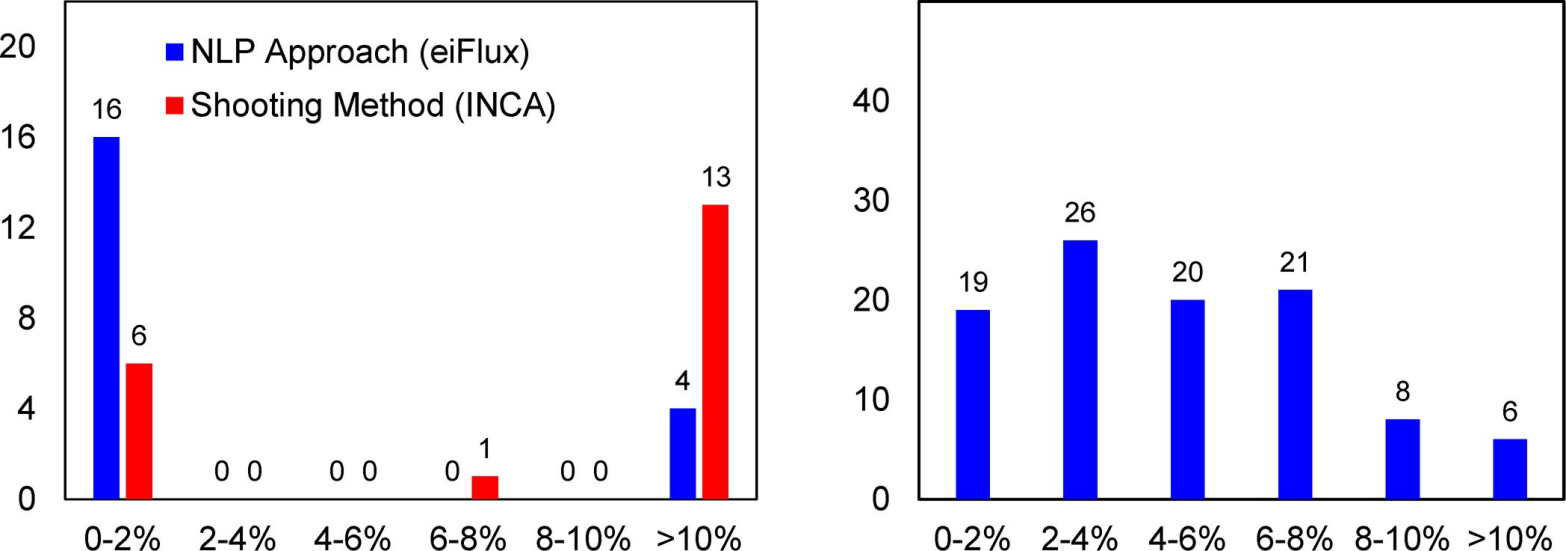
(Left) Number of restarts out of 20 that converged within a vicinity (percent) of the best-computed optimum for the NLP approach and the shooting method. For the NLP approach, these 20 restarts were performed with the 3rd-order Radau IIA method followed by refinement using the 9th-order method. The best achieved optimum was 660.39 with the 3rd-order method and 644.14 after the 9th-order refinement. For the shooting method we used the software INCA, and the best achieved optimum was 646.34. (Right) Results from 100 restarts for the genome-scale Synechocystis example. Using the NLP approach, most optimizations started from a random initial point converge to within 10% of the best-computed optimal solution, and a large fraction within 2%. This highlights the robustness of the NLP approach for inst-MFA applications.

Using eiFlux, the best computed optimal solution from the 20 restarts using the 3^rd^-order Radau IIA method was 660.39, which reduced to 644.14 after refinement using the 9^th^-order method. Of these 20 restarts, 16 converged within 2% of the best computed optimum, defined as those that converged with an SSR in the range 660.39 – 673.60 using the 3^rd^-order Radau IIA method. The remaining 4 restarts converged to a poor solution greater than 10% above this optimum. The optimizations using the 3^rd^-order Radau IIA method required an average time of 2.55 min per restart. The 9^th^-order refinements took an average of 2.65 min to converge. Using INCA, the best computed optimal solution from the 20 restarts was 646.34. Of these 20 restarts, 6 converged within 2% of the best computed optimum (those with an optimal SSR in the range 646.34 – 659.27). A total of 13 converged to a poor solution greater than 10% above this optimum. These results are summarized in **Fig 7**. The optimizations using INCA required an average time of 7.57 min per restart. This comparison demonstrates that for core networks, the NLP approach has a comparable run time to the shooting method, but it offers significantly more robust convergence to the optimal solution.

### Genome-scale *Synechocystis* inst-MFA Example

To demonstrate the scalability of this approach, we solved the *Synechocystis* PCC 6803 genome-scale inst-MFA model presented by Gopalakrishnan et al. [37]. This model was fit to the dataset presented by Young et al. [38]. In the analysis by Gopalakrishnan et al. [37], the authors performed inst-MFA using the shooting method to estimate fluxes and pool sizes in a genome-scale model of *Synechocystis* PCC 6803 (hereafter, *Synechocystis*). In this approach, the authors first performed FVA to eliminate reactions from the model that could not carry flux under the studied photoautotrophic growth conditions, which resulted in a reduction in the model size. However, the model remained quite large and required the simulation of 8.4×10^5^ ODEs at each iteration of a Levenberg-Marquardt optimization algorithm. This number of equations included both the state equations needed to simulate the EMU dynamics and their sensitivities to the fitted parameters.

When using this approach, both the state and sensitivity equations must be solved from scratch at each iteration of the optimization algorithm. In the Levenberg-Marquardt algorithm, the sensitivity equations are used to determine a good step direction during each iteration for parameter estimation. In the inst-MFA case, both the state and the sensitivity equations are ODEs. The number of sensitivity equations scales as *n*×*p* in which *n* is the number of EMU mass fractions and *p* is the number of free parameters [23]. This scaling means that the number of sensitivity equations grows extremely large as the size of the network increases. The authors report 851 EMUs, having 2311 EMU mass fractions, each contributing a balance. There are 367 fitted parameters. The number of sensitivity equations is calculated by multiplying these two values, giving the reported 8.4×10^5^ ODEs that must be solved from scratch at each iteration of the Levenberg-Marquardt algorithm. This is the reason for the poor scalability of the shooting method approach.

While the authors did not report the computational time and memory required to solve this model, it is presumably quite large considering the size of the ODE system. The authors found that many predicted fluxes differed considerably between the core model and the genome-scale model. This indicates that modeling assumptions, such as pathway lumping, made in developing a core model can notably bias flux estimates. These results demonstrate that larger, more detailed models are preferable for accurately elucidating fluxes when using inst-MFA. This highlights the need for scalable approaches to MFA if such analyses with large-scale models are to become routine.

We repeated the flux and pool size estimation with the genome-scale *Synechocystis* model using eiFlux implementing the EMU modeling method. Because the MIDs being fit were incomplete and uncorrected, scaling factors were active as free parameters in the optimization. The natural isotope abundances of the additional atoms were accounted for by factoring their effects into the measurement mapping matrix, *M*_*nj*_, in the NLP. This allowed us to fit the data presented in this study. We restarted the optimization from 100 random initial feasible flux and pool size distributions using the 3^rd^-order Radau IIA method. The average convergence time was 15.89 min per restart. The short convergence time of this genome-scale model demonstrates that the NLP approach scales well to large, detailed networks which represent the current frontier of inst-MFA applications. Of these 100 restarts, 94 converged within 10%, and 19 converged within 2% of the best-computed optimum (568.23) when running the 3^rd^-order Radau IIA method. This demonstrates that the optimization does not frequently converge to poor local optima far from the globally optimal solution, and that this approach remains robust and efficient even for models of genome scale. These results are summarized in **Fig 7**. To improve the accuracy of the solution, the best solution from these 100 restarts was used as the initial point for an optimization using the 9^th^-order Radau IIA method. This 9^th^-order refinement converged to a solution with an optimal SSR of 513.30.

For the optimizations using the 3^rd^-order Radau IIA method, the NLP included ~80,000 algebraic equality constraints and variables after CONOPT’s model preprocessing step. For the 9^th^-order method, the NLP included ~182,000 algebraic equality constraints and variables. This is a significant simplification of the problem when compared to the 8.4×10^5^ ODEs that must be solved from scratch at each iteration of the Levenberg-Marquardt algorithm in the shooting method approach. This simplification results because the sensitivity equations do not need to be constructed and solved in the NLP approach.

The optimization was also memory-efficient, using less than 1 gigabyte of memory even for the 9^th^-order refinement step. Most of the memory required by CONOPT is used for storing the Jacobian of the constraints and its LU decomposition. While a large model such as this may have on the order of 10^5^ equality constraints and variables, the Jacobian is highly sparse. When running the refinement with the 9^th^-order Radau IIA method, the Jacobian had only 9.7×10^5^ nonzero elements after CONOPT’s model preprocessing step. GAMS and CONOPT use sparse linear algebra routines to accelerate computations and remain memory-efficient. Notably, second derivative information is only computed for the nonbasic variables in the form of a reduced Hessian. This reduced Hessian is small and has a negligible contribution to the memory usage of the CONOPT algorithm compared to the Jacobian and its LU decomposition. This example demonstrates that large-scale models can be solved efficiently on a standard personal computer using a single processor core.

## Discussion

MFA, especially inst-MFA, is a computationally intensive parameter estimation problem involving *O*(10-100) free parameters (fluxes and metabolite pool sizes) in a model that contains *O*(1000) ODEs and *O*(100-1000) measurements. Currently available algorithms developed for inst-MFA have used sequential optimization algorithms, wherein the DLS is recalculated *explicitly* and from scratch at each iteration of the optimization. This shooting method could potentially be inefficient and may not scale well to large networks. It is also known to suffer from small regions of convergence [13], often unable to robustly converge to global optima without a good initial guess. In this manuscript, we follow a different approach and formulate the MFA problem as an equality-constrained nonlinear program (NLP). In this approach, the DLS is not solved from scratch at each iteration, but is updated to satisfy the equality constraints. In other words, the DLS is handled *implicitly* without distinguishing the state variables from the model parameters. This method significantly increases the efficiency of the optimization, especially when the DLS consists of O(100-1000) labeling transients. It also scales well when the inst-MFA model contains a large number of state variables and parameters. The NLP approach enables either the cumomer or EMU framework to be used effectively and efficiently, leading to robust convergence and fast run times.

We formulated the MFA problem as an equality-constrained NLP by using the framework presented by Wiechert et al. [1]. This NLP may be solved using optimization algorithms implemented in state-of-the-art AMLs such as GAMS. To solve the inst-MFA parameter estimation problem, we used collocation to transcribe the ODE system into a set of algebraic constraints for the NLP. We found that the large-scale reduced gradient algorithm, CONOPT, implemented in GAMS, was particularly fast and robust at solving both the steady-state and inst-MFA problems. However, in principle, any general NLP solver may be used. For the inst-MFA problem, this approach scales well to large networks with many free parameters and has more robust convergence properties compared to traditional methods that use the shooting method. Furthermore, because the MFA problem may be formulated using the cumomer framework, tandem MS data is easily supported for both steady-state and inst-MFA. We assembled a software package, eiFlux, built using Python and GAMS that employs the NLP formulation and supports both steady-state and inst-MFA using either the cumomer or EMU framework.

After demonstrating proof of concept with a toy network, we used our formulation and eiFlux on a realistic *E*. *coli* tandem MS example. Here, we demonstrated that our NLP formulation converges robustly to a narrow SSR range for every restart performed from a poor and random initial point. Computation speeds were fast, and convergence to an optimum took less than 10 minutes on average for inst-MFA using the cumomer framework. In this example, the number of constraints and variables in the underlying inst-MFA NLP, which included the transcribed ODE system, were each ~180,000 after CONOPT’s model preprocessing step.

Next, we provided a direct comparison between eiFlux, which uses the NLP formulation, and INCA, which uses the shooting method on the core *E*. *coli* network fitting simulated single-MS data. We found that computation speeds were comparable for both platforms, but that eiFlux converged more robustly to the optimum as evidenced by the fraction of restarts from a random initial guess that converged within a small neighborhood of the best-computed optimum. Finally, we used eiFlux to solve a large genome-scale inst-MFA model of *Synechocystis* metabolism presented by Gopalakrishnan et al. [37] fitting time-series measurements presented by Young et al. [38]. This example demonstrated that the NLP formulation scales well to very large networks, and that using this formulation, such networks may be solved efficiently on a personal computer. The scalability of this approach makes the routine solution of large-scale inst-MFA models possible and significantly expands the scope in which inst-MFA may be applied in the future. We expect that this approach will allow large-scale models of complex eukaryotic networks (e.g. algae) and co-culture metabolism to be handled efficiently by inst-MFA.

In the past, increasing the speed and efficiency of the parameter estimation in steady-state and inst-MFA has emphasized solving for the labeling state quickly at each iteration of an optimization algorithm. Here, we focused on formulating the problem in an improved optimization framework, which allows for efficient scalability of the MFA problem to larger and more detailed networks. We found that this approach is particularly useful for inst-MFA, efficiently solving a genome-scale model. Finally, the flexibility of the NLP approach allows for incorporating additional linear and nonlinear constraints on fluxes and pool sizes that are not easily incorporated in current MFA or inst-MFA applications. These types of constraints may be incorporated for future extensions and uses of MFA and represents a possible future direction of this work.

## Supporting information

S1 Text Example of a reduced gradient nonlinear programming algorithm. (PDF)

S2 Text Mathematical details of handling of equality constraints by CONOPT. (PDF)

S3 Text Simple isotopomer network to illustrate collocation. (PDF)

S4 Text Toy network and *E*. *coli* tandem mass spectrometry inst-MFA examples. (PDF)

S5 Text Internal method of generating initial feasible flux and pool size distributions in eiFlux. (PDF)

S6 Text List of symbols. (PDF)

S1 Fig Isotopomer information measured by tandem MS. (PDF)

S1 Data Simulated data used for toy network and *E*. *coli* examples. (XLSX)

S1 File Zip file containing the following: eiFlux models for all examples presented in this article. The text files in these folders are used as inputs to eiFlux. The text file modelfile.txt is used to specify which model to run. The GAMS scripts used by eiFlux, in the folder GAMS_Model. A demo version of eiFlux that specifically runs two models listed above, thus fully reproducing the results in this manuscript. This is a compiled Python file, eiFlux_Limited.pyc. This demo version only runs only the two models listed above. Users may change model parameters such as the time nodes, but not the models themselves. After following the instructions given in the Installation_Instructions.pdf file, users may run the eiFlux_Limited.pyc file to run the software.
(ZIP)

## References

[1] Wiechert W, Möllney M, Isermann N, Wurzel M, de Graaf AA. Bidirectional reaction steps in metabolic networks: III. Explicit solution and analysis of isotopomer labeling systems. Biotechnol Bioeng. 1999;66: 69–85. doi: 10.1002/(SICI)1097-0290(1999)66:2&lt;69::AID-BIT1&gt;3.0.CO;2-6 10567066

[2] Antoniewicz MR. A guide to metabolic flux analysis in metabolic engineering: Methods, tools and applications. Metab Eng. 2021;63: 2–12. doi: 10.1016/j.ymben.2020.11.002 33157225

[3] Cheah YE, Young JD. Isotopically nonstationary metabolic flux analysis (INST-MFA): putting theory into practice. Curr Opin Biotechnol. 2018;54: 80–87. doi: 10.1016/j.copbio.2018.02.013 29522915

[4] Wiechert W, Nöh K. Isotopically non-stationary metabolic flux analysis: complex yet highly informative. Curr Opin Biotechnol. 2013;24: 979–986. doi: 10.1016/j.copbio.2013.03.024 23623747

[5] Antoniewicz MR, Kelleher JK, Stephanopoulos G. Elementary metabolite units (EMU): A novel framework for modeling isotopic distributions. Metab Eng. 2007;9: 68–86. doi: 10.1016/j.ymben.2006.09.001 17088092 PMC1994654

[6] Sriram G, Shanks JV. Improvements in metabolic flux analysis using carbon bond labeling experiments: bondomer balancing and Boolean function mapping. Metab Eng. 2004;6: 116–132. doi: 10.1016/j.ymben.2004.02.003 15113565

[7] van Winden WA, Heijnen JJ, Verheijen PJT. Cumulative bondomers: A new concept in flux analysis from 2D [^13^C, ^1^H] COSY NMR data. Biotechnol Bioeng. 2002;80: 731–745. doi: 10.1002/bit.10429 12402319

[8] Srour O, Young JD, Eldar YC. Fluxomers: a new approach for ^13^C metabolic flux analysis. BMC Syst Biol. 2011;5: 1–14. doi: 10.1186/1752-0509-5-1 21846358 PMC3750106

[9] Young JD, Walther JL, Antoniewicz MR, Yoo H, Stephanopoulos G. An elementary metabolite unit (EMU) based method of isotopically nonstationary flux analysis. Biotechnol Bioeng. 2008;99: 686–699. doi: 10.1002/bit.21632 17787013

[10] Shin S, Venturelli OS, Zavala VM. Scalable nonlinear programming framework for parameter estimation in dynamic biological system models. PLOS Comput Biol. 2019;15: e1006828. doi: 10.1371/journal.pcbi.1006828 30908479 PMC6467427

[11] Kirkpatrick S, Gelatt CD, Vecchi MP. Optimization by Simulated Annealing. Science. 1983;220: 671–680. doi: 10.1126/science.220.4598.671 17813860

[12] Rodriguez-Fernandez M, Mendes P, Banga JR. A hybrid approach for efficient and robust parameter estimation in biochemical pathways. Biosystems. 2006;83: 248–265. doi: 10.1016/j.biosystems.2005.06.016 16236429

[13] Peifer M, Timmer J. Parameter estimation in ordinary differential equations for biochemical processes using the method of multiple shooting. IET Syst Biol. 2007;1: 78–88. doi: 10.1049/iet-syb:20060067 17441551

[14] Betts JT. Practical Methods for Optimal Control and Estimation Using Nonlinear Programming, 2nd ed. Society for Industrial and Applied Mathematics; 2010.

[15] Drud A. CONOPT: A GRG code for large sparse dynamic nonlinear optimization problems. Math Program. 1985;31: 153–191. doi: 10/bhwtmp

[16] Drud A. CONOPT—A Large-Scale GRG Code. ORSA J Comput. 1994;6: 207–216. doi: 10/cww2m7

[17] General Algebraic Modeling System (GAMS). 2751 Prosperity Ave, Suite 210, Fairfax VA 22031: GAMS Development Corporation. Fairfax VA, USA; 2021.

[18] Choi J, Antoniewicz MR. Tandem mass spectrometry: A novel approach for metabolic flux analysis. Metab Eng. 2011;13: 225–233. doi: 10.1016/j.ymben.2010.11.006 21134484

[19] Möllney M, Wiechert W, Kownatzki D, de Graaf AA. Bidirectional reaction steps in metabolic networks: IV. Optimal design of isotopomer labeling experiments. Biotechnol Bioeng. 1999;66: 86–103. doi: 10.1002/(sici)1097-0290(1999)66:2&lt;86::aid-bit2&gt;3.0.co;2-a 10567067

[20] Abadie J, Carpentier J. Optimization. Academic Press; 1969.

[21] Nöh K, Wahl A, Wiechert W. Computational tools for isotopically instationary ^13^C labeling experiments under metabolic steady state conditions. Metab Eng. 2006;8: 554–577. doi: 10.1016/j.ymben.2006.05.006 16890470

[22] Wiechert W, Nöh K. From stationary to instationary metabolic flux analysis. Adv Biochem Eng Biotechnol. 2005;92: 145–172. doi: 10.1007/b98921 15791936

[23] Mottelet S, Gaullier G, Sadaka G. Metabolic Flux Analysis in Isotope Labeling Experiments Using the Adjoint Approach. IEEE/ACM Trans Comput Biol Bioinform. 2017;14: 491–497. doi: 10.1109/TCBB.2016.2544299 28113867

[24] Young JD. INCA: a computational platform for isotopically non-stationary metabolic flux analysis. Bioinformatics. 2014;30: 1333–1335. doi: 10.1093/bioinformatics/btu015 24413674 PMC3998137

[25] Kajihata S, Furusawa C, Matsuda F, Shimizu H. OpenMebius: An Open Source Software for Isotopically Nonstationary ^13^C-Based Metabolic Flux Analysis. BioMed Res Int. 2014;2014: e627014. doi: 10.1155/2014/627014 25006579 PMC4071984

[26] Fröhlich F, Kaltenbacher B, Theis FJ, Hasenauer J. Scalable Parameter Estimation for Genome-Scale Biochemical Reaction Networks. PLOS Comput Biol. 2017;13: e1005331. doi: 10.1371/journal.pcbi.1005331 28114351 PMC5256869

[27] Tjoa IB, Biegler LT. Simultaneous solution and optimization strategies for parameter estimation of differential-algebraic equation systems. Ind Eng Chem Res. 1991;30: 376–385. doi: 10/dzk9pt

[28] Hairer E, Wanner G. Stiff differential equations solved by Radau methods. J Comput Appl Math. 1999;111: 93–111. doi: 10/b3jgts

[29] Huynh HT. Collocation and Galerkin Time-Stepping Methods. 19th AIAA Computational Fluid Dynamics. San Antonio, Texas: American Institute of Aeronautics and Astronautics; 2009. doi: 10/gg4rxz

[30] Antoniewicz MR. Tandem mass spectrometry for measuring stable-isotope labeling. Curr Opin Biotechnol. 2013;24: 48–53. doi: 10.1016/j.copbio.2012.10.011 23142542

[31] Choi J, Grossbach MT, Antoniewicz MR. Measuring Complete Isotopomer Distribution of Aspartate Using Gas Chromatography/Tandem Mass Spectrometry. Anal Chem. 2012;84: 4628–4632. doi: 10.1021/ac300611n 22510303

[32] Rühl M, Rupp B, Nöh K, Wiechert W, Sauer U, Zamboni N. Collisional fragmentation of central carbon metabolites in LC-MS/MS increases precision of ^13^C metabolic flux analysis. Biotechnol Bioeng. 2012;109: 763–771. doi: 10.1002/bit.24344 22012626

[33] Press WH, Teukolsky SA, Vetterling WT, Flannery BP. Numerical Recipes 3rd Edition: The Art of Scientific Computing. 3rd ed. Cambridge University Press; 2007.

[34] Nargund S, Misra A, Zhang X, D. Coleman G, Sriram G. Flux and reflux: metabolite reflux in plant suspension cells and its implications for isotope-assisted metabolic flux analysis. Mol Biosyst. 2014;10: 1496–1508. doi: 10.1039/c3mb70348g 24675729

[35] King ZA, Dräger A, Ebrahim A, Sonnenschein N, Lewis NE, Palsson BO. Escher: a web application for building, sharing, and embedding data-rich visualizations of biological pathways. PLOS Comput Biol. 2015;11: e1004321. doi: 10.1371/journal.pcbi.1004321 26313928 PMC4552468

[36] Young JD, Allen DK, Morgan JA. Isotopomer Measurement Techniques in Metabolic Flux Analysis II: Mass Spectrometry. Plant Metabolism: Methods and Protocols. New York: Springer Science+Business Media, LLC; 2014. 10.1007/978-1-62703-661-0_7 24218212

[37] Gopalakrishnan S, Pakrasi HB, Maranas CD. Elucidation of photoautotrophic carbon flux topology in Synechocystis PCC 6803 using genome-scale carbon mapping models. Metab Eng. 2018;47: 190–199. doi: 10.1016/j.ymben.2018.03.008 29526818

[38] Young JD, Shastri AA, Stephanopoulos G, Morgan JA. Mapping photoautotrophic metabolism with isotopically nonstationary 13C flux analysis. Metab Eng. 2011;13: 656–665. doi: 10.1016/j.ymben.2011.08.002 21907300 PMC3210925

