# Supplementary material for: Isotope-assisted metabolic flux analysis as an equality-constrained nonlinear program for improved scalability and robustness
Source: PLoS Comput Biol. 2022 Mar 24;18(3):e1009831. doi: 10.1371/journal.pcbi.1009831 (PMC8947808; doi:10.1371/journal.pcbi.1009831)
Supplement: S1 Text — (PDF) [file pcbi.1009831.s001.pdf]

Example of a Reduced Gradient Nonlinear Programming Algorithm

A general equality-constrained nonlinear programming problem is shown in (S1.1). Here, $f(\mathbf{x})$ is the objective function to be minimized, which may be nonlinear. The vector function $g(\mathbf{x})$ is the system of equality constraints, which may in general be nonlinear. The variables, $\mathbf{x}$, may additionally be bounded by lower and upper bounds, lb and ub, respectively.

$$\begin{aligned} \text{minimize} \quad & f(\mathbf{x}) \\ \text{s.t.} \quad & g(\mathbf{x}) = \mathbf{0} \\ & lb \le \mathbf{x} \le ub \end{aligned} \tag{S1.1}$$

The Lagrangian (S1.2) may be formed from the model components introduced in (S1.1).

$$L(\mathbf{x}, \lambda) = f(\mathbf{x}) - \lambda^{\mathsf{T}} g(\mathbf{x}) \tag{S1.2}$$

In equation (S1.2), $L(\mathbf{x}, \lambda)$ is the Lagrangian, and λ is the vector of Lagrange multipliers corresponding to the equality constraints, $g(\mathbf{x})$. Each $g_i(\mathbf{x})$ has an associated Lagrange multiplier λ_i. Optimizing an equality-constrained program involves finding stationary points of the Lagrangian (S1.3). A stationary point is identified as one in which $\nabla L(\mathbf{x}, \lambda) = \mathbf{0}$.

$$\nabla L(\mathbf{x}, \lambda) = \nabla f(\mathbf{x}) - \left[\nabla g(\mathbf{x})\right]^{\mathsf{T}} \lambda = \mathbf{0} \tag{S1.3}$$

In (S1.3), $\nabla L(\mathbf{x}, \lambda)$ is the gradient of the Lagrangian with respect to the model variables, $\mathbf{x}$. This gradient may be zero at a maximum, minimum, or a saddle point of the Lagrangian. The term $\nabla g(\mathbf{x})$ is a matrix whose rows consist of the gradients of the constraints (S1.4), and is also known as the Jacobian of the constraints, $J(\mathbf{x})$.

$$\nabla g(\mathbf{x}) = \begin{pmatrix} -\nabla g_1(\mathbf{x})^{\mathsf{T}} - \\ -\nabla g_2(\mathbf{x})^{\mathsf{T}} - \\ \vdots \\ -\nabla g_m(\mathbf{x})^{\mathsf{T}} - \end{pmatrix} = J(\mathbf{x}) \tag{S1.4}$$

With the notational substitution of (S1.4), (S1.3) becomes (S1.5).

$$\nabla L(\mathbf{x}, \lambda) = \nabla f(\mathbf{x}) - J(\mathbf{x})^{\mathsf{T}} \lambda = \mathbf{0} \tag{S1.5}$$

The form shown in (S1.5) is used in constructing the reduced gradient algorithm.

The generalized reduced gradient (GRG) algorithm works well for nonlinear optimization problems in which the objective function and constraints are smooth and differentiable [1,2]. It is particularly well-suited for optimization problems in which the total number of equality constraints is similar to the total number of model variables. This is because the GRG algorithm involves partitioning the variables into sets. One set consists of basic variables, $\mathbf{x}_b$, and the other of nonbasic variables, $\mathbf{x}_{nb}$. At each iteration of the GRG algorithm, the nonbasic variables are propagated to a better point (one that improves the objective function), and the basic variables are propagated to the new point such that they satisfy the equality constraints [1,3]. The following list of steps in a general GRG algorithm was adapted from Drud [3]:

1. Find a feasible starting point, $\mathbf{x}_0$. At this point, compute the objective function $f(\mathbf{x}_0)$, gradient of the objective function $\nabla f(\mathbf{x}_0)$, and Jacobian of the constraints $J(\mathbf{x}_0)$.
2. Select a set of n basic variables, $\mathbf{x}_b$, such that J_b, the submatrix of the basic columns of J, is nonsingular.

3. Solve for the multipliers $J_b^T \lambda = \nabla_b f$.
4. Compute the reduced gradient $\mathbf{r} = \nabla f - J^\mathsf{T} \lambda$.
5. Terminate if a stopping criterion is met.
6. Find a search direction, $\mathbf{d}$, for the nonbasic variables based on $\mathbf{r}$ and compute the tangent direction.
7. Perform a search along direction $\mathbf{d}$. For each step, adjust $\mathbf{x}_b$ to satisfy $g(\mathbf{x}_b, \mathbf{x}_{nb}) = \mathbf{0}$ using a pseudo-Newton process.
8. Repeat these steps with the current point.

This process is next illustrated in a simple example. This example GRG algorithm is simply used for illustration purposes. Large-scale GRG algorithms, such as CONOPT [2,3], are far more sophisticated, making them much more effective and efficient. Therefore, the following example is illustrative only, and is not representative of the CONOPT implementation.

The example equality-constrained nonlinear program is shown in (S1.6).

$$\begin{aligned} \text{minimize} \quad & f(\mathbf{x}) = 200 \;\; x_1^2 + x_2^2 + x_1 x_2 \;\; - 360\, x_1 - 240\, x_2 + 168 \\ \text{s.t.} \quad & g_1(\mathbf{x}) = {x_1}^2 + {x_3}^2 - x_1 - x_2 + 0.5 = 0 \\ & g_2(\mathbf{x}) = x_1 x_2 + x_1 + x_4 - 1 = 0 \\ & g_3(\mathbf{x}) = x_5 - x_2 - x_1 = 0 \\ & \mathbf{x} \geq 0 \end{aligned} \qquad \text{(S1.6)}$$

This example was chosen because it consists of a nonlinear objective function with nonlinear constraints. Specifically, $g_3(\mathbf{x})$ is linear, while $g_1(\mathbf{x})$ and $g_2(\mathbf{x})$ are both nonlinear. Also note that (S1.6) is in the general format shown in (S1.1). Here, the objective function is only a function of two of the model variables, x_1 and x_2. Problems with this structure are very common in least-squares model fitting. This problem has a total of 5 variables and 3 equality constraints.

Operating the GRG algorithm requires repeated use of the gradient of the objective function and the Jacobian of the constraints. They are listed here for the example problem for reference:

$$\nabla f(\mathbf{x}) = \begin{pmatrix} 400\, x_1 + 200\, x_2 - 360 \\ 400\, x_2 + 200\, x_1 - 240 \\ 0 \\ 0 \\ 0 \end{pmatrix}, \; J(\mathbf{x}) = \begin{pmatrix} 2x_1 - 1 & -1 & 2x_3 & 0 & 0 \\ x_2 + 1 & x_1 & 0 & 1 & 0 \\ -1 & -1 & 0 & 0 & 1 \end{pmatrix} \qquad \text{(S1.7)}$$

Step 1: Initialize with a feasible starting point

Start with a point that satisfies the constraints $g(\mathbf{x}) = \mathbf{0}$. Calculate the objective function, gradient of the objective function, and Jacobian at this point. We will begin with the arbitrarily chosen feasible point $\mathbf{x}_0 = \begin{bmatrix} 0 & 0.5 & 0 & 1 & 0.5 \end{bmatrix}^\mathsf{T}$. This starting point is chosen simply because it satisfies the constraints. The objective function $f(\mathbf{x}_0)$, objective function gradient $\nabla f(\mathbf{x}_0)$, and Jacobian $J(\mathbf{x}_0) = J_0$ at this point are shown in (S1.8). The subscript, 0, is the index of the current iteration.

$$f(\mathbf{x}_0) = 98.0, \quad \nabla f(\mathbf{x}_0) = \begin{pmatrix} -260 \\ -40 \\ 0 \\ 0 \\ 0 \end{pmatrix}, \quad J_0 = \begin{pmatrix} -1 & -1 & 0 & 0 & 0 \\ 1.5 & 0 & 0 & 1 & 0 \\ -1 & -1 & 0 & 0 & 1 \end{pmatrix} \qquad \text{(S1.8)}$$

Step 2: Select a set of basic variables

To simplify the notation, the subscripted iteration index is omitted from J for the remaining steps. The best set of basic variables is usually not obvious and is determined by the solver, however it is generally wise to choose a set of basic variables such that the submatrix, J_b, of the Jacobian corresponding to the basic variables is well-conditioned. This is because J_b must be invertible [1]. The remaining nonbasic columns of J are stored in matrix J_{nb}. The variable vector, $\mathbf{x}$, is partitioned accordingly. It is important to note that for a given choice of basic variables, J_b may be well-conditioned at one point, but ill-conditioned at another point. Therefore, the best set of basic variables may change from one iteration to the next. In this example, x_3 would be a poor choice for a basic variable in the first iteration. This is because the column of the Jacobian corresponding to x_3 is all zeros at the initial point. Choosing x_2, x_4, and x_5 to be basic, the vectors $\mathbf{x}_b$ and $\mathbf{x}_{nb}$, and matrices J_b and J_{nb} are shown in (S1.9) for the first iteration.

$$\mathbf{x}_b = \begin{pmatrix} x_2 \\ x_4 \\ x_5 \end{pmatrix}, \quad \mathbf{x}_{nb} = \begin{pmatrix} x_1 \\ x_3 \end{pmatrix}, \quad J_b = \begin{pmatrix} -1 & 0 & 0 \\ 0 & 1 & 0 \\ -1 & 0 & 1 \end{pmatrix}, \quad J_{nb} = \begin{pmatrix} -1 & 0 \\ 1.5 & 0 \\ -1 & 0 \end{pmatrix} \tag{S1.9}$$

Step 3: Solve for the Lagrange multipliers

Candidate optima are found as the stationary points of the Lagrangian, defined in (S1.2). These stationary points, $\mathbf{x}^*, \lambda^*$, will satisfy $\nabla L(\mathbf{x}^*, \lambda^*) = \mathbf{0}$. Here we seek points that satisfy (S1.10).

$$\nabla L(\mathbf{x}, \lambda) = \nabla f(\mathbf{x}) - J(\mathbf{x})^{\mathsf{T}} \lambda = \mathbf{0} \tag{S1.10}$$

Considering only the basic variables, (S1.10) becomes (S1.11), which is an exactly determined system for the multipliers, λ.

$$\nabla f_b - J_b^{\mathsf{T}} \lambda = \mathbf{0} \tag{S1.11}$$

Solving (S1.11) for λ gives (S1.12) for the first iteration.

$$\lambda = {J_b^T}^{-1} \nabla f_b = \begin{pmatrix} -1 & 0 & -1 \\ 0 & 1 & 0 \\ 0 & 0 & 1 \end{pmatrix}^{-1} \begin{pmatrix} -40 \\ 0 \\ 0 \end{pmatrix} = \begin{pmatrix} 40 \\ 0 \\ 0 \end{pmatrix} \tag{S1.12}$$

In solvers using the GRG method, J_b^T is typically not inverted at each iteration of the algorithm, as this would be computationally inefficient. Instead, the inverse is computed at the first iteration and updated at each subsequent iteration using a suitable approximation [1].

Step 4: Compute the reduced gradient

Next, the reduced gradient, $\mathbf{r}$, is computed using (S1.13). The reduced gradient is calculated as the gradient of the Lagrangian using the values of the multipliers calculated in (S1.12).

$$\mathbf{r} = \nabla f - J^{\mathsf{T}} \lambda \tag{S1.13}$$

Only the nonbasic variables may have nonzero values in the reduced gradient. This results from the values of the multipliers being calculated using (S1.11). The value of the reduced gradient is shown in (S1.14) for the first iteration.

$$\mathbf{r} = \begin{pmatrix} -260 \\ -40 \\ 0 \\ 0 \\ 0 \end{pmatrix} - \begin{pmatrix} -1 & 1.5 & -1 \\ -1 & 0 & -1 \\ 0 & 0 & 0 \\ 0 & 1 & 0 \\ 0 & 0 & 1 \end{pmatrix} \begin{pmatrix} 40 \\ 0 \\ 0 \end{pmatrix} = \begin{pmatrix} -220 \\ 0 \\ 0 \\ 0 \\ 0 \end{pmatrix} \qquad \text{(S1.14)}$$

Step 5: Check the stopping criterion

As the iterations proceed, points are generated which become successively closer to optimality. As these points approach optimality, the gradient of the Lagrangian (S1.5) moves closer to zero, and the magnitude of the reduced gradient decreases. At optimality, the magnitude of the reduced gradient is identically zero. Numerically, it is necessary to specify a threshold at which the algorithm terminates to a point that is "good enough". This optimality tolerance on the magnitude of the reduced gradient is a user-specified input, ε. At each iteration of the GRG algorithm, (S1.15) is checked, and once satisfied, the algorithm terminates.

$$\left\|\mathbf{r}\right\| \leq \varepsilon \qquad \text{(S1.15)}$$

At the input point, the magnitude of the reduced gradient is $\left\|\mathbf{r}_0\right\| = 220.0$.

Step 6: Find a search direction

To minimize the objective function subject to the constraints, one choice for the search direction for the nonbasic variables is $-\mathbf{r}_{nb}$, where $\mathbf{r}_{nb}$ is the reduced gradient of the nonbasic variables. This gives a steepest descent direction in the nonbasic variables, which will be used in this example. Designating the search direction as $\mathbf{s}$ gives (S1.16) for the nonbasic variables.

$$\mathbf{s}_{nb} = -\mathbf{r}_{nb} \qquad \text{(S1.16)}$$

The search direction for the basic variables is chosen with the goal of minimizing the violation of the constraints, $g(\mathbf{x}) = \mathbf{0}$. To find this direction, consider each constraint $g_i(\mathbf{x})$ to be a function of $\mathbf{x}_b$ and $\mathbf{x}_{nb}$, so that $g_i(\mathbf{x}) = g_i(\mathbf{x}_b, \mathbf{x}_{nb})$. The total differential of $g_i(\mathbf{x}_b, \mathbf{x}_{nb})$ is shown in (S1.17) with $\mathbf{x}$ partitioned in this way for a single constraint $g_i(\mathbf{x}) = g_i(\mathbf{x}_b, \mathbf{x}_{nb})$ [4].

$$dg_i(\mathbf{x}_b, \mathbf{x}_{nb}) = \left(\frac{\partial g_i}{\partial \mathbf{x}_b}\right)^T d\mathbf{x}_b + \left(\frac{\partial g_i}{\partial \mathbf{x}_{nb}}\right)^T d\mathbf{x}_{nb} = \nabla_b g_i^{\,T} d\mathbf{x}_b + \nabla_{nb} g_i^{\,T} d\mathbf{x}_{nb} \qquad \text{(S1.17)}$$

Considering the full vector of m equality constraints, $g(\mathbf{x}_b, \mathbf{x}_{nb})$, (S1.17) is generalized to (S1.18).

$$dg(\mathbf{x}_b, \mathbf{x}_{nb}) = \begin{pmatrix} -\nabla_b g_1(\mathbf{x})^T - \\ \vdots \\ -\nabla_b g_m(\mathbf{x})^T - \end{pmatrix} d\mathbf{x}_b + \begin{pmatrix} -\nabla_{nb} g_1(\mathbf{x})^T - \\ \vdots \\ -\nabla_{nb} g_m(\mathbf{x})^T - \end{pmatrix} d\mathbf{x}_{nb} = J_b(\mathbf{x}) d\mathbf{x}_b + J_{nb}(\mathbf{x}) d\mathbf{x}_{nb} \qquad \text{(S1.18)}$$

For a small finite change $\Delta\mathbf{x}$, the constraints $g(\mathbf{x}) = 0$ are satisfied to first order using (S1.19), obtained from (S1.18) by setting the constraint violation, Δg, equal to zero.

$$J_b(\mathbf{x})\Delta\mathbf{x}_b + J_{nb}(\mathbf{x})\Delta\mathbf{x}_{nb} = \mathbf{0} \qquad \text{(S1.19)}$$

Rearranging (S1.19) and substituting $\Delta\mathbf{x}_{nb} = \mathbf{s}_{nb}$ and $\Delta\mathbf{x}_b = \mathbf{s}_b$ provides a way to compute $\mathbf{s}_b$ from $\mathbf{s}_{nb}$ at each iteration (S1.20).

$$\mathbf{s}_b = -J_b^{-1} J_{nb} \mathbf{s}_{nb} \qquad \text{(S1.20)}$$

The search direction for the nonbasic variables, $\mathbf{s}_{nb}$, and basic variables, $\mathbf{s}_b$, is thereby determined for the current iteration. The full search direction, $\mathbf{s}$, is now defined. For the initial point, the search direction is $\mathbf{s}_0 = \begin{bmatrix} 220 & -220 & 0 & -330 & 0 \end{bmatrix}^T$.

Step 7: Perform a line search along the search direction. Use a pseudo-Newton process to correct any constraint violation after the step.

Using the search direction, $\mathbf{s}$, the next step is to perform a line search in the direction of $\mathbf{s}$. The next point is calculated as shown in (S1.21).

$$\mathbf{x}_{i+1} = \mathbf{x}_i + \alpha_i \mathbf{s}_i = \mathbf{x}_i + \alpha_i \left\| \mathbf{s}_i \right\| \hat{\mathbf{s}}_i \qquad \text{(S1.21)}$$

Here, $\hat{\mathbf{s}}_i$ is the unit vector in the search direction, $\mathbf{s}_i$, at iteration i and $\left\| \mathbf{s}_i \right\|$ is the magnitude of $\mathbf{s}_i$. Ideally, α is chosen to find the global minimizer of the objective along the search direction. However, this is highly computationally expensive in practice. For practical applications, a step size is accepted if it satisfies some sufficient decrease condition [5]. For the purpose of illustrating the GRG algorithm in this simple example, α is held constant at $\alpha_i = 0.001$ for each iteration. The magnitude of the reduced gradient, $\mathbf{r}$, is large far from an optimum and decreases as iterations approach an optimum. Because $\mathbf{s}$ is determined from $\mathbf{r}$, $\left\| \mathbf{s} \right\|$ will decrease with subsequent iterations, and the step length will automatically decrease as the optimum is approached despite α being held constant in the example. From the initial point, the next point is calculated as shown in (S1.22) using (S1.21).

$$\tilde{\mathbf{x}}_1 = \begin{pmatrix} 0 \\ 0.5 \\ 0 \\ 1 \\ 0.5 \end{pmatrix} + 0.001 \begin{pmatrix} 220 \\ -220 \\ 0 \\ -330 \\ 0 \end{pmatrix} = \begin{pmatrix} 0 \\ 0.5 \\ 0 \\ 1 \\ 0.5 \end{pmatrix} + 0.001 \;\; 453.54 \begin{pmatrix} 0.4851 \\ -0.4851 \\ 0 \\ -0.7276 \\ 0 \end{pmatrix} = \begin{pmatrix} 0.2200 \\ 0.2800 \\ 0 \\ 0.6700 \\ 0.5000 \end{pmatrix} \qquad \text{(S1.22)}$$

The "~" decoration is added to $\mathbf{x}_1$ in (S1.22) because this point may violate some constraints. After the step, it may be necessary to correct the constraint violation to within a tolerance. The constraint violation for $\tilde{\mathbf{x}}_1$ in the example is shown in (S1.23).

$$g(\tilde{\mathbf{x}}_1) = \begin{pmatrix} 0.0484 \\ -0.0484 \\ 0.0000 \end{pmatrix} \qquad \text{(S1.23)}$$

In the example, only the first two constraints are violated, and the constraint violation is small. To correct the constraint violation, pseudo-Newton iterations are performed until the constraint violation is satisfied within a tolerance [4]. The pseudo-Newton iterations are of the form shown in (S1.24).

$$\mathbf{y}_{i+1} = \mathbf{y}_i - J(\mathbf{y}_i)^T \left[J(\mathbf{y}_i) J(\mathbf{y}_i)^T \right]^{-1} g(\mathbf{y}_i) \qquad \text{(S1.24)}$$

The pseudo-Newton iterations are repeated until the constraints $g(\mathbf{x})$ are satisfied to within a tolerance. After performing the pseudo-Newton iterations, the new point, $\mathbf{x}_1$, is found. For the example, this point is shown in (S1.25) along with the objective function $f(\mathbf{x}_1)$.

$$\mathbf{x}_1 = \begin{pmatrix} 0.2371 \\ 0.3191 \\ 0 \\ 0.6872 \\ 0.5562 \end{pmatrix}, \quad f(\mathbf{x}_1) = 52.80 \tag{S1.25}$$

Step 8: Repeat all steps with the new point

After the new point is calculated, repeat all steps until the stopping criterion is met in **Step 5**. The results of this example are shown in **Table S1.1**. The problem is depicted graphically in **Fig S1.1**.

Table S1.1. The results of the first fifteen iterations of the GRG algorithm are shown for the example problem. The algorithm was operated with α held constant at 0.001. The iterations converge quickly to the optimum of the constrained problem. Iterations 9-15 continue to improve the objective function, however the value was rounded to three decimal places. The magnitude of the reduced gradient approaches zero as the iterations progress, and the algorithm is stopped when this value is within a user-defined tolerance.

**Iteration**	$\mathbf{x}$	$f(\mathbf{x})$	$\lVert\mathbf{r}\rVert$
0	$\begin{bmatrix}0.0000 & 0.5000 & 0.0000 & 1.0000 & 0.0000\end{bmatrix}^T$	98.00	220.0
1	$\begin{bmatrix}0.2371 & 0.3191 & 0.0000 & 0.6872 & 0.5562\end{bmatrix}^T$	52.80	167.2
2	$\begin{bmatrix}0.3999 & 0.2600 & 0.0000 & 0.4961 & 0.6599\end{bmatrix}^T$	27.93	136.8
3	$\begin{bmatrix}0.5289 & 0.2508 & 0.0000 & 0.3384 & 0.7798\end{bmatrix}^T$	12.46	100.2
4	$\begin{bmatrix}0.6238 & 0.2653 & 0.0000 & 0.2107 & 0.8891\end{bmatrix}^T$	4.761	59.67
5	$\begin{bmatrix}0.6814 & 0.2829 & 0.0000 & 0.1259 & 0.9643\end{bmatrix}^T$	2.222	27.46
6	$\begin{bmatrix}0.7084 & 0.2934 & 0.0000 & 0.0838 & 1.0018\end{bmatrix}^T$	1.713	10.05
7	$\begin{bmatrix}0.7183 & 0.2977 & 0.0000 & 0.0678 & 1.0160\end{bmatrix}^T$	1.647	3.205
8	$\begin{bmatrix}0.7215 & 0.2991 & 0.0000 & 0.0627 & 1.0206\end{bmatrix}^T$	1.640	0.9662
9	$\begin{bmatrix}0.7225 & 0.2995 & 0.0000 & 0.0611 & 1.0220\end{bmatrix}^T$	1.639	0.2857
10	$\begin{bmatrix}0.7228 & 0.2996 & 0.0000 & 0.0606 & 1.0224\end{bmatrix}^T$	1.639	0.0840
11	$\begin{bmatrix}0.7229 & 0.2997 & 0.0000 & 0.0605 & 1.0225\end{bmatrix}^T$	1.639	0.0247
12	$\begin{bmatrix}0.7229 & 0.2997 & 0.0000 & 0.0605 & 1.0226\end{bmatrix}^T$	1.639	0.0072
13	$\begin{bmatrix}0.7229 & 0.2997 & 0.0000 & 0.0605 & 1.0226\end{bmatrix}^T$	1.639	0.0021
14	$\begin{bmatrix}0.7229 & 0.2997 & 0.0000 & 0.0605 & 1.0226\end{bmatrix}^T$	1.639	0.0006
15	$\begin{bmatrix}0.7229 & 0.2997 & 0.0000 & 0.0605 & 1.0226\end{bmatrix}^T$	1.639	0.0002

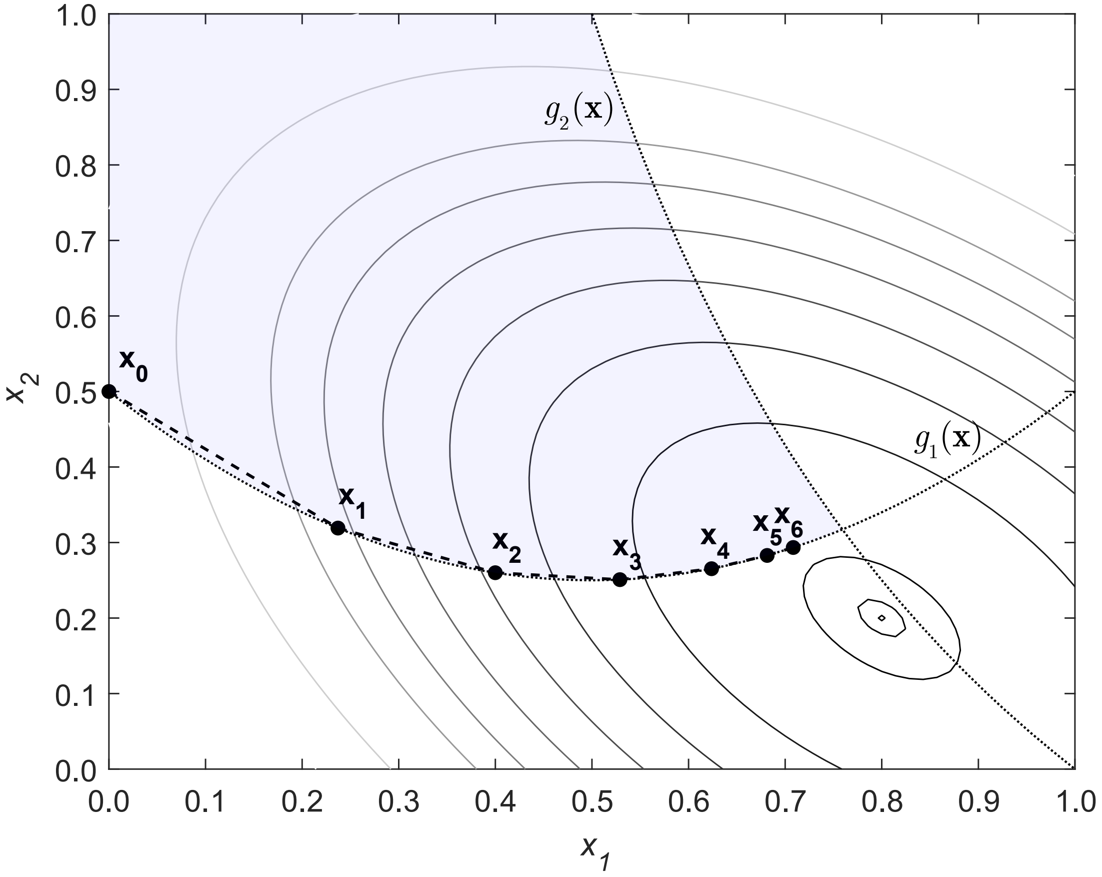

Fig S1.1. The contours of the objective function are shown for the example problem. The first two constraints, $g_1(\mathbf{x})$ and $g_2(\mathbf{x})$, are represented in two-dimensional space. In this representation, x_3^2 and x_4 can be thought of as slack variables for the two constraints, $g_1(\mathbf{x})$ and $g_2(\mathbf{x})$, respectively. Because $\mathbf{x} \geq 0$, the feasible region (shaded in blue) for x_1 and x_2 is bounded below by $g_1(\mathbf{x})$ and above by $g_2(\mathbf{x})$ in this two-dimensional space. The third constraint, $g_3(\mathbf{x})$ is not plotted because it does not constrain the feasible space. The variable x_5 may be arbitrarily increased to satisfy $g_3(\mathbf{x})$ for any values of x_1 and x_2. The first six iterations of the GRG algorithm are plotted for the example problem. The points progress along the boundary of $g_1(\mathbf{x})$and approach the minimum of the objective function as closely as possible without violating the constraints. In this case, the optimum of the objective function lies outside the feasible region of the constraints, therefore the optimal solution of the constrained problem is not the same as the optimum of the unconstrained problem.

References

1. Abadie J, Carpentier J. Generalization of the Wolfe Reduced Gradient Method to the Case of Nonlinear Constraints. Optimization. Academic Press; 1969.
2. Drud A. CONOPT: A GRG code for large sparse dynamic nonlinear optimization problems. Math Program. 1985;31: 153–191. doi:10/bhwtmp
3. Drud AS. CONOPT—A Large-Scale GRG Code. ORSA J Comput. 1994;6: 207–216. doi:10/cww2m7
4. Arora J. Introduction to Optimum Design, 4th ed. London, UK: Academic Press; 2017.
5. Nocedal J, Wright S. Numerical Optimization. Springer Science+Business Media, LLC; 2006.
