# Supplementary material for: Isotope-assisted metabolic flux analysis as an equality-constrained nonlinear program for improved scalability and robustness
Source: PLoS Comput Biol. 2022 Mar 24;18(3):e1009831. doi: 10.1371/journal.pcbi.1009831 (PMC8947808; doi:10.1371/journal.pcbi.1009831)
Supplement: S2 Text — (PDF) [file pcbi.1009831.s002.pdf]

Maintenance of Constraint Feasibility in the CONOPT Algorithm on GAMS

The following mathematical details on the internal workings of the CONOPT algorithm are based on a communication with Dr. Arne Drud (ARKI Consulting & Development A/S, Denmark). Further mathematical detail is available in Dr. Drud's publications [1,2].

An equality-constrained NLP can be modeled as follows, in which the optimization variables are partitioned into two sets **x** (basic variables) and **y** (nonbasic variables):

$$\begin{aligned} \min \quad & f(\mathbf{x},\mathbf{y}) \\ s.t. \quad & \mathbf{g}(\mathbf{x},\mathbf{y}) = \mathbf{0} \end{aligned} \qquad [1]$$

The scalar objective $f(\mathbf{x},\mathbf{y})$ and the vector of equality constraints $\mathbf{g}(\mathbf{x},\mathbf{y}) = \mathbf{0}$ are nonlinear in $\mathbf{x}$ and $\mathbf{y}$. In an inst-MFA problem, most of the equality constraints correspond to the steady-state or discretized transient balances on cumomers/EMUs. The remaining equality constraints are generated from the inequality constraints (e.g., bounds on fluxes) by introducing slack variables, a step that is handled by CONOPT.

The vector **x** contains the basic variables, and the vector **y** contains the nonbasic variables. During an iteration of the optimization, the step size and direction are determined by using the nonbasic variables $\mathbf{y}$. The basic variables **x** are updated to satisfy the equality constraints. In this formulation, the number of basic variables is equal to the number of constraints (typically ~10^5 for a large inst-MFA model). The number of nonbasic variables is approximately equal to the number of unknown parameters (~100 fluxes and pool sizes for a typical inst-MFA model). As the algorithm proceeds, the partitioning of the variables into basic and nonbasic may change at each iteration. This partitioning is handled by CONOPT and is done without distinction between parameters and state variables. See Drud [2] for further details on basic/nonbasic variables and details of the basis factorization implemented in CONOPT.

Expressing the Objective as a Function of the Nonbasic Variables y

At a feasible point $(\mathbf{x}_0, \mathbf{y}_0)$:

$$\mathbf{g}(\mathbf{x},\mathbf{y}) = \mathbf{0} \qquad [2]$$

According to the implicit function theorem [3], if there is a feasible point $(\mathbf{x}_0, \mathbf{y}_0)$ where the Jacobian $\partial \mathbf{g}/\partial \mathbf{x}$ is nonsingular, then **x** can be expressed as a function of **y** in the neighborhood of the feasible point $(\mathbf{x}_0, \mathbf{y}_0)$:

$$\mathbf{x} = \mathbf{G}(\mathbf{y}) \qquad [3]$$

From [2] and [3]:

$$\mathbf{g}(\mathbf{G}(\mathbf{y}),\mathbf{y}) \equiv \mathbf{0} \qquad [4]$$

Differentiating [4] via the chain rule, we get:

$$\frac{\partial \mathbf{g}}{\partial \mathbf{G}} \cdot \frac{\partial \mathbf{G}}{\partial \mathbf{y}} + \frac{\partial \mathbf{g}}{\partial \mathbf{y}} \equiv \mathbf{0}$$

Thus:

$$\frac{\partial \mathbf{G}}{\partial \mathbf{y}} = -\left(\frac{\partial \mathbf{g}}{\partial \mathbf{G}}\right)^{-1} \cdot \frac{\partial \mathbf{g}}{\partial \mathbf{y}} \qquad [5]$$

From [3] and [5]:

$$\frac{\partial \mathbf{x}}{\partial \mathbf{y}} = -\left(\frac{\partial \mathbf{g}}{\partial \mathbf{x}}\right)^{-1} \cdot \frac{\partial \mathbf{g}}{\partial \mathbf{y}} \qquad [6]$$

As shown below, we can substitute [6] into the expression for the objective function gradient to eliminate **x** from the gradient and obtain an expression for it involving **y** alone.

Because of [3], the NLP [1] can be written as:

$$\begin{aligned} \min \quad & F(\mathbf{y}) = f(\mathbf{G}(\mathbf{y}),\mathbf{y}) \\ s.t. \quad & \mathbf{g}(\mathbf{G}(\mathbf{y}),\mathbf{y}) = \mathbf{0} \end{aligned}$$

The derivative of the objective function w.r.t. **y** is:

$$\begin{aligned} \frac{\partial F}{\partial \mathbf{y}} &= \frac{\partial f}{\partial \mathbf{x}} \cdot \frac{\partial \mathbf{x}}{\partial \mathbf{y}} + \frac{\partial f}{\partial \mathbf{y}} \\ \frac{\partial F}{\partial \mathbf{y}} &= -\frac{\partial f}{\partial \mathbf{x}} \cdot \left(\frac{\partial \mathbf{g}}{\partial \mathbf{x}}\right)^{-1} \cdot \frac{\partial \mathbf{g}}{\partial \mathbf{y}} + \frac{\partial f}{\partial \mathbf{y}} \end{aligned} \quad [7]$$

Thus, we now have a problem in **y** alone (dimension~100). Eqs. [7] enable us to compute both the objective F and its gradient $\partial F/\partial \mathbf{y}$.

The computation of the matrix inverse $\partial \mathbf{g}/\partial \mathbf{x}^{-1}$ may appear to be expensive given its size of $10^5 \times 10^5 = 10^{10}$ elements. However, this matrix has a special structure due to the discretized ODEs. Thus, most off-diagonal elements in $\partial \mathbf{g}/\partial \mathbf{x}$ are zero. CONOPT is able to exploit this structure to efficiently invert $\partial \mathbf{g}/\partial \mathbf{x}$. An average constraint has only 5–10 nonzero elements, giving a total of $O(10^6)$ elements. For the Synechocystis genome-scale inst-MFA model described in the manuscript, the Jacobian had ~1.6×10^6 nonzero elements when using the 9th-order Radau IIA collocation method (we obtained the Jacobian size from the GAMS model generation statistics). CONOPT has an efficient algorithm for computing the inverse of $\partial \mathbf{g}/\partial \mathbf{x}$. This algorithm is based on the LU decomposition method wherein **L** and **U** are sparse triangular matrices, each containing only have a few million nonzero elements. Thus, any operation involving $\partial \mathbf{g}/\partial \mathbf{x}^{-1}$ can be rewritten in terms of **L** and **U**, and be performed with $O(10^6)$ operations. The LU factorization typically needs < 50 million operations.

Ensuring Feasibility at Each Iteration

The GRG algorithm and CONOPT use Newton's method, which computes the residuals in $\mathbf{g}(\mathbf{x},\mathbf{y})$ and adjusts **x** iteratively by using a linear approximation and the inverse $\partial \mathbf{g}/\partial \mathbf{x}^{-1}$. Thus:

$$g(\mathbf{x},\mathbf{y}) + \frac{\partial \mathbf{g}}{\partial \mathbf{x}} \cdot d\mathbf{x} = \mathbf{0}$$

$$d\mathbf{x} = -\left(\frac{\partial \mathbf{g}}{\partial \mathbf{x}}\right)^{-1} \cdot g(\mathbf{x},\mathbf{y})$$

$$\mathbf{x} := \mathbf{x} + d\mathbf{x}$$

The above steps are repeated until $\mathbf{g}(\mathbf{x},\mathbf{y})$ is sufficiently small, i.e., within a tolerance. Typically, this procedure requires only a few (5 to 10) iterations of Newton's method as **x** is simply being updated from its previous value to satisfy the constraints at the current iteration. The repeated use of the inverse $\partial \mathbf{g}/\partial \mathbf{x}^{-1}$ renders this method efficient. Once $\mathbf{g}(\mathbf{x},\mathbf{y})$ is within the preset tolerance, the objective function is evaluated at this point.

Dynamic Tolerances

CONOPT minimizes the number of evaluations of $\mathbf{g}(\mathbf{x},\mathbf{y})$ by setting dynamic tolerances in the Newton iterations. During the early stages of the optimization, $\mathbf{g}(\mathbf{x},\mathbf{y})$ is not solved exactly as the algorithm needs to proceed to new points in quick succession. This minimizes the number of Newton iterations needed at this stage. During the later stages, as the optimum is approached, $\mathbf{g}(\mathbf{x},\mathbf{y})$ is solved with high accuracy. However, the optimization is making very small moves at this stage as $\mathbf{x}$ is already close to the solution. Therefore, the increased accuracy is not expensive during the final stages. We note that the default values of the maximum and minimum feasibility tolerances in CONOPT are 1×10^{-7} and 4×10^{-10} respectively. With these values, we have very rarely seen a loss of feasibility in eiFlux.

Searching in the y Space

To search in the $\mathbf{y}$ space, CONOPT the gradient $\partial F/\partial \mathbf{y}$ and the reduced Hessian $\partial^2 F/\partial \mathbf{y}^2$. The name "reduced Hessian" stems from the fact that this Hessian (matrix of second derivatives) in calculated in the reduced $\mathbf{y}$ space. This matrix is typically quite small containing $\sim 10^2 \times 10^2 = 10^4$ elements for an inst-MFA problem. This reduced Hessian is efficiently and inexpensively computed from the changes in $\partial F/\partial \mathbf{y}$ for steps taken in the $\mathbf{y}$ space. This computation makes use of techniques such as quasi-Newton methods or Broyden-Fletcher-Goldfarb-Shanno (BFGS) updates.

References

1. Drud A. CONOPT: A GRG code for large sparse dynamic nonlinear optimization problems. Math Program. 1985;31: 153–191. doi:10/bhwtmp

2. Drud A. CONOPT—A Large-Scale GRG Code. ORSA J Comput. 1994;6: 207–216. doi:10/cww2m7

3. Stewart J. Multivariable Calculus. 7th ed. Belmont, CA: Brooks/Cole; 2012.
