# Supplementary material for: Isotope-assisted metabolic flux analysis as an equality-constrained nonlinear program for improved scalability and robustness
Source: PLoS Comput Biol. 2022 Mar 24;18(3):e1009831. doi: 10.1371/journal.pcbi.1009831 (PMC8947808; doi:10.1371/journal.pcbi.1009831)
Supplement: S3 Text — (PDF) [file pcbi.1009831.s003.pdf]

Simple Isotopomer Network to Illustrate Collocation

To illustrate how a collocation method may be used to approximate the solution to a system of ordinary differential equations (ODEs), we consider a simple example possessing an analytical (exact) solution (**Fig S2.1**). This example was chosen because it represents a very simple isotope labeling experiment, where the governing isotopomer balancing equations [1,2] constitute a linear ODE system that may be solved analytically to give an exact solution in terms of eigenvalues and eigenvectors. The collocation method developed here may be extended to any arbitrary unsteady state isotopomer network, where an analytical solution is not easily accessible.

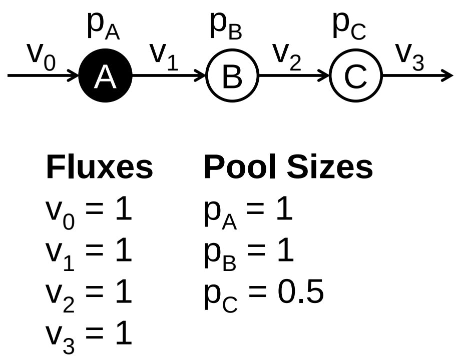

Fig S2.1. In this simple example network consisting of three 1-carbon compounds, A is 100% ^{13}C labeled. Compounds B and C are initially 100% ^{12}C but over time, they will become ^{13}C-enriched. The goal is to calculate the ^{13}C labeling profile over time for metabolites B and C.

Exact Solution

In the network shown in **Fig S2.1**, the flux values and pool sizes are given. Therefore, the solution may be obtained by solving the isotopically nonstationary isotopomer balances with these parameter values. The isotopomer balances for this system are shown in (S2.1).

$$\begin{aligned} 1\frac{db_0}{dt} &= -b_0, \quad b_0(t=0)=1 \\ 1\frac{db_1}{dt} &= 1-b_1, \quad b_1(t=0)=0 \\ 0.5\frac{dc_0}{dt} &= b_0-c_0, \quad c_0(t=0)=1 \\ 0.5\frac{dc_1}{dt} &= b_1-c_1, \quad c_1(t=0)=0 \end{aligned} \tag{S2.1}$$

With $\mathbf{x}^T = \begin{bmatrix} b_0 & b_1 & c_0 & c_1 \end{bmatrix}$, this system of equations can be written in matrix notation (S2.2).

$$\frac{d\mathbf{x}}{dt} = f(\mathbf{x}) = \mathbf{M}\mathbf{x} + \mathbf{b} = \begin{pmatrix} -1 & 0 & 0 & 0 \\ 0 & -1 & 0 & 0 \\ 2 & 0 & -2 & 0 \\ 0 & 2 & 0 & -2 \end{pmatrix}\mathbf{x} + \begin{pmatrix} 0 \\ 1 \\ 0 \\ 0 \end{pmatrix}, \quad \mathbf{x}_{t=0} = \begin{pmatrix} 1 \\ 0 \\ 1 \\ 0 \end{pmatrix} \tag{S2.2}$$

With the substitution $\mathbf{x}^T = \mathbf{y}^T + \begin{bmatrix} 0 & 1 & 0 & 1 \end{bmatrix}$, (S2.2) is converted into a homogenous linear system whose exact solution can be written using the matrix eigenvalues and eigenvectors (S2.3). The solution is plotted in **Fig S2.2** on the time interval [0,2].

$$\mathbf{x}(t) = \begin{pmatrix} 0 \\ 0 \\ -1 \\ 1 \end{pmatrix} e^{-2t} + \begin{pmatrix} 1 \\ -1 \\ 2 \\ -2 \end{pmatrix} e^{-t} + \begin{pmatrix} 0 \\ 1 \\ 0 \\ 1 \end{pmatrix} \tag{S2.3}$$

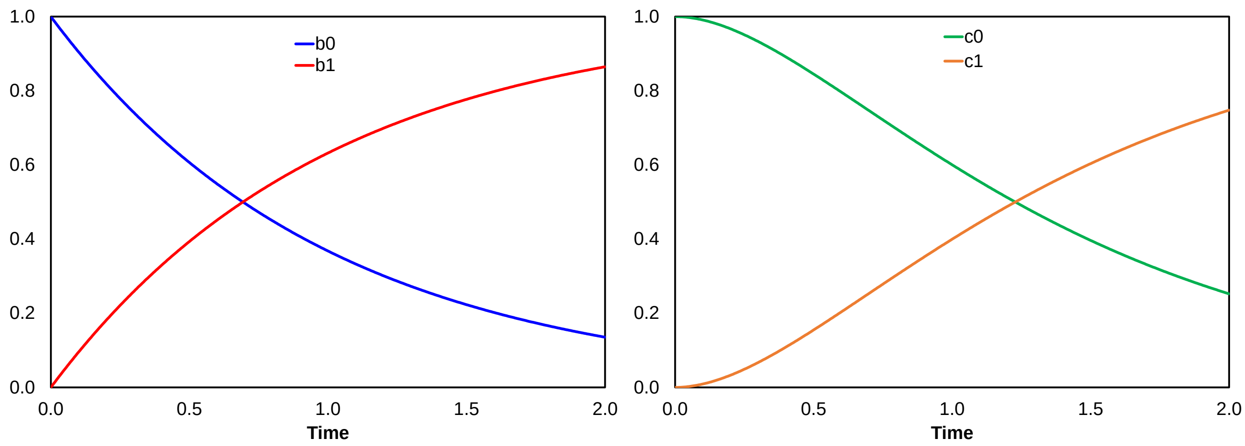

Fig S2.2. The exact solution to (S2.2) is plotted on the time interval [0,2]. Clearly, the ^{13}C enrichment of compounds B and C is initially zero but increases with time. At infinite time, B and C become fully enriched.

Collocation Solution

Here, we will solve (S2.2) using the 5th-order Radau IIA orthogonal collocation method [3,4], then compare the resulting approximation to the exact solution. In this method, the time domain is first divided into several contiguous intervals, after which the solution is approximated within each time interval. For this illustrative example, the time domain is divided into two contiguous intervals [0, 1] and [1, 2]. For a given time interval, the equations to be solved are those for the corresponding fully-implicit Runge-Kutta method.

$$\begin{aligned}
\mathbf{K}_1 &= \mathbf{x}_0 + h(a_{11}\mathbf{k}_1 + a_{12}\mathbf{k}_2 + a_{13}\mathbf{k}_3) \\
\mathbf{K}_2 &= \mathbf{x}_0 + h(a_{21}\mathbf{k}_1 + a_{22}\mathbf{k}_2 + a_{23}\mathbf{k}_3) \\
\mathbf{K}_3 &= \mathbf{x}_0 + h(a_{31}\mathbf{k}_1 + a_{32}\mathbf{k}_2 + a_{33}\mathbf{k}_3) \\
\mathbf{k}_1 &= f(\mathbf{K}_1, t_0 + h) = \mathbf{M}\cdot\mathbf{K}_1 + \mathbf{b} \\
\mathbf{k}_2 &= f(\mathbf{K}_2, t_0 + h) = \mathbf{M}\cdot\mathbf{K}_2 + \mathbf{b} \\
\mathbf{k}_3 &= f(\mathbf{K}_3, t_0 + h) = \mathbf{M}\cdot\mathbf{K}_3 + \mathbf{b}
\end{aligned} \tag{S2.4}$$

In (S2.4), $\mathbf{x}_0$ is the value of $\mathbf{x}$ at the initial point, t_0, in that time interval. The step size, h, is the width of the time interval being considered. The values of a_{ij} are determined from row i and column j of $\mathbf{A}$, the Runge-Kutta matrix for the corresponding Runge-Kutta Method (S2.5). For an explanation of how these a_{ij} are derived, see Huynh [4].

$$\mathbf{A} = \left(\begin{array}{c|c|c} \frac{11}{45} - \frac{7\sqrt{6}}{360} & \frac{37}{225} - \frac{169\sqrt{6}}{1800} & -\frac{2}{225} + \frac{\sqrt{6}}{75} \\ \hline \frac{37}{225} + \frac{169\sqrt{6}}{1800} & \frac{11}{45} + \frac{7\sqrt{6}}{360} & -\frac{2}{225} - \frac{\sqrt{6}}{75} \\ \hline \frac{4}{9} - \frac{\sqrt{6}}{36} & \frac{4}{9} + \frac{\sqrt{6}}{36} & \frac{1}{9} \end{array}\right) \tag{S2.5}$$

The set of equations (S2.4) are a fully implicit, exactly determined algebraic system with unknown vectors $\mathbf{K}_1, \mathbf{K}_2, \mathbf{K}_3$ and $\mathbf{k}_1, \mathbf{k}_2, \mathbf{k}_3$. This can be solved using a root-finding algorithm such as Newton's Method. The vectors $\mathbf{K}_1, \mathbf{K}_2, \mathbf{K}_3$ are the values of $\mathbf{x}$ at each of the three collocation points in the time interval. For the 5th-order Radau IIA method, these collocation points are $\mathbf{c} = \left(\frac{2}{5} - \frac{\sqrt{6}}{10}, \ \ \frac{2}{5} + \frac{\sqrt{6}}{10}, \ \ 1 \right)$ on the interval [0,1]. Note that for Radau IIA methods, the interval endpoint is also a collocation point. The vectors $\mathbf{k}_1 \ldots \mathbf{k}_3$ contain the basis coefficients for representing the solution in that time interval as a linear combination of the basis functions $B_1(t), B_2(t), B_3(t)$, defined in (S2.6) on the interval [0,1]. In (S2.6), $\ell_i(t)$ are the Lagrange polynomials defined using the collocation points. These basis functions are listed explicitly in (S2.7) and plotted in **Fig S2.3**.

$$B_i(t) = \int_0^t \ell_i(t')\, dt' = \int_0^t \left(\prod_{j=1, i \neq j}^{s} \frac{t' - c_j}{c_i - c_j} \right) dt' \qquad \text{(S2.6)}$$

$$\begin{aligned}
B_1(t) &= \frac{1}{3}\left(\frac{1}{c_1 - c_2}\right)\left(\frac{1}{c_1 - c_3}\right)t^3 - \frac{1}{2}(c_2 + c_3)t^2 + c_2 c_3 t \\
B_2(t) &= \frac{1}{3}\left(\frac{1}{c_2 - c_1}\right)\left(\frac{1}{c_2 - c_3}\right)t^3 - \frac{1}{2}(c_1 + c_3)t^2 + c_1 c_3 t \\
B_2(t) &= \frac{1}{3}\left(\frac{1}{c_3 - c_1}\right)\left(\frac{1}{c_3 - c_2}\right)t^3 - \frac{1}{2}(c_1 + c_2)t^2 + c_1 c_2 t
\end{aligned} \qquad \text{(S2.7)}$$

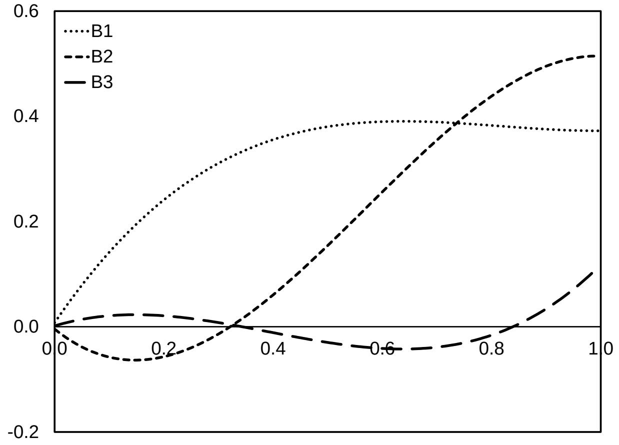

Fig S2.3. The basis functions used for generating a continuous approximation to the ODE solution in a given time interval. These basis functions were obtained by integrating the three Lagrange polynomials generated using the collocation points, which are the zeros of the corresponding Radau polynomial.

The following solution is obtained by solving (S2.4) on the time interval [0,1]:

$$\mathbf{K}_1 = \begin{pmatrix} 0.8569 \\ 0.1431 \\ 0.9755 \\ 0.0245 \end{pmatrix}, \quad \mathbf{K}_2 = \begin{pmatrix} 0.5242 \\ 0.4758 \\ 0.7776 \\ 0.2224 \end{pmatrix}, \quad \mathbf{K}_3 = \begin{pmatrix} 0.3679 \\ 0.6321 \\ 0.5995 \\ 0.4005 \end{pmatrix} \qquad \text{(S2.8)}$$

$$\mathbf{k}_1 = \begin{pmatrix} -0.8569 \\ 0.8569 \\ -0.2372 \\ 0.2372 \end{pmatrix}, \quad \mathbf{k}_2 = \begin{pmatrix} -0.5242 \\ 0.5242 \\ -0.5069 \\ 0.5069 \end{pmatrix}, \quad \mathbf{k}_3 = \begin{pmatrix} -0.3679 \\ 0.3679 \\ -0.4631 \\ 0.4631 \end{pmatrix} \qquad \text{(S2.9)}$$

The continuous approximation to the ODE solution is plotted in **Fig S2.4** for time intervals [0,1] and [1,2].

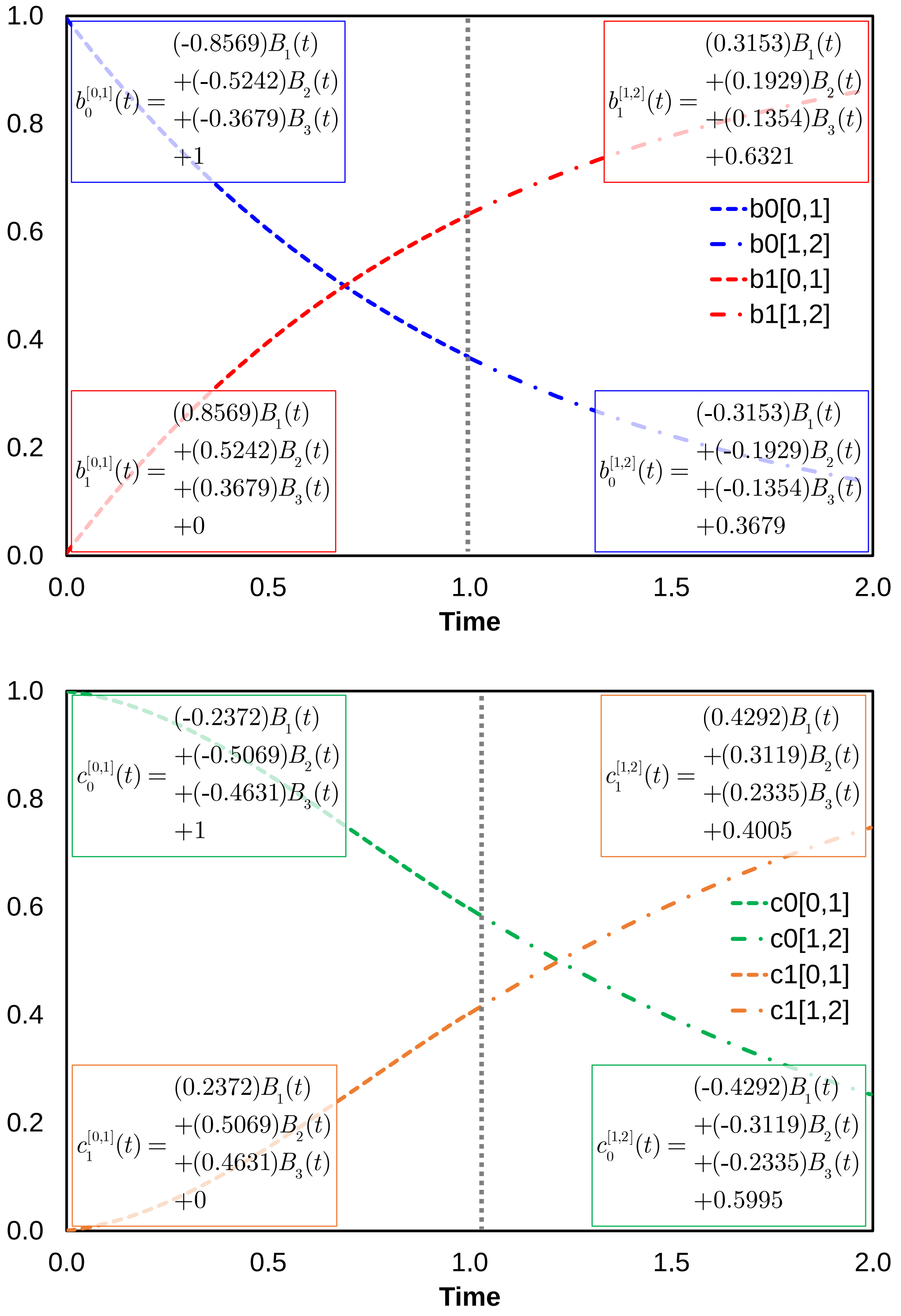

Fig S2.4. 5th-order Radau IIA collocation solution to the example ODE system. The continuous solution in each time interval is approximated by a linear combination of a set of basis polynomials. (Top) The collocation solution for compound B on the time interval [0,2]. (Bottom) The collocation solution for compound C on the time interval [0,2].

A comparison between the collocation approximation and the exact solution to the ODE system is shown in **Fig S2.5**. Clearly, the 5th-order Radau IIA collocation method accurately approximates the labeling

dynamics. If a more accurate solution is desired, a higher order collocation method (i.e. 9th-order Radau IIA) may be used, or the time domain could be further divided, and the solution approximated on shorter time intervals.

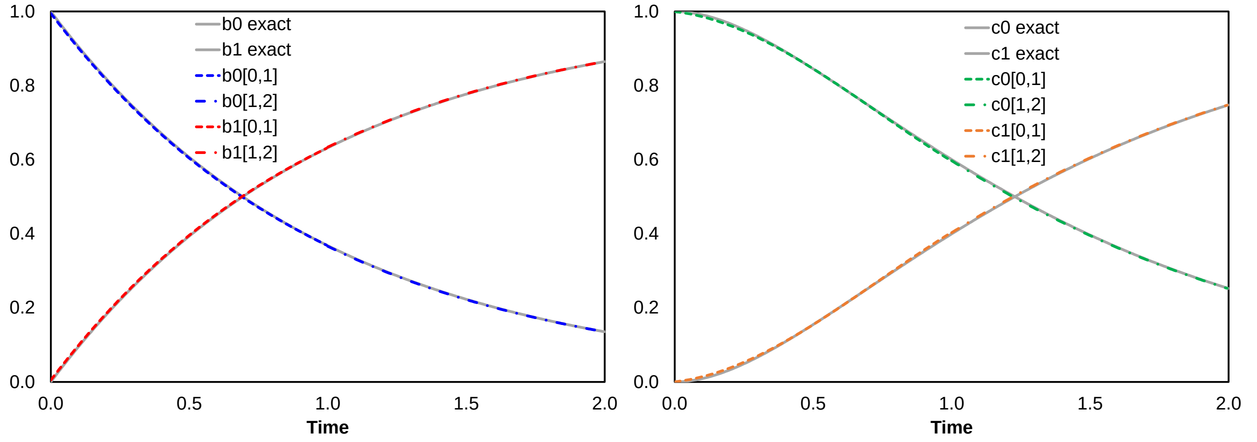

Fig S2.5. The collocation approximation is overlain on the exact solution to compare the accuracy of the method. Clearly, the 5th-order Radau IIA method provides an accurate approximation to the labeling dynamics.

In this example, we demonstrated how a collocation method may be used to approximate the solution to a system of ODEs. We demonstrated that the 5th-order Radau IIA orthogonal collocation method generated an accurate, continuous approximation to the labeling dynamics of a simple system in terms of a set of basis polynomials. When applied to the NLP formulation of the inverse problem of predicting the fluxes and pool sizes from a set of measurements, collocation methods provide a way to systematically discretize the ODE system on the entire time domain and approximate the solution at the measured time points. However, in this case the solution in each time interval is no longer solved sequentially. The entire system is solved simultaneously during the convex optimization algorithm.

References

1. Wiechert W, Möllney M, Isermann N, Wurzel M, de Graaf AA. Bidirectional reaction steps in metabolic networks: III. Explicit solution and analysis of isotopomer labeling systems. Biotechnol Bioeng. 1999;66: 69–85. doi:10.1002/(SICI)1097-0290(1999)66:2<69::AID-BIT1>3.0.CO;2-6

2. Nöh K, Wahl A, Wiechert W. Computational tools for isotopically instationary 13C labeling experiments under metabolic steady state conditions. Metab Eng. 2006;8: 554–577. doi:10/bxd49p

3. Hairer E, Wanner G. Stiff differential equations solved by Radau methods. J Comput Appl Math. 1999;111: 93–111. doi:10/b3jgts

4. Huynh HT. Collocation and Galerkin Time-Stepping Methods. 19th AIAA Computational Fluid Dynamics. San Antonio, Texas: American Institute of Aeronautics and Astronautics; 2009. doi:10/gg4rxz
