# Supplementary material for: Isotope-assisted metabolic flux analysis as an equality-constrained nonlinear program for improved scalability and robustness
Source: PLoS Comput Biol. 2022 Mar 24;18(3):e1009831. doi: 10.1371/journal.pcbi.1009831 (PMC8947808; doi:10.1371/journal.pcbi.1009831)
Supplement: S4 Text — (PDF) [file pcbi.1009831.s004.pdf]

Toy Network and *E. coli* Tandem Mass Spectrometry inst-MFA Examples

Toy Network Model

TOY NETWORK

Reaction Name	Reaction Description	Flux LB	Flux UB
v0	→ Axt (abc)	1.0	1.0
v1	Axt (abc) → A (abc)	0.0	1000.0
v2	A (abc) → C (a) + D (bc)	0.0	1000.0
v3	C (a) + D (bc) → B (abc)	0.0	1000.0
v4	A (abc) → E (ab) + F (c)	0.0	1000.0
v5	E (ab) + F (c) → B (abc)	0.0	1000.0
v6.f	A (abc) → B (abc)	0.0	1000.0
v6.b	B (abc) → A (abc)	0.0	1000.0
v7	B (abc) →	0.0	1000.0

Toy Network Results

Simulated measurements that were fit to the model are listed in **S1 Data**. All pool sizes were constrained by a lower bound of 0.0001 and an upper bound of 10.0 when fitting the model. The time domain was discretized by assigning time nodes at values of 0.0, 1.0, 2.0, 4.0, 8.0, and 20.0. The 5th-order Radau IIA collocation method was used.

Reaction Name	Actual Flux	Actual Exchange	Predicted Flux	Predicted Exchange
v0	1.000		1.000	
v1	1.000		1.000	
v2	0.700		0.695	
v3	0.700		0.695	
v4	0.100		0.100	
v5	0.100		0.100	
v6	0.200	0.800	0.205	0.775
v7	1.000		1.000	

Metabolite Name	Actual Pool Size	Predicted Pool Size
A	1.000	1.003
B	0.200	0.188
C	5.000	5.010
D	0.100	0.108
E	0.500	0.502
F	0.001	0.003

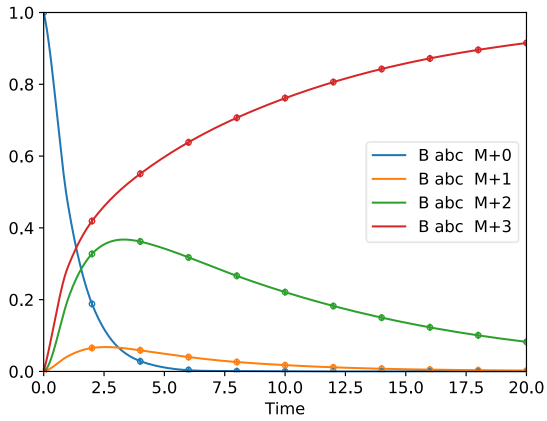
B abc M+0
B abc M+1
B abc M+2
B abc M+3
Time

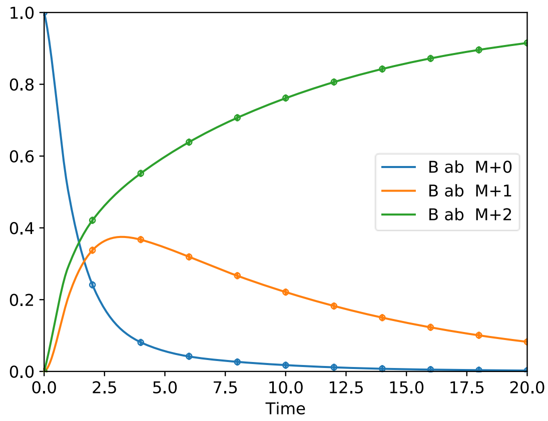
B ab M+0
B ab M+1
B ab M+2
Time

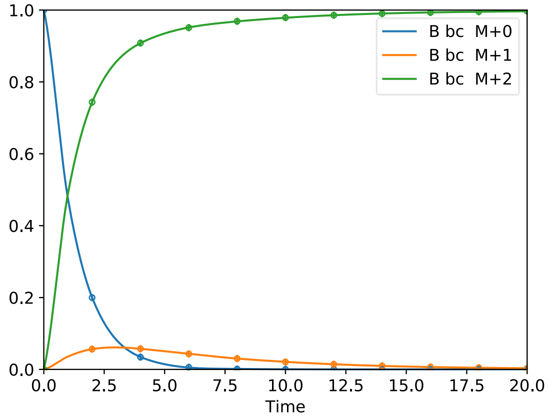
B bc M+0
B bc M+1
B bc M+2
Time

E. coli Model

The following *E. coli* metabolic flux analysis (MFA) network was adapted from that presented by Young et al. [1]. For fitting the model, the pool size of each metabolite was given a lower bound of 0.001 and an upper bound of 10.0. Confidence intervals were estimated by performing Monte Carlo simulations using the measurement means and standard deviations.

GLYCOLYSIS

Reaction Name	Reaction Description	Flux LB	Flux UB
gl-in:	→ Glc (abcdef)	1.0	1.0
gl-1:	Glc (abcdef) → G6P (abcdef)	0.0	1000.0
gl-2:	G6P (abcdef) → F6P (abcdef)	0.0	1000.0
gl-3.f:	F6P (abcdef) → FBP (abcdef)	0.0	1000.0
gl-3.b:	FBP (abcdef) → F6P (abcdef)	0.0	1000.0
gl-4.f:	FBP (abcdef) → DHAP (cba) + GAP (def)	0.0	1000.0
gl-4.b:	DHAP (cba) + GAP (def) → FBP (abcdef)	0.0	1000.0
gl-5.f:	DHAP (abc) → GAP (abc)	0.0	1000.0
gl-5.b:	GAP (abc) → DHAP (abc)	0.0	1000.0
gl-6.f:	GAP (abc) → BPG (abc)	0.0	1000.0
gl-6.b:	BPG (abc) → GAP (abc)	0.0	1000.0
gl-7.f:	BPG (abc) → 3PG (abc)	0.0	1000.0
gl-7.b:	3PG (abc) → BPG (abc)	0.0	1000.0
gl-8.f:	3PG (abc) → PEP (abc)	0.0	1000.0
gl-8.b:	PEP (abc) → 3PG (abc)	0.0	1000.0
gl-9	PEP (abc) → Pyr (abc)	0.0	1000.0

PENTOSE PHOSPHATE PATHWAY

Reaction Name	Reaction Description	Flux LB	Flux UB
pp-1:	G6P (abcdef) → 6PG (abcdef)	0.0	1000.0
pp-2:	6PG (abcdef) → Ru5P (bcdef) + CO2 (a)	0.0	1000.0
pp-3.f:	Ru5P (abcde) → R5P (abcde)	0.0	1000.0
pp-3.b:	R5P (abcde) → Ru5P (abcde)	0.0	1000.0
pp-4.f:	Ru5P (abcde) → X5P (abcde)	0.0	1000.0
pp-4.b:	X5P (abcde) → Ru5P (abcde)	0.0	1000.0
pp-5.f:	R5P (cdefg) + X5P (abhij) → S7P (abcdefg) + GAP (hij)	0.0	1000.0
pp-5.b:	S7P (abcdefg) + GAP (hij) → R5P (cdefg) + X5P (abhij)	0.0	1000.0
pp-6.f:	GAP (def) + S7P (abcghij) → F6P (abcdef) + E4P (ghij)	0.0	1000.0
pp-6.b:	F6P (abcdef) + E4P (ghij) → GAP (def) + S7P (abcghij)	0.0	1000.0
pp-7.f:	X5P (abcde) + E4P (fghi) → GAP (cde) + F6P (abfghi)	0.0	1000.0
pp-7.b:	GAP (cde) + F6P (abfghi) → X5P (abcde) + E4P (fghi)	0.0	1000.0

ENTNER-DOUDOROFF PATHWAY

Reaction Name	Reaction Description	Flux LB	Flux UB
ed-1:	6PG (abcdef) → KDPG (abcdef)	0.0	1000.0

ed-2:	KDPG (abcdef) → GAP (def) + Pyr (abc)	0.0	1000.0

TCA CYCLE

Reaction Name	Reaction Description	Flux LB	Flux UB
pd:	Pyr (abc) → CO2 (a) + AcCoA (bc)	0.0	1000.0
tc-1:	OAA (abcd) + AcCoA (pq) → Cit (dcbqpa)	0.0	1000.0
tc-2:	Cit (abcdef) → iCit (abcdef)	0.0	1000.0
tc-3:	iCit (abcdef) → aKG (abcde) + CO2 (f)	0.0	1000.0
tc-4:	aKG (abcde) → CO2 (a) + SucCoA (bcde)	0.0	1000.0
tc-5:	SucCoA (abcd) → 0.5 Suc (abcd) + 0.5 Suc (dcba)	0.0	1000.0
tc-6.f:	Suc (abcd) → 0.5 Fum (abcd) + 0.5 Fum (dcba)	0.0	1000.0
tc-6.b:	Fum (abcd) → 0.5 Suc (abcd) + 0.5 Suc (dcba)	0.0	1000.0
tc-7.f:	Fum (abcd) → Mal (abcd)	0.0	1000.0
tc-7.b:	Mal (abcd) → 0.5 Fum (abcd) + 0.5 Fum (dcba)	0.0	1000.0
tc-8.f:	Mal (abcd) → OAA (abcd)	0.0	1000.0
tc-8.b:	OAA (abcd) → Mal (abcd)	0.0	1000.0

ANAPLEROTIC/CATAPLEROTIC REACTIONS

Reaction Name	Reaction Description	Flux LB	Flux UB
me:	Mal (abcd) → Pyr (abc) + CO2 (d)	0.0	1000.0
an.f:	PEP (abc) + CO2 (d) → OAA (abcd)	0.0	1000.0
an.b:	OAA (abcd) → PEP (abc) + CO2 (d)	0.0	1000.0

ACETATE SYNTHESIS

Reaction Name	Reaction Description	Flux LB	Flux UB
ac:	AcCoA (ab) → Ac (ab)	0.0	1000.0

GLYOXYLATE SHUNT

Reaction Name	Reaction Description	Flux LB	Flux UB
gx-1:	iCit (abcdef) → Gox (ab) + 0.5 Suc (fcde) + 0.5 Suc (edcf)	0.0	1000.0
gx-2:	Gox (ab) + AcCoA (cd) → Mal (abdc)	0.0	1000.0

ENTRY/EXIT FLUXES

Reaction Name	Reaction Description	Flux LB	Flux UB
co2-in.f:	→ CO2 (a)	0.0	1000.0
co2-in.b:	CO2 (a) →	0.0	1000.0
ac-out:	Ac (ab) →	0.0	1000.0

AMINO ACID SYNTHESIS

Reaction Name	Reaction Description	Flux LB	Flux UB
glusyn:	aKG (abcde) → Glu (abcde)	0.0	1000.0
glnsyn:	Glu (abcde) → Gln (abcde)	0.0	1000.0
prosyn:	Glu (abcde) → Pro (abcde)	0.0	1000.0
argsyn:	Glu (abcde) + CO2 (f) + Gln (ghijk) + Asp (lmno) + AcCoA (pq) → Arg (abcdef) + aKG (ghijk) + Fum (lmno) + Ac (pq)	0.0	1000.0
aspsyn:	OAA (abcd) + Glu (efghi) → Asp (abcd) + aKG (efghi)	0.0	1000.0
asnsyn:	Asp (abcd) → Asn (abcd)	0.0	1000.0

alasyn:	Pyr (abc) + Glu (defgh) → Ala (abc) + aKG (defgh)	0.0	1000.0
sersyn:	3PG (abc) + Glu (defgh) → Ser (abc) + aKG (defgh)	0.0	1000.0
glysyn.f:	Ser (abc) → Gly (ab) + MEETHF (c)	0.0	1000.0
glysyn.b:	Gly (ab) + MEETHF (c) → Ser (abc)	0.0	1000.0
glydeg.f:	Gly (ab) → CO_2 (a) + MEETHF (b)	0.0	1000.0
glydeg.b:	CO_2 (a) + MEETHF (b) → Gly (ab)	0.0	1000.0
thrdeg:	Thr (abcd) → Gly (ab) + AcCoA (cd)	0.0	1000.0
cyssyn:	Ser (abc) + AcCoA (de) → Cys (abc) + Ac (de)	0.0	1000.0
lldapsyn:	Asp (abcd) + Pyr (efg) + Glu (hijkl) + SucCoA (mnop) → LLDAP (abcdgfe) + aKG (hijkl) + Suc (mnop)	0.0	1000.0
lyssyn:	LLDAP (abcdefg) → Lys (abcdef) + CO_2 (g)	0.0	1000.0
thrsyn:	Asp (abcd) → Thr (abcd)	0.0	1000.0
metsyn:	Asp (abcd) + METHF (e) + Cys (fgh) + SucCoA (ijkl) → Met (abcde) + Pyr (fgh) + Suc (ijkl)	0.0	1000.0
valsyn:	Pyr (abc) + Pyr (def) + Glu (ghijk) → Val (abcef) + CO_2 (d) + aKG (ghijk)	0.0	1000.0
leusynI:	Pyr (cde) + Pyr (fgh) + Glu (ijklm) → Leu1 (dghe) + CO_2 (c) + CO_2 (f) + aKG (ijklm)	0.0	1000.0
leusynII:	AcCoA (ab) + Leu1 (dghe) → Leu (abdghe)	0.0	1000.0
ilesyn:	Thr (abcd) + Pyr (efg) + Glu (hijkl) → Ile (abfcdg) + CO_2 (e) + aKG (hijkl)	0.0	1000.0
phesynI:	PEP (def) + E4P (ghij) + Glu (klmno) → Phe1 (efghij) + CO_2 (d) + aKG (klmno)	0.0	1000.0
phesynII:	PEP (abc) + Phe1 (efghij) → Phe (abcefghij)	0.0	1000.0
tyrsynI:	PEP (def) + E4P (ghij) + Glu (klmno) → Tyr1 (efghij) + CO_2 (d) + aKG (klmno)	0.0	1000.0
tyrsynII:	PEP (abc) + Tyr1 (efghij) → Tyr (abcefghij)	0.0	1000.0
trpsynI:	R5P (defgh) + E4P (lmno) + PEP (pqr) + Gln (stuvw) → Trp1 (edlmno) + GAP (fgh) + Pyr (pqr) + Glu (stuvw)	0.0	1000.0
trpsynII:	PEP (ijk) + Trp1 (edlmno) → Trp2 (edklmnoj) + CO_2 (i)	0.0	1000.0
trpsynIII:	Ser (abc) + Trp2 (edklmnoj) → Trp (abcedklmnoj)	0.0	1000.0
hissyn:	R5P (abcde) + FTHF (f) + Gln (ghijk) + Asp (lmno) → His (edcbaf) + aKG (ghijk) + Fum (lmno)	0.0	1000.0

ONE CARBON METABOLISM

Reaction Name	Reaction Description	Flux LB	Flux UB
methfsyn:	MEETHF (a) → METHF (a)	0.0	1000.0
fthfsyn:	MEETHF (a) → FTHF (a)	0.0	1000.0

BIOMASS SYNTHESIS

Reaction Name	Reaction Description	Flux LB	Flux UB

Biomass:	0.488 Ala (abc) + 0.281 Arg (abcdef) + 0.229 Asn (abcd) + 0.229 Asp (abcd) + 0.087 Cys (abc) + 0.250 Glu (abcde) + 0.250 Gln (abcde) + 0.582 Gly (ab) + 0.090 His (abcdef) + 0.276 Ile (abcdef) + 0.428 Leu (abcdef) + 0.326 Lys (abcdef) + 0.146 Met (abcde) + 0.176 Phe (abcdefghi) + 0.210 Pro (abcde) + 0.205 Ser (abc) + 0.241 Thr (abcd) + 0.054 Trp (abcdefghijk) + 0.131 Tyr (abcdefghi) + 0.402 Val (abcde) + 0.205 G6P (abcdef) + 0.071 F6P (abcdef) + 0.754 R5P (abcde) + 0.129 GAP (abc) + 0.619 3PG (abc) + 0.051 PEP (abc) + 0.083 Pyr (abc) + 2.510 AcCoA (ab) + 0.087 aKG (abcde) + 0.340 OAA (abcd) + 0.443 MEETHF (a) →	0.015	0.015

22

Results

The *E. coli* model was fit to simulated liquid chromatography-tandem mass spectrometry (LC-MS/MS) data with random noise added to the measurements to simulate experimental error. This data is listed in **S1 Data**. The simulated LC-MS/MS data includes the parent and daughter ions described by Rühl et al [2].

All pool sizes were constrained by a lower bound of 0.001 and an upper bound of 10.0 when fitting the model. The time domain was discretized by assigning time nodes at values of 0.0, 0.015, 0.045, 0.150, 0.400, 1.100, and 3.100. The 3rd-order Radau IIA collocation method was used initially to identify the optimum, followed by the 9th-order Radau IIA method for refining the solution.

GLYCOLYSIS

Reaction Name	Actual Flux	Actual Exchange	Predicted Flux	SD	Predicted Exchange	SD
gl-in:	1.000		1.000	0.000		
gl-1:	1.000		1.000	0.000		
gl-2:	0.737		0.735	0.029		
gl-3:	0.875	0.018	0.872	0.034	0.030	0.012
gl-4:	0.875	79.945	0.872	0.034	49.635	352.884
gl-5:	0.875	398.604	0.872	0.034	999.128	456.952
gl-6:	1.851	387.675	1.847	0.034	998.153	303.376
gl-7:	1.851	177.631	1.847	0.034	0.000	290.339
gl-8:	1.825	16.921	1.827	0.034	998.173	479.101
gl-9:	1.652		1.667	0.043		

PENTOSE PHOSPHATE PATHWAY

Reaction Name	Actual Flux	Actual Exchange	Predicted Flux	SD	Predicted Exchange	SD
pp-1:	0.260		0.262	0.029		
pp-2:	0.226		0.222	0.010		
pp-3:	0.086	3.213	0.085	0.003	3.285	1.189
pp-4:	0.140	1.316	0.137	0.007	1.324	0.107
pp-5:	0.072	0.393	0.071	0.003	0.386	0.020
pp-6:	0.072	0.000	0.071	0.003	0.001	0.001
pp-7:	0.067	0.000	0.066	0.003	0.000	0.002

ED PATHWAY

Reaction Name	Actual Flux	Actual Exchange	Predicted Flux	SD	Predicted Exchange	SD
ed-1:	0.035		0.040	0.037		
ed-2:	0.035		0.040	0.037		

TCA CYCLE

Reaction Name	Actual Flux	Actual Exchange	Predicted Flux	SD	Predicted Exchange	SD
pd:	1.785		1.778	0.007		
tc-1:	0.096		0.089	0.011		
tc-2:	0.096		0.089	0.011		
tc-3:	0.077		0.072	0.010		
tc-4:	0.060		0.056	0.010		

tc-5:	0.053		0.049	0.010		
tc-6:	0.080	646.735	0.073	0.011	20.146	493.971
tc-7:	0.086	0.419	0.078	0.011	0.424	0.092
tc-8:	-0.033	1000.000	-0.016	0.014	111.089	475.008

ANAPLEROTIC/CATAPLEROTIC REACTIONS

Reaction Name	Actual Flux	Actual Exchange	Predicted Flux	SD	Predicted Exchange	SD
me:	0.138		0.111	0.018		
an:	0.161	0.000	0.148	0.020	0.000	0.010

ACETATE SYNTHESIS

Reaction Name	Actual Flux	Actual Exchange	Predicted Flux	SD	Predicted Exchange	SD
ac:	1.618		1.631	0.009		

GLYOXYLATE CYCLE

Reaction Name	Actual Flux	Actual Exchange	Predicted Flux	SD	Predicted Exchange	SD
gx-1:	0.019		0.017	0.003		
gx-2:	0.019		0.017	0.003		

EXCHANGE FLUXES

Reaction Name	Actual Flux	Actual Exchange	Predicted Flux	SD	Predicted Exchange	SD
co2-in:	-2.154	0.524	-2.126	0.018	0.585	0.095
ac-out:	1.625		1.639	0.009		

AMINO ACID SYNTHESIS

Reaction Name	Actual Flux	Actual Exchange	Predicted Flux	SD	Predicted Exchange	SD
glusyn:	0.098		0.103	0.007		
glnsyn:	0.010		0.010	0.000		
prosyn:	0.003		0.003	0.000		
argsyn:	0.004		0.004	0.000		
aspsyn:	0.027		0.038	0.013		
asnsyn:	0.003		0.003	0.000		
alasyn:	0.007		0.007	0.000		
sersyn:	0.017		0.012	0.007		
glysyn:	0.009	0.005	0.004	0.007	999.996	280.146
glydeg:	0.001	0.000	0.006	0.007	999.994	338.953
thrdeg:	0.000		0.011	0.013		
cyssyn:	0.003		0.003	0.000		
lldapsyn:	0.005		0.005	0.000		
lyssyn:	0.005		0.005	0.000		
thrsyn:	0.008		0.018	0.013		
metsyn:	0.002		0.002	0.000		
valsyn:	0.006		0.006	0.000		
leusynI:	0.006		0.006	0.000		
leusynII:	0.006		0.006	0.000		

ilesyn:	0.004		0.004	0.000		
phesynI:	0.003		0.003	0.000		
phesynII:	0.003		0.003	0.000		
tyrsynI:	0.002		0.002	0.000		
tyrsynII:	0.002		0.002	0.000		
trpsynI:	0.001		0.001	0.000		
trpsynII:	0.001		0.001	0.000		
trpsynIII:	0.001		0.001	0.000		
hissyn:	0.001		0.001	0.000		

ONE CARBON METABOLISM

Reaction Name	Actual Flux	Actual Exchange	Predicted Flux	SD	Predicted Exchange	SD
methfsyn:	0.002		0.002	0.000		
fthfsyn:	0.001		0.001	0.000		

BIOMASS SYNTHESIS

Reaction Name	Actual Flux	Actual Exchange	Predicted Flux	SD	Predicted Exchange	SD
Biomass:	0.015		0.015	0.000		

Pool Name	Actual Pool Size	Predicted Pool Size	SD
Glc	0.096	2.700	0.212
G6P	0.085	0.035	0.009
F6P	0.061	0.066	0.007
FBP	0.089	0.171	0.059
DHAP	0.012	0.001	0.097
GAP	0.006	0.001	0.034
BPG	0.075	0.001	0.009
3PG	0.032	0.015	0.051
PEP	0.000	0.001	0.026
6PG	0.050	0.087	0.010
Ru5P	0.086	0.001	0.010
R5P	0.076	0.021	0.011
X5P	0.091	0.079	0.009
S7P	0.043	0.089	0.005
E4P	0.065	0.048	0.008
KDPG	0.008	0.033	1.955
Pyr	0.059	0.031	0.012
OAA	0.045	0.022	0.047
AcCoA	0.041	0.087	0.026
Cit	0.057	0.080	0.010
iCit	0.025	0.042	0.006
aKG	0.041	0.024	0.007

SucCoA	0.048	0.066	0.025
Suc	0.029	0.105	0.030
Fum	0.087	0.001	0.020
Mal	0.074	0.079	0.049
CO2	0.034	0.001	0.037
Gox	0.040	10.000	4.673
Ac	0.048	0.086	0.069
Glu	0.082	0.053	0.004
Gln	0.025	0.001	1.468
Asp	0.048	0.045	0.016
Ser	0.014	0.001	1.330
Gly	0.009	0.001	1.307
MEETHF	0.016	10.000	3.896
Thr	0.078	10.000	3.722
LLDAP	0.100	10.000	4.854
METHF	0.090	0.768	0.367
Cys	0.012	0.001	3.956
Leu1	0.030	9.948	0.151
Phe1	0.041	0.491	1.562
Tyr1	0.031	6.652	0.000
Trp1	0.015	8.508	1.397
Trp2	0.009	1.375	1.119
FTHF	0.091	0.001	0.254
Ala	0.000	4.909	0.000
Arg	0.061	0.885	0.664
Asn	0.085	8.010	0.000
His	0.081	1.254	1.396
Ile	0.076	0.001	0.045
Leu	0.020	6.674	0.345
Lys	0.005	6.788	0.102
Met	0.034	4.390	0.000
Phe	0.017	8.980	0.414
Pro	0.008	6.372	0.311
Trp	0.078	0.001	0.004
Tyr	0.027	3.202	0.000
Val	0.046	2.173	0.688

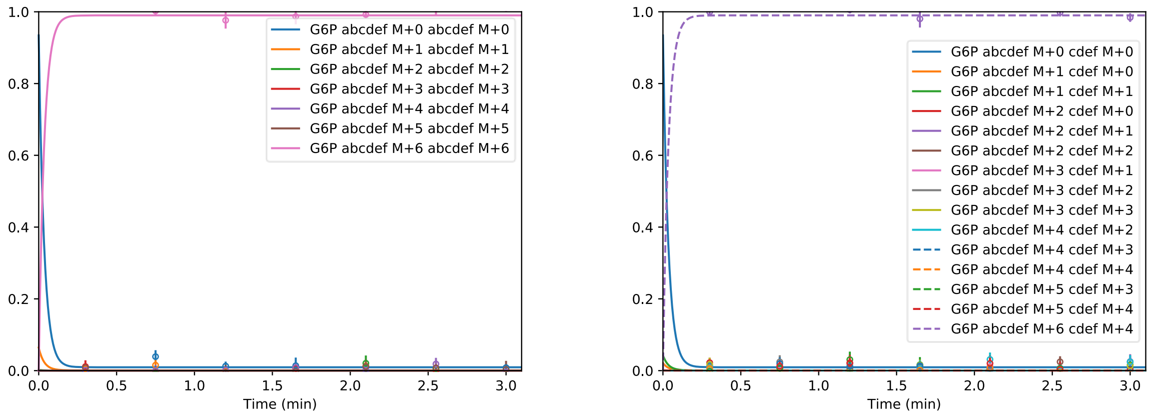
G6P abcdef M+0 abcdef M+0
G6P abcdef M+1 abcdef M+1
G6P abcdef M+2 abcdef M+2
G6P abcdef M+3 abcdef M+3
G6P abcdef M+4 abcdef M+4
G6P abcdef M+5 abcdef M+5
G6P abcdef M+6 abcdef M+6
Time (min)
G6P abcdef M+0 cdef M+0
G6P abcdef M+1 cdef M+0
G6P abcdef M+1 cdef M+1
G6P abcdef M+2 cdef M+0
G6P abcdef M+2 cdef M+1
G6P abcdef M+2 cdef M+2
G6P abcdef M+3 cdef M+1
G6P abcdef M+3 cdef M+2
G6P abcdef M+3 cdef M+3
G6P abcdef M+4 cdef M+2
G6P abcdef M+4 cdef M+3
G6P abcdef M+4 cdef M+4
G6P abcdef M+5 cdef M+3
G6P abcdef M+5 cdef M+4
G6P abcdef M+6 cdef M+4
Time (min)

37

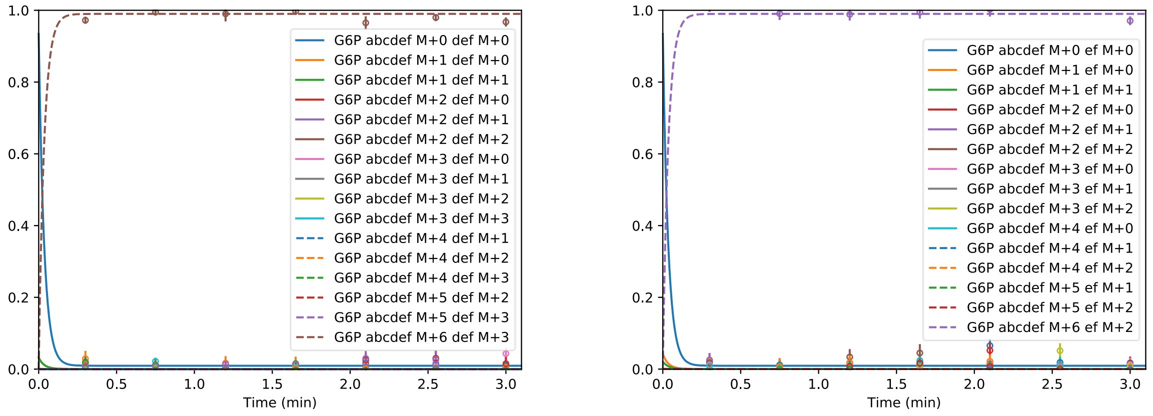
G6P abcdef M+0 def M+0
G6P abcdef M+1 def M+0
G6P abcdef M+1 def M+1
G6P abcdef M+2 def M+0
G6P abcdef M+2 def M+1
G6P abcdef M+2 def M+2
G6P abcdef M+3 def M+0
G6P abcdef M+3 def M+1
G6P abcdef M+3 def M+2
G6P abcdef M+3 def M+3
G6P abcdef M+4 def M+1
G6P abcdef M+4 def M+2
G6P abcdef M+4 def M+3
G6P abcdef M+5 def M+2
G6P abcdef M+5 def M+3
G6P abcdef M+6 def M+3
Time (min)
G6P abcdef M+0 ef M+0
G6P abcdef M+1 ef M+0
G6P abcdef M+1 ef M+1
G6P abcdef M+2 ef M+0
G6P abcdef M+2 ef M+1
G6P abcdef M+2 ef M+2
G6P abcdef M+3 ef M+0
G6P abcdef M+3 ef M+1
G6P abcdef M+3 ef M+2
G6P abcdef M+4 ef M+0
G6P abcdef M+4 ef M+1
G6P abcdef M+4 ef M+2
G6P abcdef M+5 ef M+1
G6P abcdef M+5 ef M+2
G6P abcdef M+6 ef M+2
Time (min)

38

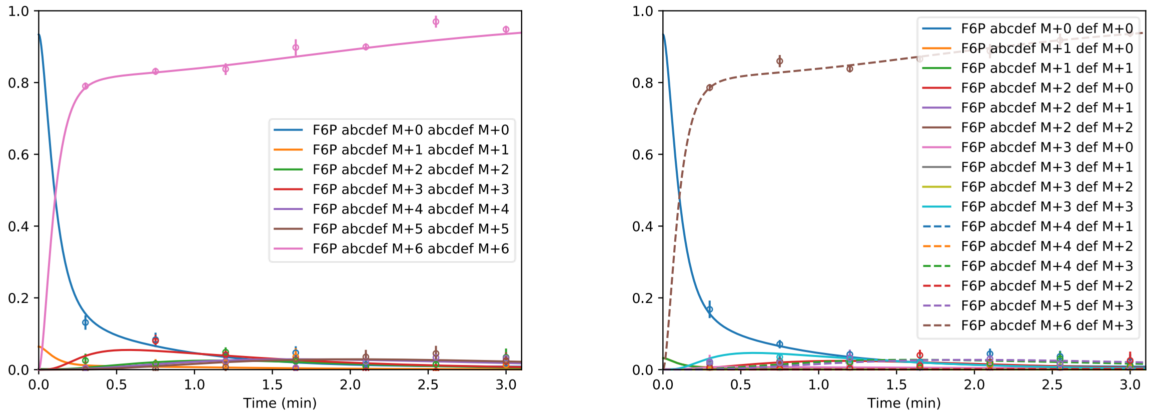
F6P abcdef M+0 abcdef M+0
F6P abcdef M+1 abcdef M+1
F6P abcdef M+2 abcdef M+2
F6P abcdef M+3 abcdef M+3
F6P abcdef M+4 abcdef M+4
F6P abcdef M+5 abcdef M+5
F6P abcdef M+6 abcdef M+6
Time (min)
F6P abcdef M+0 def M+0
F6P abcdef M+1 def M+0
F6P abcdef M+1 def M+1
F6P abcdef M+2 def M+0
F6P abcdef M+2 def M+1
F6P abcdef M+2 def M+2
F6P abcdef M+3 def M+0
F6P abcdef M+3 def M+1
F6P abcdef M+3 def M+2
F6P abcdef M+3 def M+3
F6P abcdef M+4 def M+1
F6P abcdef M+4 def M+2
F6P abcdef M+4 def M+3
F6P abcdef M+5 def M+2
F6P abcdef M+5 def M+3
F6P abcdef M+6 def M+3
Time (min)

39

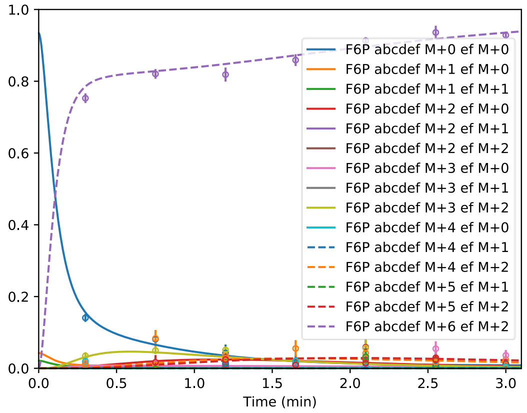
F6P abcdef M+0 ef M+0
F6P abcdef M+1 ef M+0
F6P abcdef M+1 ef M+1
F6P abcdef M+2 ef M+0
F6P abcdef M+2 ef M+1
F6P abcdef M+2 ef M+2
F6P abcdef M+3 ef M+0
F6P abcdef M+3 ef M+1
F6P abcdef M+3 ef M+2
F6P abcdef M+4 ef M+0
F6P abcdef M+4 ef M+1
F6P abcdef M+4 ef M+2
F6P abcdef M+5 ef M+1
F6P abcdef M+5 ef M+2
F6P abcdef M+6 ef M+2
Time (min)

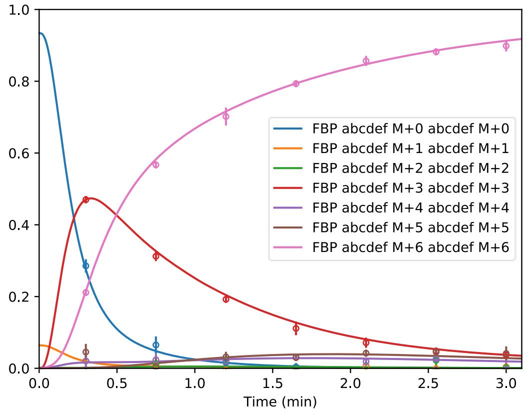
FBP abcdef M+0 abcdef M+0
FBP abcdef M+1 abcdef M+1
FBP abcdef M+2 abcdef M+2
FBP abcdef M+3 abcdef M+3
FBP abcdef M+4 abcdef M+4
FBP abcdef M+5 abcdef M+5
FBP abcdef M+6 abcdef M+6
Time (min)

40

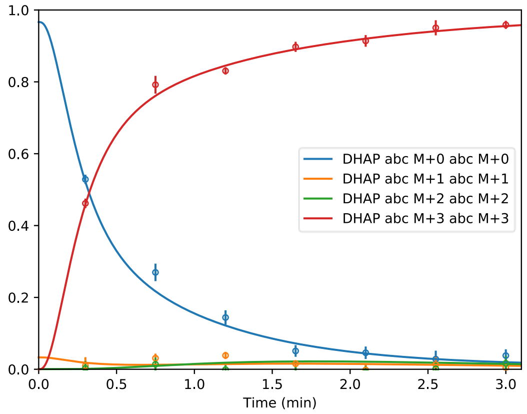
DHAP abc M+0 abc M+0
DHAP abc M+1 abc M+1
DHAP abc M+2 abc M+2
DHAP abc M+3 abc M+3
Time (min)

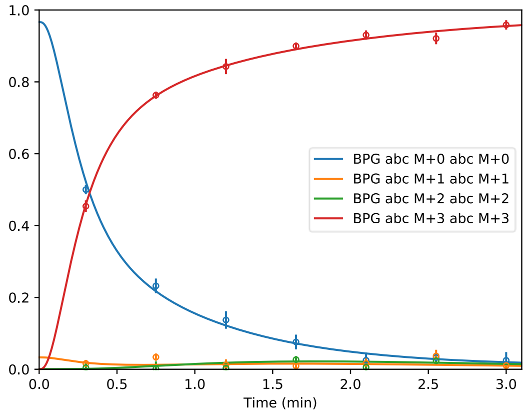
BPG abc M+0 abc M+0
BPG abc M+1 abc M+1
BPG abc M+2 abc M+2
BPG abc M+3 abc M+3
Time (min)

41

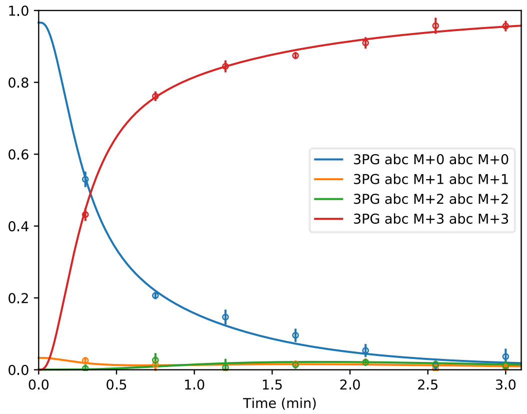
3PG abc M+0 abc M+0
3PG abc M+1 abc M+1
3PG abc M+2 abc M+2
3PG abc M+3 abc M+3
Time (min)

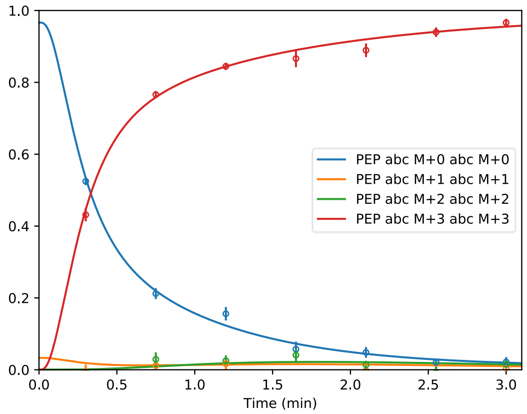
PEP abc M+0 abc M+0
PEP abc M+1 abc M+1
PEP abc M+2 abc M+2
PEP abc M+3 abc M+3
Time (min)

42

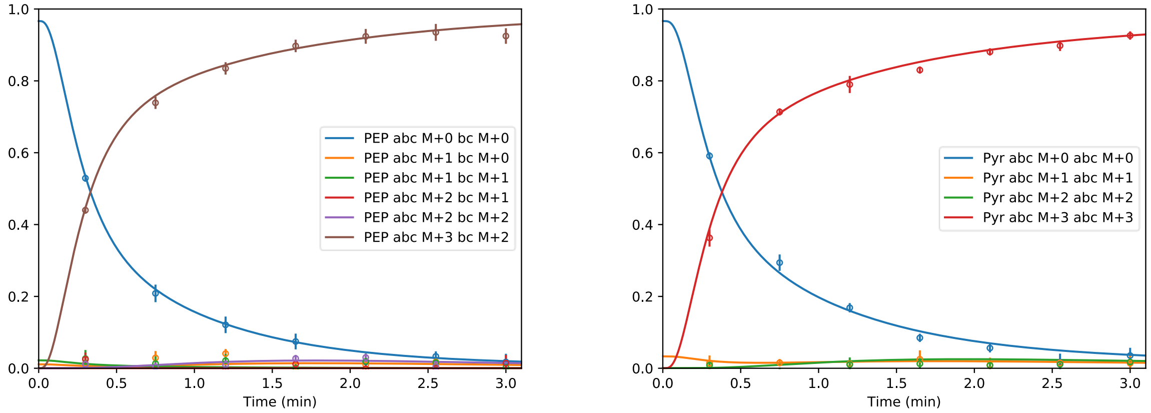
PEP abc M+0 bc M+0
PEP abc M+1 bc M+0
PEP abc M+1 bc M+1
PEP abc M+2 bc M+1
PEP abc M+2 bc M+2
PEP abc M+3 bc M+2
Time (min)
Pyr abc M+0 abc M+0
Pyr abc M+1 abc M+1
Pyr abc M+2 abc M+2
Pyr abc M+3 abc M+3
Time (min)

43

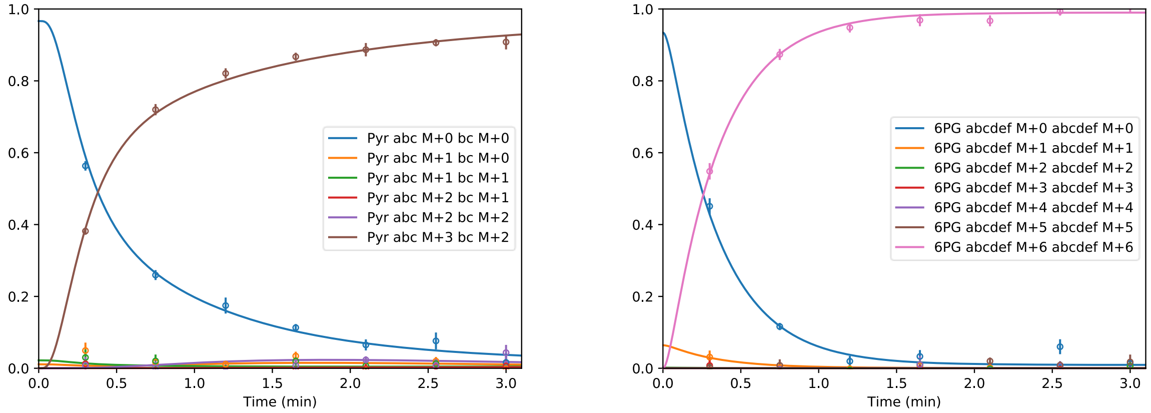
Pyr abc M+0 bc M+0
Pyr abc M+1 bc M+0
Pyr abc M+1 bc M+1
Pyr abc M+2 bc M+1
Pyr abc M+2 bc M+2
Pyr abc M+3 bc M+2
Time (min)
6PG abcdef M+0 abcdef M+0
6PG abcdef M+1 abcdef M+1
6PG abcdef M+2 abcdef M+2
6PG abcdef M+3 abcdef M+3
6PG abcdef M+4 abcdef M+4
6PG abcdef M+5 abcdef M+5
6PG abcdef M+6 abcdef M+6
Time (min)

44

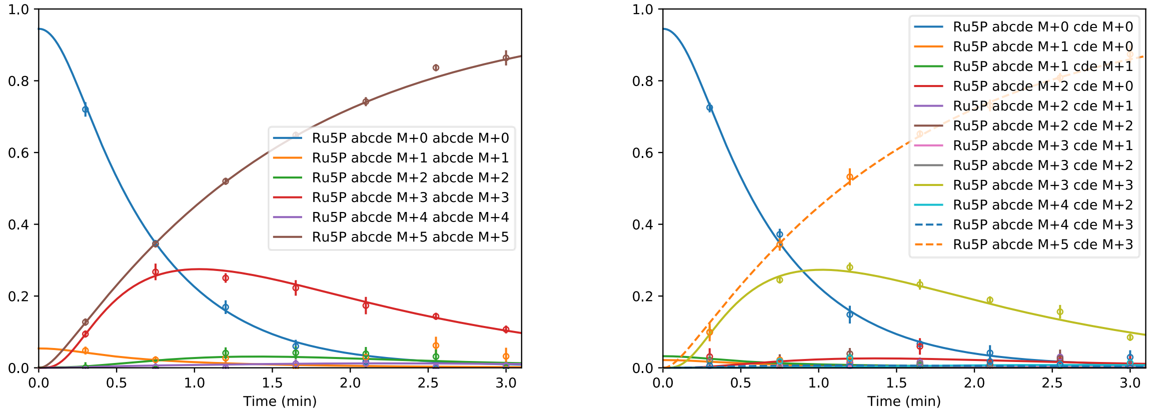
Ru5P abcde M+0 abcde M+0
Ru5P abcde M+1 abcde M+1
Ru5P abcde M+2 abcde M+2
Ru5P abcde M+3 abcde M+3
Ru5P abcde M+4 abcde M+4
Ru5P abcde M+5 abcde M+5
Time (min)
Ru5P abcde M+0 cde M+0
Ru5P abcde M+1 cde M+0
Ru5P abcde M+1 cde M+1
Ru5P abcde M+2 cde M+0
Ru5P abcde M+2 cde M+1
Ru5P abcde M+2 cde M+2
Ru5P abcde M+3 cde M+1
Ru5P abcde M+3 cde M+2
Ru5P abcde M+3 cde M+3
Ru5P abcde M+4 cde M+2
Ru5P abcde M+4 cde M+3
Ru5P abcde M+5 cde M+3
Time (min)

45

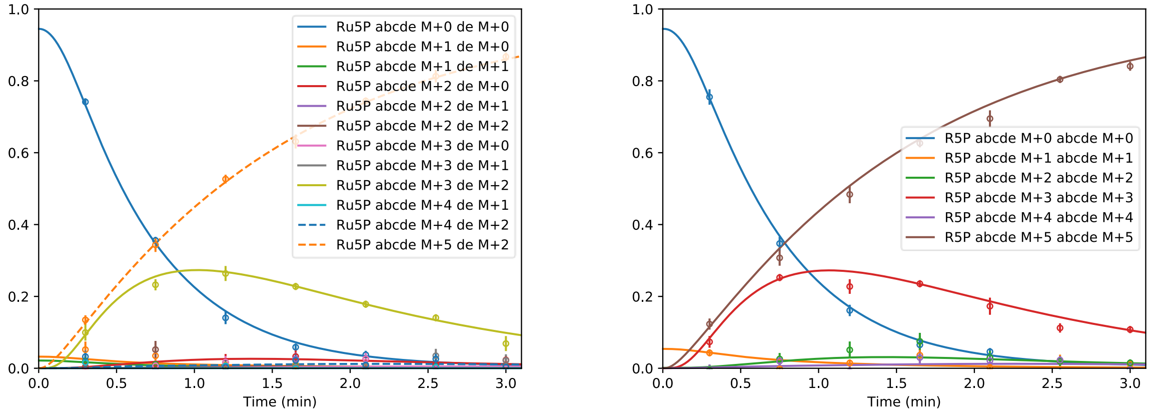
Ru5P abcde M+0 de M+0
Ru5P abcde M+1 de M+0
Ru5P abcde M+1 de M+1
Ru5P abcde M+2 de M+0
Ru5P abcde M+2 de M+1
Ru5P abcde M+2 de M+2
Ru5P abcde M+3 de M+0
Ru5P abcde M+3 de M+1
Ru5P abcde M+3 de M+2
Ru5P abcde M+4 de M+1
Ru5P abcde M+4 de M+2
Ru5P abcde M+5 de M+2
Time (min)
R5P abcde M+0 abcde M+0
R5P abcde M+1 abcde M+1
R5P abcde M+2 abcde M+2
R5P abcde M+3 abcde M+3
R5P abcde M+4 abcde M+4
R5P abcde M+5 abcde M+5
Time (min)

46

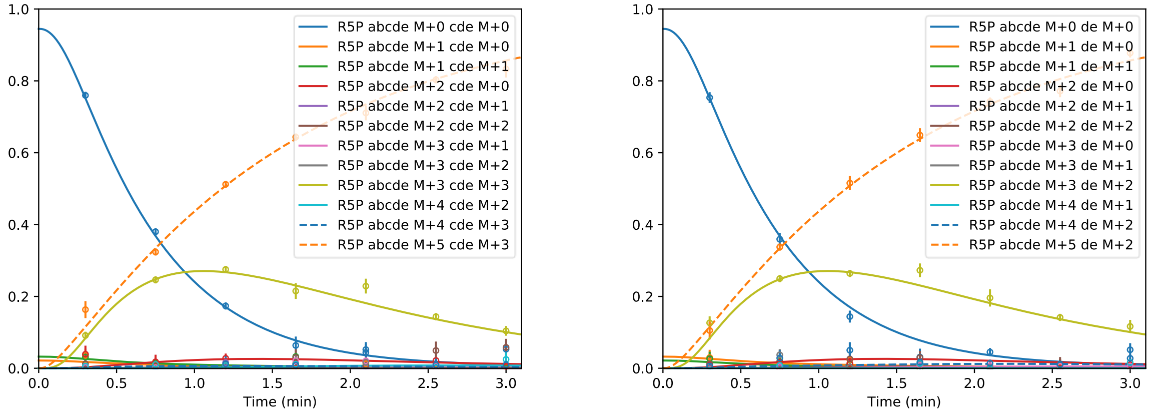
R5P abcde M+0 cde M+0
R5P abcde M+1 cde M+0
R5P abcde M+1 cde M+1
R5P abcde M+2 cde M+0
R5P abcde M+2 cde M+1
R5P abcde M+2 cde M+2
R5P abcde M+3 cde M+1
R5P abcde M+3 cde M+2
R5P abcde M+3 cde M+3
R5P abcde M+4 cde M+2
R5P abcde M+4 cde M+3
R5P abcde M+5 cde M+3
Time (min)
R5P abcde M+0 de M+0
R5P abcde M+1 de M+0
R5P abcde M+1 de M+1
R5P abcde M+2 de M+0
R5P abcde M+2 de M+1
R5P abcde M+2 de M+2
R5P abcde M+3 de M+0
R5P abcde M+3 de M+1
R5P abcde M+3 de M+2
R5P abcde M+4 de M+1
R5P abcde M+4 de M+2
R5P abcde M+5 de M+2
Time (min)

47

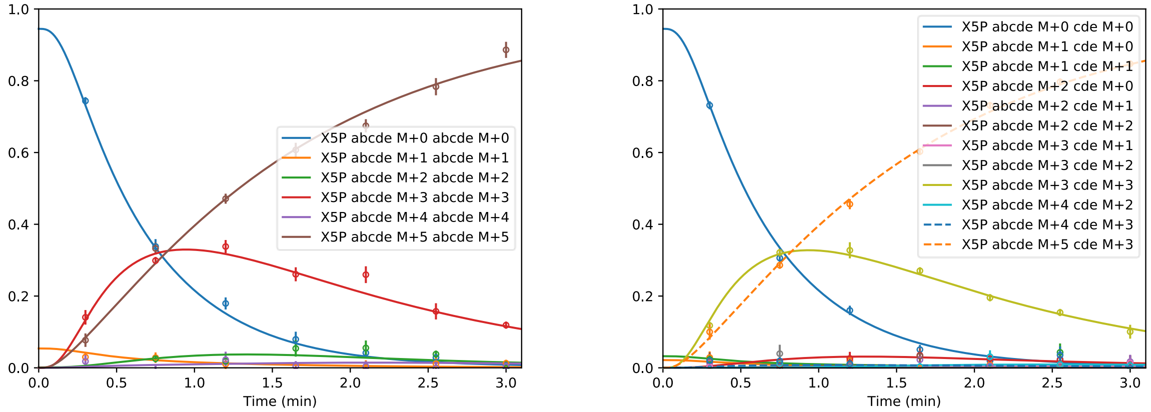
X5P abcde M+0 abcde M+0
X5P abcde M+1 abcde M+1
X5P abcde M+2 abcde M+2
X5P abcde M+3 abcde M+3
X5P abcde M+4 abcde M+4
X5P abcde M+5 abcde M+5
Time (min)
X5P abcde M+0 cde M+0
X5P abcde M+1 cde M+0
X5P abcde M+1 cde M+1
X5P abcde M+2 cde M+0
X5P abcde M+2 cde M+1
X5P abcde M+2 cde M+2
X5P abcde M+3 cde M+1
X5P abcde M+3 cde M+2
X5P abcde M+3 cde M+3
X5P abcde M+4 cde M+2
X5P abcde M+4 cde M+3
X5P abcde M+5 cde M+3
Time (min)

48

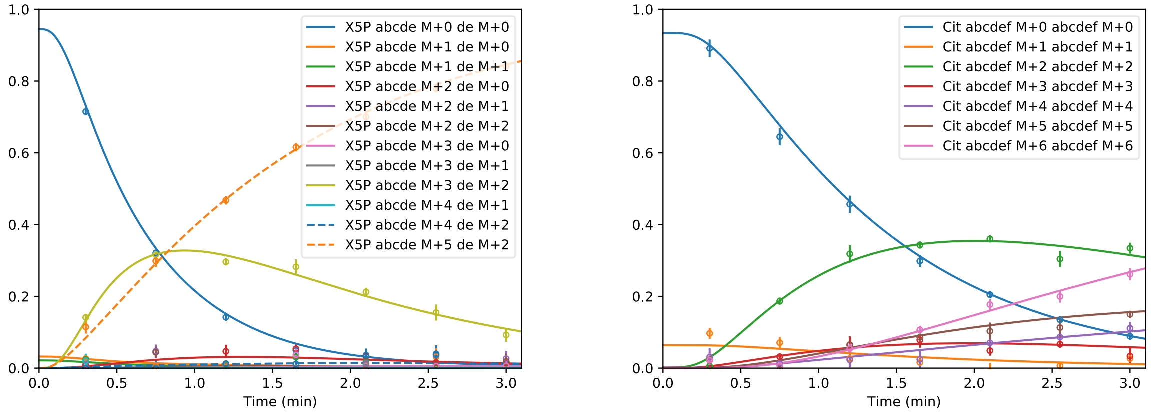
X5P abcde M+0 de M+0
X5P abcde M+1 de M+0
X5P abcde M+1 de M+1
X5P abcde M+2 de M+0
X5P abcde M+2 de M+1
X5P abcde M+2 de M+2
X5P abcde M+3 de M+0
X5P abcde M+3 de M+1
X5P abcde M+3 de M+2
X5P abcde M+4 de M+1
X5P abcde M+4 de M+2
X5P abcde M+5 de M+2
Time (min)
Cit abcdef M+0 abcdef M+0
Cit abcdef M+1 abcdef M+1
Cit abcdef M+2 abcdef M+2
Cit abcdef M+3 abcdef M+3
Cit abcdef M+4 abcdef M+4
Cit abcdef M+5 abcdef M+5
Cit abcdef M+6 abcdef M+6
Time (min)

49

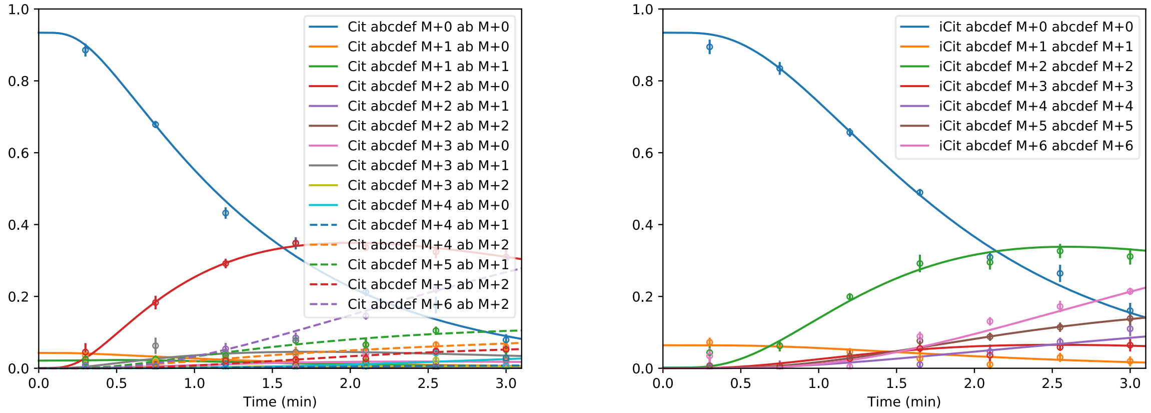
Cit abcdef M+0 ab M+0
Cit abcdef M+1 ab M+0
Cit abcdef M+1 ab M+1
Cit abcdef M+2 ab M+0
Cit abcdef M+2 ab M+1
Cit abcdef M+2 ab M+2
Cit abcdef M+3 ab M+0
Cit abcdef M+3 ab M+1
Cit abcdef M+3 ab M+2
Cit abcdef M+4 ab M+0
Cit abcdef M+4 ab M+1
Cit abcdef M+4 ab M+2
Cit abcdef M+5 ab M+1
Cit abcdef M+5 ab M+2
Cit abcdef M+6 ab M+2
Time (min)
iCit abcdef M+0 abcdef M+0
iCit abcdef M+1 abcdef M+1
iCit abcdef M+2 abcdef M+2
iCit abcdef M+3 abcdef M+3
iCit abcdef M+4 abcdef M+4
iCit abcdef M+5 abcdef M+5
iCit abcdef M+6 abcdef M+6
Time (min)

50

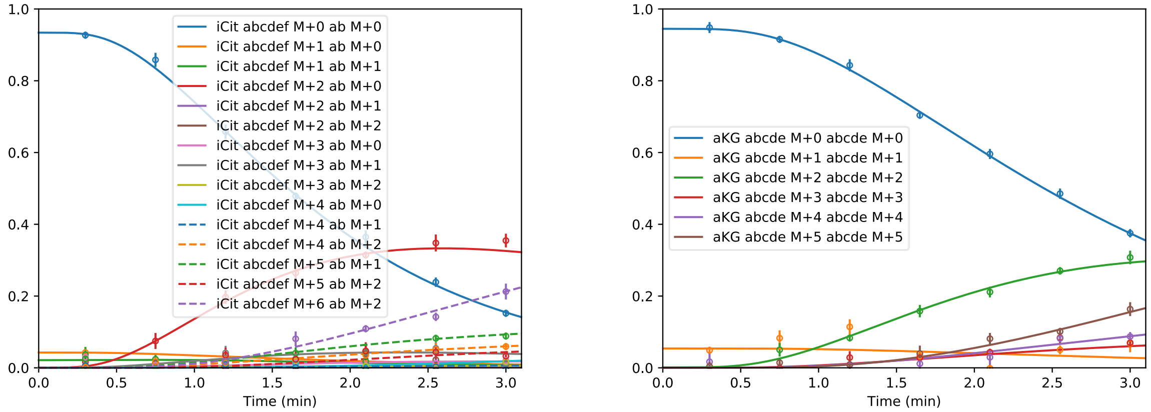
iCit abcdef M+0 ab M+0
iCit abcdef M+1 ab M+0
iCit abcdef M+1 ab M+1
iCit abcdef M+2 ab M+0
iCit abcdef M+2 ab M+1
iCit abcdef M+2 ab M+2
iCit abcdef M+3 ab M+0
iCit abcdef M+3 ab M+1
iCit abcdef M+3 ab M+2
iCit abcdef M+4 ab M+0
iCit abcdef M+4 ab M+1
iCit abcdef M+4 ab M+2
iCit abcdef M+5 ab M+1
iCit abcdef M+5 ab M+2
iCit abcdef M+6 ab M+2
Time (min)
aKG abcde M+0 abcde M+0
aKG abcde M+1 abcde M+1
aKG abcde M+2 abcde M+2
aKG abcde M+3 abcde M+3
aKG abcde M+4 abcde M+4
aKG abcde M+5 abcde M+5
Time (min)

51

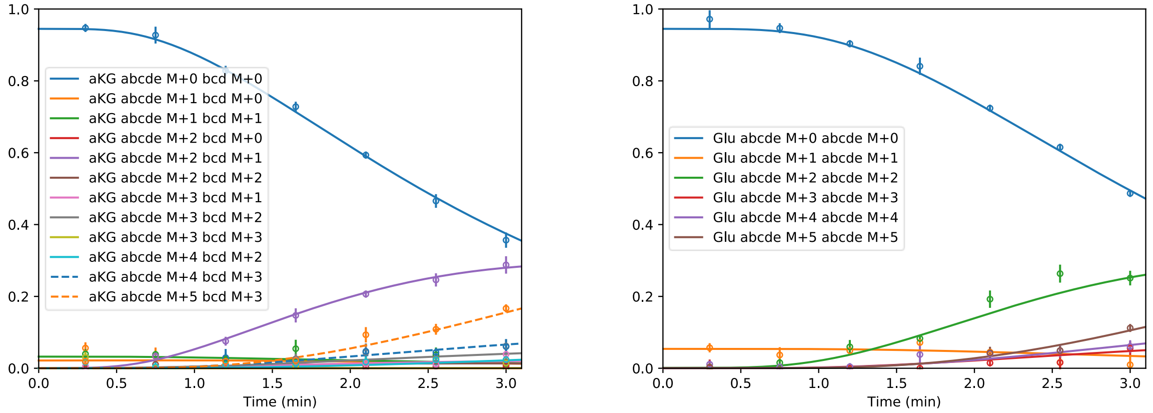
aKG abcde M+0 bcd M+0
aKG abcde M+1 bcd M+0
aKG abcde M+1 bcd M+1
aKG abcde M+2 bcd M+0
aKG abcde M+2 bcd M+1
aKG abcde M+2 bcd M+2
aKG abcde M+3 bcd M+1
aKG abcde M+3 bcd M+2
aKG abcde M+3 bcd M+3
aKG abcde M+4 bcd M+2
aKG abcde M+4 bcd M+3
aKG abcde M+5 bcd M+3
Time (min)
Glu abcde M+0 abcde M+0
Glu abcde M+1 abcde M+1
Glu abcde M+2 abcde M+2
Glu abcde M+3 abcde M+3
Glu abcde M+4 abcde M+4
Glu abcde M+5 abcde M+5
Time (min)

52

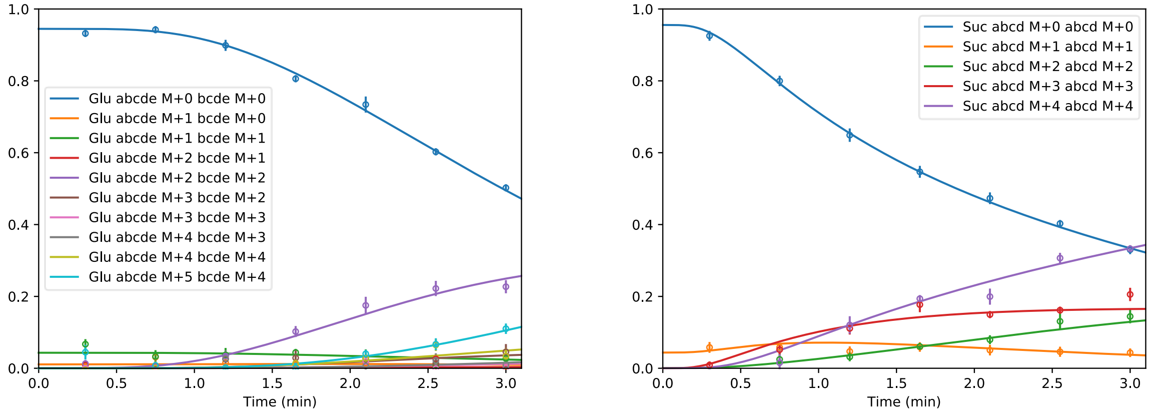
Glu abcde M+0 bcde M+0
Glu abcde M+1 bcde M+0
Glu abcde M+1 bcde M+1
Glu abcde M+2 bcde M+1
Glu abcde M+2 bcde M+2
Glu abcde M+3 bcde M+2
Glu abcde M+3 bcde M+3
Glu abcde M+4 bcde M+3
Glu abcde M+4 bcde M+4
Glu abcde M+5 bcde M+4
Time (min)
Suc abcd M+0 abcd M+0
Suc abcd M+1 abcd M+1
Suc abcd M+2 abcd M+2
Suc abcd M+3 abcd M+3
Suc abcd M+4 abcd M+4
Time (min)

53

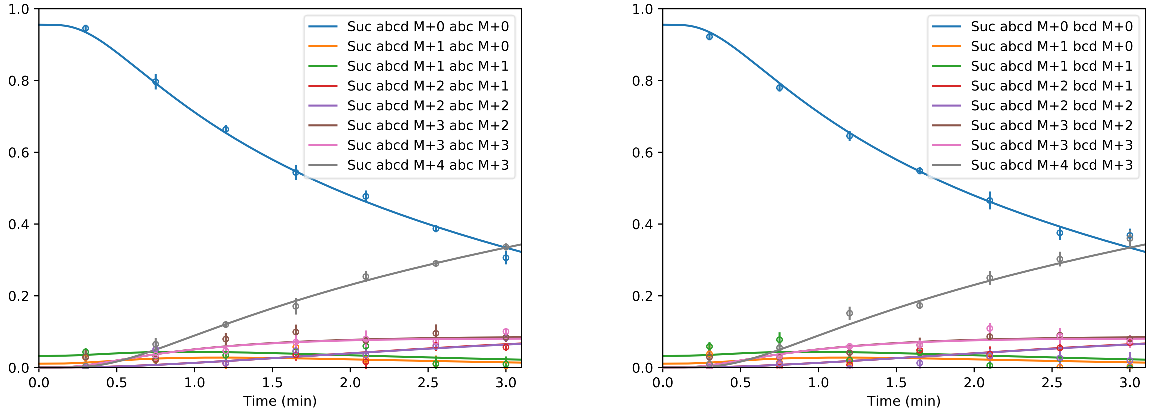
Suc abcd M+0 abc M+0
Suc abcd M+1 abc M+0
Suc abcd M+1 abc M+1
Suc abcd M+2 abc M+1
Suc abcd M+2 abc M+2
Suc abcd M+3 abc M+2
Suc abcd M+3 abc M+3
Suc abcd M+4 abc M+3
Time (min)
Suc abcd M+0 bcd M+0
Suc abcd M+1 bcd M+0
Suc abcd M+1 bcd M+1
Suc abcd M+2 bcd M+1
Suc abcd M+2 bcd M+2
Suc abcd M+3 bcd M+2
Suc abcd M+3 bcd M+3
Suc abcd M+4 bcd M+3
Time (min)

54

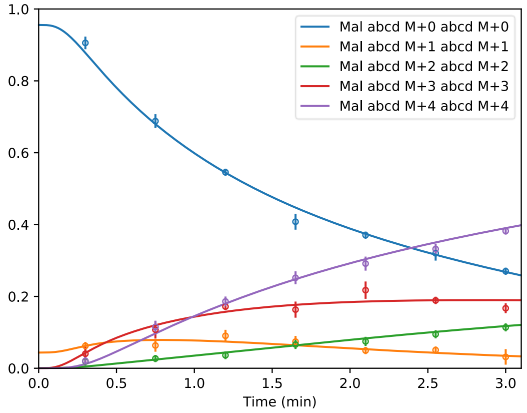
Mal abcd M+0 abcd M+0
Mal abcd M+1 abcd M+1
Mal abcd M+2 abcd M+2
Mal abcd M+3 abcd M+3
Mal abcd M+4 abcd M+4
Time (min)

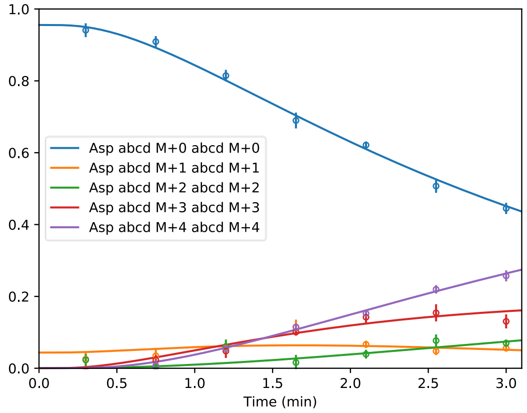
Asp abcd M+0 abcd M+0
Asp abcd M+1 abcd M+1
Asp abcd M+2 abcd M+2
Asp abcd M+3 abcd M+3
Asp abcd M+4 abcd M+4
Time (min)

55

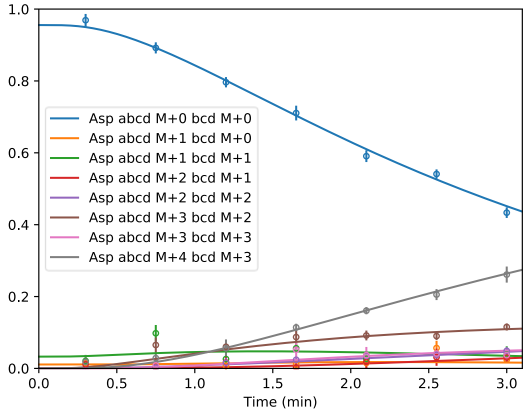
Asp abcd M+0 bcd M+0
Asp abcd M+1 bcd M+0
Asp abcd M+1 bcd M+1
Asp abcd M+2 bcd M+1
Asp abcd M+2 bcd M+2
Asp abcd M+3 bcd M+2
Asp abcd M+3 bcd M+3
Asp abcd M+4 bcd M+3
Time (min)

56

References

1. Young JD, Walther JL, Antoniewicz MR, Yoo H, Stephanopoulos G. An elementary metabolite unit (EMU) based method of isotopically nonstationary flux analysis. Biotechnol Bioeng. 2008;99: 686–699. doi:10/crmdfw

2. Rühl M, Rupp B, Nöh K, Wiechert W, Sauer U, Zamboni N. Collisional fragmentation of central carbon metabolites in LC-MS/MS increases precision of 13C metabolic flux analysis. Biotechnol Bioeng. 2012;109: 763–771. doi:10/bt7qxt
