# Supplementary material for: Isotope-assisted metabolic flux analysis as an equality-constrained nonlinear program for improved scalability and robustness
Source: PLoS Comput Biol. 2022 Mar 24;18(3):e1009831. doi: 10.1371/journal.pcbi.1009831 (PMC8947808; doi:10.1371/journal.pcbi.1009831)
Supplement: S5 Text — (PDF) [file pcbi.1009831.s005.pdf]

Generation of Initial Feasible Fluxes and Pool Sizes in eiFlux

eiFlux generates random initial feasible flux (and pool size for inst-MFA) distributions to serve as starting points for the optimization for parameter estimation. eiFlux uses the following custom algorithm for generating these initial parameter values:

- The Python portion of eiFlux generates a set of initial feasible fluxes and pool sizes consistent with the network stoichiometry, flux bounds, and pool size bounds provided by the user. To generate random initial feasible flux distributions, we have implemented a custom algorithm. Briefly, an LP is assembled with constraints defined by the network stoichiometry and flux bounds. The objective function is a linear combination of the fluxes with random coefficients having values on the interval [-1, 1]. The solution of this LP is a point on a vertex or edge of the feasible flux space, which serves as the first initial flux distribution. To generate the next feasible flux distribution, the LP is solved again with a different set of random coefficients. A random, additive linear combination of this new flux distribution and the previous one is a point in the interior of the flux space, which serves as the second initial feasible flux distribution. This is repeated to generate each subsequent feasible flux distribution. Notably, this algorithm samples points in the interior of the flux space, and not just edges and vertices. Initial feasible pool sizes are randomly chosen values between the user-input lower and upper bounds.
- If the metabolic network or flux bounds supplied by the user are stoichiometrically infeasible, the LP has no solution and the program quits, returning an infeasibility message. In this case, the user must revise their model to ensure the network and user-input flux bounds are stoichiometrically feasible. The user can (and should) play safe and input wide flux bounds for all except carbon source input and measured fluxes.
- The Python portion of eiFlux supplies this feasible set of fluxes and pool sizes to GAMS. GAMS solves this model as an exactly-determined system by using the “Constrained Nonlinear System (CNS)” option in the CONOPT solver to generate a feasible initial starting point for the optimization that satisfies the equality constraints. This takes only a few seconds even for very large models.
- This initializes all of the variables in the NLP (state variables and parameters) at an **initial feasible point**. This initial point serves as the feasible starting point for the optimization. The NLP is then solved by minimizing the variance weighted sum-of-squared-residuals as the objective function while maintaining constraint feasibility throughout the solution.
