# Supplementary material for: Isotope-assisted metabolic flux analysis as an equality-constrained nonlinear program for improved scalability and robustness
Source: PLoS Comput Biol. 2022 Mar 24;18(3):e1009831. doi: 10.1371/journal.pcbi.1009831 (PMC8947808; doi:10.1371/journal.pcbi.1009831)
Supplement: S6 Text — (PDF) [file pcbi.1009831.s006.pdf]

List of Symbols

Symbol	Description
z	Objective function value
m_n^{meas}	Value of isotope labeling measurement n
m_n	Value of simulated isotope labeling measurement n
σ_n	Standard deviation of isotope labeling measurement n
v_r^{meas}	Value of flux measurement r
v_r	Value of simulated flux measurement r
$\sigma_{v,r}$	Standard deviation of flux measurement r
x_i	Cumomer with index i
$Q_{rkij} x_i x_j$	Bimolecular reactions r producing x_k from x_i and x_j
$P_{rkj} x_i x_j$	Unimolecular production and consumption of x_k
$P_{rkj}^{inp} x_j^{inp}$	Input of fed isotopically labeled compound(s) into the network
$\mathbf{S}$	Stoichiometric matrix
R_{wr}	Element of the flux linear inequality constraint matrix with indices w and r
b_w	Flux inequality constraint bound with index w
h_c^{frag}	Fragment scaling factor for measured fragment with index c
h_n^{meas}	Measurement scaling factor or measurement with index n
$h_{c\mu}^{frag}$	Fragment scaling factor for measured fragment with index c at measurement time μ
$h_{n\mu}^{meas}$	Measurement scaling factor for measurement with index n at measurement time μ
$\mathbf{U}$	Matrix used to constrain each $h_{n\mu}^{meas}$ to equal its respective fragment scaling factor $h_{c\mu}^{frag}$
$\mathbf{M}$	Mapping matrix from cumomers to isotope labeling measurements
x_u	Zero-order cumomers (cumomers with indices u)
v_r	Flux with index r
v_r^{lb}	Lower bound of flux with index r
v_r^{ub}	Upper bound of flux with index r
p_k	Pool size of metabolite corresponding to cumomer k
$a_{\alpha\beta}$	Element of the Runge-Kutta matrix with indices α and β
b_α	Runge-Kutta weight with index α
c_α	Collocation node with index α on the interval [0,1]
h_γ	Width of time interval γ: $h_\gamma = t_\gamma - t_{\gamma-1}$
$x_{\gamma i}$	Value of cumomer with index i at time point γ

x_{0i}	Initial conditions of cumomer with index i
$k_{\alpha\gamma i}$	Basis coefficient with index α used for approximating $x_i(t)$ in time interval γ
$K_{\alpha\gamma i}$	Intermediate variable used to split the Runge-Kutta equations
$\ell_{\alpha}(\tau)$	Lagrange polynomial with index α
$B_{\alpha}(\tau)$	Basis function with index α for the collocation method
$m_{n\mu}^{meas}$	Value of isotope labeling measurement n at time point μ
$m_{n\mu}$	Value of simulated isotope labeling measurement n at time point μ
$p_m^{met,meas}$	Value of metabolite pool size measurement m
p_m^{met}	Value of simulated metabolite pool size m
$\mathbf{D}$	Matrix used to constrain each p_k to equal its respective metabolite pool size p_m^{met}
$p_m^{met,lb}$	Lower bound of metabolite pool size p_m^{met}
$p_m^{met,ub}$	Upper bound of metabolite pool size p_m^{met}
