# Supplementary figures and images for: Isotope-assisted metabolic flux analysis as an equality-constrained nonlinear program for improved scalability and robustness
Source: PLoS Comput Biol. 2022 Mar 24;18(3):e1009831. doi: 10.1371/journal.pcbi.1009831 (PMC8947808; doi:10.1371/journal.pcbi.1009831)

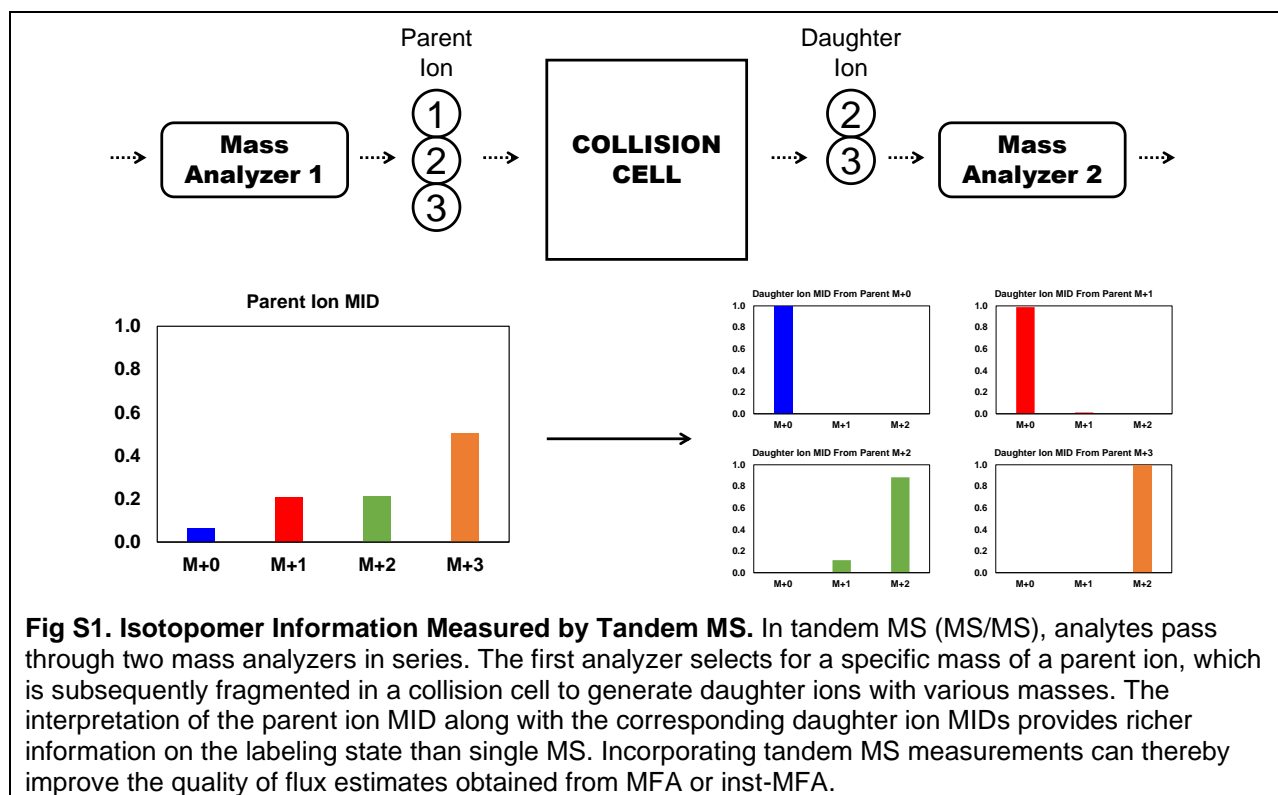

Supplement: S1 Fig — (PDF) [file pcbi.1009831.s007.pdf]
