# Supplementary material for: Isotope-assisted metabolic flux analysis as an equality-constrained nonlinear program for improved scalability and robustness
Source: PLoS Comput Biol. 2022 Mar 24;18(3):e1009831. doi: 10.1371/journal.pcbi.1009831 (PMC8947808; doi:10.1371/journal.pcbi.1009831)
Supplement: S1 File — The text files in these folders are used as inputs to eiFlux. The text file modelfile.txt is used to specify which model to run. The GAMS scripts used by eiFlux, in the folder GAMS_Model. A demo version of eiFlux that specifically runs two models listed above, thus fully reproducing the results in this manuscript. This is a compiled Python file, eiFlux_Limited.pyc. This demo version only runs only the two models listed above. Users may change model parameters such as the time nodes, but not the models themselves. After following the instructions given in the Installation_Instructions.pdf file, users may run the eiFlux_Limited.pyc file to run the software. (ZIP) [file pcbi.1009831.s009.zip › Installation_Instructions.pdf]

Installation Instructions

eiFlux is written using two languages, Python and GAMS. Python is a general-purpose high-level programming language. GAMS is a proprietary algebraic modeling language designed to efficiently implement solvers for large-scale optimization problems. The model arrays are assembled in Python, then read into a GAMS Data Exchange (GDX) file using the GAMS/Python API. Following the creation of the GDX file, the GAMS model is prompted to run in the Python script. Following completion of the run, a second GDX file is created, this time by GAMS, which is subsequently read by the Python script and the results displayed and printed to text files.

To run eiFlux, it is necessary to have Python 3 (version 3.6.5 or above) and GAMS (version 33.2.0 or above) with the CONOPT solver. The Python libraries NumPy (version 1.15.4 or above), SciPy (version 1.5.4 or above), and Matplotlib (version 2.2.2 or above) must also be installed. GAMS must have a valid license file which includes the Base Module and the CONOPT solver. The GAMS/Python API must also be installed, ensuring that the version and bitness of the Python API is the same as that of Python.

The local directory containing the eiFlux Python script should also contain a folder called "GAMS_Model". Therein should be two GAMS scripts: eiFlux_GAMS and eiFlux_GAMS_Instationary. eiFlux_GAMS is called when running steady-state data collected at isotope labeling steady state. eiFlux_GAMS_Instationary is called when fitting time series data.

This version of eiFlux runs only the two models described in the manuscript. A full set of instructions for developing and running models using eiFlux is provided at the following URL: https://github.com/SriramLabUMD/eiFlux_Instructions_Public

The Toy, *E. coli* tandem-MS, and genome scale Synechocystis models presented in the manuscript were solved using GAMS version 33.2.0 and CONOPT version 4.21 on a Dell Precision Tower 3620, with 16.0 GB of memory and an Intel Core i7-7700 CPU @ 3.60GHz processor running on a single core. The *E. coli* single-MS example model was solved using GAMS version 34.2.0 with CONOPT version 4.23 on a Microsoft Surface Pro 6 with 8.0 GB of memory and an Intel Core i5-8250U CPU @1.60 GHz processor running on a single core. This model was solved using INCA 2.0 on the same system for a direct comparison.
